# COVID-19 vaccination intention and vaccine characteristics influencing vaccination acceptance: a global survey of 17 countries

**DOI:** 10.1186/s40249-021-00900-w

**Published:** 2021-10-07

**Authors:** Li Ping Wong, Haridah Alias, Mahmoud Danaee, Jamil Ahmed, Abhishek Lachyan, Carla Zi Cai, Yulan Lin, Zhijian Hu, Si Ying Tan, Yixiao Lu, Guoxi Cai, Di Khanh Nguyen, Farhana Nishat Seheli, Fatma Alhammadi, Milkar D. Madhale, Muditha Atapattu, Tasmi Quazi-Bodhanya, Samira Mohajer, Gregory D. Zimet, Qinjian Zhao

**Affiliations:** 1 Department of Epidemiology and Health Statistics, Fujian Provincial Key Laboratory of Environment Factors and Cancer, School of Public Health, Fujian Medical University, Fuzhou, 350122 Fujian China; 2 Centre for Epidemiology and Evidence-Based Practice, Department of Social and Preventive Medicine, Faculty of Medicine, University of Malaya, 50603 Kuala Lumpur, Malaysia; 3 Department of Community Health Science, Muhammad Medical College, Mirpurkhas, Sindh 69000 Pakistan; 4 World Health Organization National Polio Surveillance Project (NPSP) Unit Belgaum World Customs Organization, Hindu Nagar, Tilakwadi, Belgaum, Karnataka 590006 India; 5 Leadership Institute for Global Health Transformation, Saw Swee Hock School of Public Health, National University of Singapore, 12 Science Drive, Singapore, 117549 Singapore; 6 Department of Public Health, Nagasaki University Graduate School of Biomedical Sciences, Nagasaki, 852-8523 Japan; 7 Department of Academic Affairs and Testing, Dong Nai Technology University, Dong Nai, Vietnam; 8 Institute of Epidemiology, Disease Control & Research (IEDCR), Mohakhali, Dhaka, 1212 Bangladesh; 9 Ministry of Health and Prevention (MOHAP), Sharjah, United Arab Emirates; 10 Vijaya College of Nursing, Belgaum, Ayodhya Nagar, Belgaum, Karnataka 590001 India; 11 Department of Nursing, Faculty of Allied Health Sciences, University of Peradeniya, Peradeniya, Sri Lanka; 12 Leadership Dialogue, 16 Elland Road, Manor Gardens, Durban, 4001 South Africa; 13 Nursing and Midwifery Care Research Center, Mashhad University of Medical Sciences, Mashhad, Iran; 14 Department of Pediatrics, School of Medicine, Indiana University, 410 W, 10th St., HS 1001, Indianapolis, IN 46202 USA; 15 State Key Laboratory of Molecular Vaccinology and Molecular Diagnostics, National Institute of Diagnostics and Vaccine Development in Infectious Diseases, School of Public Health, Xiamen University, Xiamen, Fujian China

**Keywords:** COVID-19 vaccine, Vaccination intention, Vaccine characteristics, Vaccination acceptance, Vaccine choice

## Abstract

**Background:**

The availability of various types of COVID-19 vaccines and diverse characteristics of the vaccines present a dilemma in vaccination choices, which may result in individuals refusing a particular COVID-19 vaccine offered, hence presenting a threat to immunisation coverage and reaching herd immunity. The study aimed to assess global COVID-19 vaccination intention, vaccine characteristics influencing vaccination acceptance and desirable vaccine characteristics influencing the choice of vaccines.

**Methods:**

An anonymous cross*-*sectional survey was conducted between 4 January and 5 March 2021 in 17 countries worldwide. Proportions and the corresponding 95% confidence intervals (*CI*) of COVID-19 vaccine acceptance and vaccine characteristics influencing vaccination acceptance were generated and compared across countries and regions. Multivariable logistic regression analysis was used to determine the factors associated with COVID-19 vaccine hesitancy.

**Results:**

Of the 19,714 responses received, 90.4% (95% *CI* 81.8–95.3) reported likely or extremely likely to receive COVID-19 vaccine. A high proportion of *likely* or *extremely likely* to receive the COVID-19 vaccine was reported in Australia (96.4%), China (95.3%) and Norway (95.3%), while a high proportion reported being *unlikely* or *extremely unlikely* to receive the vaccine in Japan (34.6%), the U.S. (29.4%) and Iran (27.9%). Males, those with a lower educational level and those of older age expressed a higher level of COVID-19 vaccine hesitancy. Less than two-thirds (59.7%; 95% *CI* 58.4–61.0) reported only being willing to accept a vaccine with an effectiveness of more than 90%, and 74.5% (95% *CI* 73.4–75.5) said they would accept a COVID-19 vaccine with minor adverse reactions. A total of 21.0% (95% *CI* 20.0–22.0) reported not accepting an mRNA vaccine and 51.8% (95% *CI* 50.3–53.1) reported that they would only accept a COVID-19 vaccine from a specific country‐of‐origin. Countries from the Southeast Asia region reported the highest proportion of not accepting mRNA technology. The highest proportion from Europe and the Americas would only accept a vaccine produced by certain countries. The foremost important vaccine characteristic influencing vaccine choice is adverse reactions (40.6%; 95% *CI* 39.3–41.9) of a vaccine and effectiveness threshold (35.1%; 95% *CI* 33.9–36.4).

**Conclusions:**

The inter-regional and individual country disparities in COVID-19 vaccine hesitancy highlight the importance of designing an efficient plan for the delivery of interventions dynamically tailored to the local population.

**Graphic Abstract:**

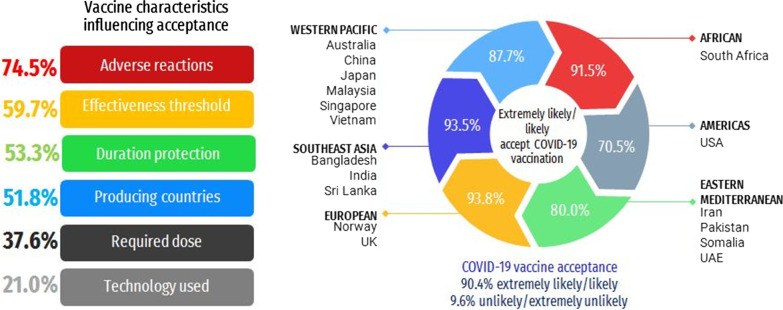

**Supplementary Information:**

The online version contains supplementary material available at 10.1186/s40249-021-00900-w.

## Background

Coronavirus disease 2019 (COVID-19), firstly reported in December 2019 [1], was declared a global pandemic by the World Health Organisation (WHO) on March 11, 2020 [2]. The novel coronavirus proliferated across the globe and has since become the greatest public health crisis the world has faced in over a century [3]. One year into the pandemic, as of early March 2021, there have been over 100 million global cases and over 2 million deaths reported [4]. Mass COVID-19 vaccination rollout is a public health top priority to mitigate the pandemic. The pandemic has motivated a global race in vaccine development initiatives which started as soon as the genetic sequence of SARS-CoV-2 was revealed. As of 2 March 2021, according to the WHO’s draft landscape of COVID-19 candidate vaccines, there were 76 candidate COVID-19 vaccines in clinical development and 182 in the preclinical evaluation stages [5]. Of significant importance to pandemic control, seven vaccines have been approved for full use and six for early or limited use across various countries as of 3 April 2021.

Vaccine hesitancy is a growing threat to global health security and the WHO named vaccine hesitancy as one of the top ten threats to global health in 2019 [6]. Despite the catastrophic impact of the pandemic and the enormous global effort to develop a vaccine as rapidly as possible, the COVID-19 vaccine is not spared from scepticism and hesitancy. Recently, COVID-19 vaccine hesitancy has been a subject of intense global interest. It is well known that the accelerated speed of the development as well as the fact that the vaccine is new has caused fear of its unknown safety and long-term side effects. The duration of protection of the current COVID-19 vaccines is also unknown. As the coronavirus mutates rapidly, new vaccines may need to be developed to combat more mutant strains of the coronavirus. Given the uncertainties surrounding the duration of protection and the possible need to be vaccinated against COVID-19 annually, similar to the seasonal flu vaccination, people may have an increased level of hesitancy towards a COVID-19 vaccination.

An important source of COVID-19 vaccine hesitancy that is not yet understood is the availability of various COVID-19 vaccines with different characteristics. Due to the global COVID-19 vaccine shortage, the public in many countries may not be able to choose one vaccine over another. People may be unwilling to get vaccinated if the COVID-19 vaccine offered in their country’s vaccination program is not their vaccine of choice. The current COVID-19 vaccines available differ in various characteristics such as level of efficacy for prevention of symptomatic disease, administration doses, manufacturing platforms, and effectiveness against virus variants [7]. Vaccine efficacies ranging from 50 to 95% have been reported [8]. It is unclear whether news headlines reporting certain COVID-19 vaccines offering greater than 90% effectiveness against COVID-19 while other vaccines having results of just over 50% effectiveness would influence a person favouring a certain vaccine over another.

In regards to administration doses, some of the COVID-19 vaccines will require two doses, while others just require one dose. The diverse manufacturing platforms of the COVID-19 vaccines also pose a challenge in vaccine choice. The public may lack confidence in vaccines developed using the messenger RNA (mRNA) technology over the traditional inactivated virus and recombinant protein platforms. The COVID-19 vaccines are being developed and produced by different manufacturers around the world. The country of manufacture of the COVID-19 vaccine may also be associated with hesitancy [9]. Distrust in vaccines from a specific country‐of‐origin has been reported [10–12]. The unprecedented speed of development and the rapid rollout of COVID-19 has also led some to believe, without evidence, that this is a result of skipping essential steps or being politically driven, leading to distrust in vaccines [11, 13–15].

The availability of several COVID-19 vaccines presents uncertainty on which vaccine to choose. Unwillingness to get vaccinated due to not favouring the COVID-19 vaccine offered in the country vaccination program can be the reason people refuse vaccination and may present a threat to achieving herd immunity. Therefore, understanding the vaccine characteristics influencing vaccine acceptance and choice of vaccine are important to inform effective strategies to improve vaccine uptake and coverage. A large-scale global study to evaluate the diverse COVID-19 vaccine characteristics influencing vaccination acceptance after the vaccine is available to the public is lacking. This multi-country survey aimed to assess (1) COVID-19 vaccination intentions and (2) vaccine characteristics influencing vaccination acceptance and choice. The vaccine characteristics investigated in this study are important factors expected to be associated with COVID-19 vaccine hesitancy (level of effectiveness, administration doses, adverse reactions, duration of protection, the new mRNA manufacturing platform, and country of the vaccine manufacturer).

## Methods

### Study design and participants

A purposive sample of researchers from various countries across all regions worldwide from the researchers’ academic linkages was invited to participate in this global survey. Researchers from a total of 17 countries responded to the invitation. Hence, a multi-country, cross-sectional survey was carried out in 17 countries using an online self-administered questionnaire during the period from 4 January to 6 March 2021. The 17 countries were grouped into six WHO regions: (1) African Region: South Africa; (2) Region of the Americas: United States of America; (3) South-East Asia Region: Bangladesh, India, and Sri Lanka; (4) European Region: Norway, and the United Kingdom; (5) Eastern Mediterranean Region: Iran, Pakistan, Somalia, and the United Arab Emirates; and (6) Western Pacific Region: Australia, China, Japan, Malaysia, Singapore, and Vietnam. The inclusion criteria were that individuals had to be 18 years or older, a citizen of the included countries, have not yet been vaccinated against COVID-19, and provide informed consent online.

A convenience sampling method was used in data collection. The sample size was calculated for each country using the formula: *n* = Z^2^ P(1 − P)/d^2^ [16]. Using a 0.05 margin of error with a 95% confidence intervals [*CI*] and 50% response distribution, the calculated sample size was 384. The sample size was multiplied by the predicted design effect of two to account for the use of convenience sampling and an online survey [17]. Hence, the minimum survey sample size for each country was set to 768 (384 × 2) participants.

The collaborators of all 17 countries were provided detailed information on the study and data collection strategies. Collaborators were informed as much as possible to distribute the survey link to the public of diverse cities in their country. Data collection was carried out using Google Forms and Qualtrics, distributed on social media platforms (repeated posting on Facebook, Twitter, WhatsApp and WeChat), online websites, and blogs in their countries. To increase response rates, a note encouraged survey respondents to share the survey links with their contact lists upon completion of the survey.

### Measures

Participants completed an online questionnaire (Additional file 1) on their (1) demographic background, (2) COVID-19 vaccination intention, (3) vaccine characteristics influencing acceptance, and (4) factors influencing the choices of COVID-19 vaccine. The questionnaire was developed in English. The native language option of the questions was available for surveys carried out in China, Vietnam, Sri Lanka, the United Arab Emirates, Malaysia and Japan. The items of the questions were content validated by content experts. Translation into target languages was carried out by standard forward–backward translation by native speakers. The translated questionnaire was also validated by new independent bilingual native speakers. The English and translated versions of the questionnaire were pilot tested in the respective countries before administration.

To ensure valid and reliable responses, we carried out survey data cleaning before analyses. Straightlining and duplicate responses were removed.

### Statistical analysis

Descriptive statistics were calculated for the sample demographic characteristics, COVID-19 vaccine acceptance, vaccine characteristics influencing vaccination acceptance and desirable vaccine characteristics influencing the choice of vaccines. Subsequently, we analysed the distribution of the overall responses by regions and by individual countries.

Due to large sample size disparities between the participating countries, in statistical analysis of the pooled responses from all 17 countries, the data were adjusted based on sample weight in order to reflect the population size of respective countries. Population size weights were employed in the analyses to ensure that each country is represented in proportion to its population size [18]. The population size weight is calculated as PWEIGHT = [Population size aged 15 years and above]/[(Study sample size in country) × 10 000]. The country population size and the study sample size for all countries used in the weightage are shown in Additional file 2.

Multivariable logistic regression analysis was used to determine the factors associated with COVID-19 vaccine hesitancy (1 = extremely unlikely/unlikely; 0 = likely/extremely likely to receive the COVID-19 vaccine) and vaccine characteristics influencing vaccination acceptance. Crude and population size weighted odds ratio (*OR*) with 95% *CI* was computed to determine the level of significance. Hosmer–Lemeshow goodness-of-fit tests were used to ensure that the models adequately fit the data. Statistical significance was established at a *p* value < 0.05. All analyses were also conducted using SPSS version 22.0 (SPSS Inc., Chicago, IL, USA).

### Ethical considerations

The principal investigator obtained ethical approval in conducting the survey in a global context from the University of Malaya Research Ethics Committee (UM.TNC2/UMREC-1182). Additional ethical approvals were also sought from the Institutional Review Board of Mehran University of Engineering and Technology (MUET.IRB-04/01-2021), Indiana University Human Research Protection Program (Protocol #: 10389) and Fujian Medical University, China (FJMU 2021 NO.63).

## Results

In total, 19,714 responses from 17 countries were received. The sample size of the participatory countries ranges from 776 (Sri Lanka) to 2175 (Malaysia). The demographics of the overall participants, the region of origin, and the descriptive responses to the survey questions on COVID-19 vaccination intention, vaccine characteristics influencing acceptance, and first and second choice of vaccine characteristics influencing a COVID-19 vaccine choice are listed in Table 1. Based on the results of analyses weighted by population, 53.8% of the study participants were female. Almost two-thirds of the participants (65.7%) had a university degree, and most were aged 18–49 years old (80.1%). The highest weighted prevalence of participation was from the Western Pacific (44.8%) and Southeast Asia (36.4%). Among the overall participants, 18.0% reported that they have ever delayed acceptance or refused any vaccine despite the availability of vaccination services in their countries. The demographics and descriptive responses to the survey questions by WHO regional category and individual 17 countries are detailed in Additional files 3 and 4, respectively.

**Table 1:** Description of overall study participants’ demographics, COVID-19 vaccination intention, and vaccine characteristics influencing vaccination acceptance and vaccine choice

		Participants *n* = 19,714 *n* (%)	Weighted prevalence % (95 *CI*)
Socio demography			
Age group, years			
18–29		5233 (26.5)	38.4 (37.1–39.6)
30–39		5524 (28.0)	25.4 (24.3–26.6)
40–49		4070 (20.6)	16.3 (15.4–17.2)
50–59		2751 (14.0)	12.0 (11.2–12.9)
60 and above		2136 (10.8)	7.9 (7.3–8.5)
Gender			
Male		9145 (46.4)	46.1 (44.8–47.4)
Female		10,557 (53.6)	53.8 (52.5–55.1)
Other		12 (0.1)	0.1 (0.1–0.2)
Highest education level			
Secondary school and below		2630 (13.3)	12.0 (11.2–12.9)
Certificate/A-Level/Diploma		4856 (24.6)	22.3 (21.2–23.4)
Bachelor degree		7883 (40.0)	42.5 (41.2–43.8)
Postgraduate degree		4345 (22.0)	23.2 (22.2–24.4)
WHO region^a^			
African		1086 (5.5)	1.3 (1.3–1.3)
Eastern Mediterranean		4122 (20.9)	7.1 (7.1–7.1)
European		2403 (12.2)	1.9 (1.9–1.9)
Region of the Americas		968 (4.9)	8.5 (8.5–8.5)
South-east Asia		3436 (17.4)	36.4 (36.4–36.4)
Western Pacific		7699 (39.1)	44.8 (44.8–44.8)
Ever delayed acceptance or refuse vaccine despite availability of vaccine service			
Yes		3812 (19.3)	18.0 (17.1–19)
No		15,902 (80.7)	82.0 (81–82.9)
COVID-19 vaccine acceptance			
Extremely likely		8395 (42.6)	47.2 (45.9–48.4)
Likely		8800 (44.6)	43.2 (41.9–44.5)
Unlikely		1933 (9.8)	6.9 (6.3–7.4)
Extremely unlikely		586 (3.0)	2.8 (2.5–3.1)
Vaccine characteristics influencing vaccination acceptance			
Required doses of COVID-19 vaccine			
Only accept single dose		8025 (40.7)	37.6 (36.4–38.8)
Do not mind		11,689 (59.3)	62.4 (61.2–63.6)
Effectiveness threshold of COVID-19 vaccine			
Only accept 90% threshold		12,625 (64.0)	59.7 (58.4–61.0)
Do not mind		7089 (36.0)	40.3 (39.0–41.6)
Adverse reactions of COVID-19 vaccine			
Only accept minor adverse reactions		14,002 (71.0)	74.5 (73.4–75.5)
Do not mind moderate adverse reactions		5712 (29.0)	25.5 (24.5–26.6)
Duration of COVID-19 vaccine protection			
Only accept no lesser than 12 months		11,452 (58.1)	53.3 (52.0–54.6)
Do not mind		8262 (41.9)	46.7 (45.4–48.0)
Technology used in COVID-19 vaccine			
Do not accept mRNA technology		4030 (20.4)	21.0 (20.0–22.0)
Do not mind		6144 (31.2)	34.0 (32.7–35.2)
Do not know much about mRNA technology		9540 (48.4)	45.1 (43.8–46.3)
Producing country of COVID-19 vaccine			
Only accept a vaccine that is produced by specific countries		11,919 (60.5)	51.8 (50.5–53.1)
Producing countries of a COVID-19 vaccine is not of my concern in vaccine acceptance		7795 (39.5)	48.2 (46.9–49.5)
First foremost important vaccine characteristics influencing COVID-19 vaccine choice			
Effectiveness threshold		7719 (39.2)	35.1 (30.1–40.5)
Adverse reactions		6387 (32.4)	40.6 (33.2–48.4)
Duration of protection		1748 (8.9)	8.7 (7.5–9.9)
Administration doses		1423 (7.2)	7.2 (5.1–10.2)
Country of origin		907 (4.6)	3.4 (1.9–5.8)
Vaccination cost		861 (4.4)	2.5 (1.0–6.1)
mRNA technology		639 (3.2)	2.5 (1.7–3.7)
Second important vaccine characteristics influencing COVID-19 vaccine choice			
Adverse reactions		5140 (26.1)	24.2 (17.8–31.9)
Duration of protection		4630 (23.5)	29.0 (24.8–33.5)
Effectiveness threshold		3729 (18.9)	22.5 (18.3–27.4)
Country or origin		2907 (14.7)	9.3 (4.5–17.9)
Cost of vaccination		1428 (7.2)	4.1 (1.7–9.6)
Administration doses		1230 (6.2)	7.1 (6.0–8.3)
mRNA technology		589 (3.0)	3.9 (3.3–4.6)

^a^African: South Africa; Eastern Mediterranean: Iran, Pakistan, Somalia, United Arab Emirates; European: Norway, United Kingdom; Region of the Americas: United States of America; South-East Asia: Bangladesh, India, Sri Lanka; Western Pacific: Australia. China, Japan, Malaysia, Singapore, Vietnam

### COVID-19 vaccination intention

**Fig. 1:**
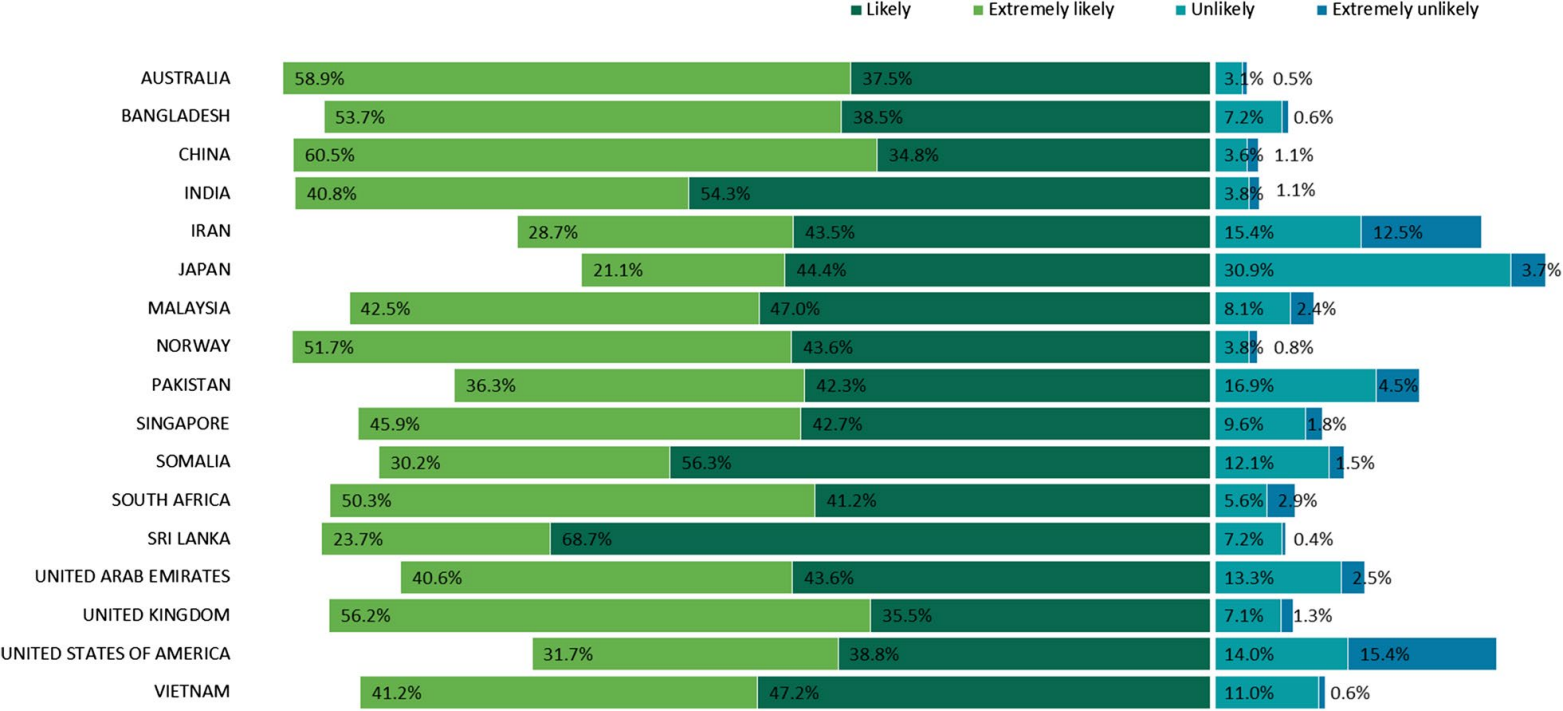
COVID-19 vaccine acceptance by country. *COVID-19* Coronavirus disease 2019; *WHO* World Health Organisation

**Fig. 2:**
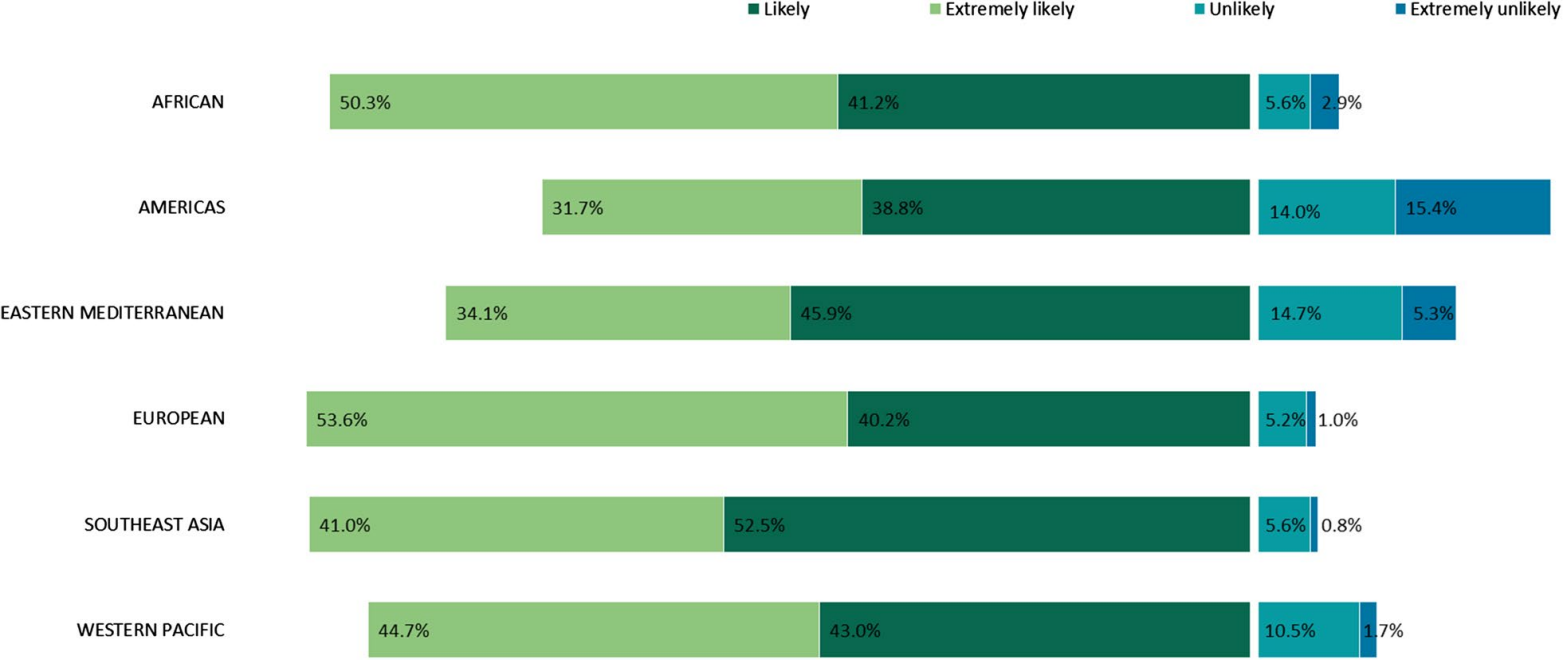
COVID-19 vaccine acceptance by WHO region. *COVID-19* Coronavirus disease 2019, *WHO* World Health Organisation

The majority of the study participants reported that they were *likely* (43.2%) and *extremely likely* (47.2%) to get vaccinated against COVID-19 (Table 1). Figure 1 shows the COVID-19 vaccination intention in the 17 countries. A high proportion of *likely* or *extremely likely* to receive the COVID-19 vaccine was reported in Australia (96.4%), China (95.3%) and Norway (95.3%), while a high proportion reported being *unlikely* or *extremely unlikely* to receive the COVID-19 vaccine in Japan (34.6%), the U.S. (29.4%) and Iran (27.9%). The highest proportion stating that they were *extremely unlikely* to receive the COVID-19 vaccine was recorded in the U.S. (15.4%). Figure 2 shows the distribution of COVID-19 vaccination intentions by WHO region. Southeast Asia and European regions reported high COVID-19 acceptance, whereas lower acceptance was reported in the Americas and Eastern Mediterranean regions.

Table 2 shows the multivariable logistic findings of factors influencing COVID-19 vaccine hesitancy for overall participants. Participants who ever delay or refuse vaccination (_weighted_*OR* = 3.14; 95% *CI* 2.65–3.72), and those with the highest educational level of secondary school or below (*OR* = 1.91; 95% *CI* 1.51–2.42) presented higher odds of vaccine hesitancy. There was a gradual increase in the odds of COVID-19 vaccine hesitancy with age. Female reported lower vaccine hesitancy than males (_weighted_*OR* = 0.84; 95% *CI* 0.72–0.98).

**Table 2:** Demographic characteristics influencing COVID-19 vaccination hesitancy

	Participants (*n* = 19 714)	Extremely unlikely/unlikely vs extremely likely/likely to accept COVID-19 vaccination (*n* = 2518)	
		Unweighted *OR* (95% *CI*)	Weighted *OR* (95% *CI*)
Socio demography			
Age group, years			
18–29	5233 (26.5)	1 (reference)	1 (reference)
30–39	5524 (28.0)	1.22 (1.08–1.38)***	1.43 (1.14–1.79)**
40–49	4070 (20.6)	1.31 (1.14–1.49)***	1.89 (1.48–2.4)***
50–59	2751 (14.0)	1.61 (1.40–1.86)**	1.86 (1.445–2.40)***
60 and above	2136 (10.8)	2.30 (2.00–2.66)***	3.64 (2.84–4.65)***
Gender			
Male	9145 (46.4)	1 (reference)	1 (reference)
Female	10,557 (53.6)	1.03 (0.95–1.13)	0.84 (0.72–0.98)*
Other	12 (0.1)	–	–
Highest education level			
Secondary school and below	2630 (13.3)	2.19 (1.91–2.51)***	1.91 (1.51–2.42)***
Certificate/A-Level/Diploma	4856 (24.6)	1.04 (0.91–1.18)	0.91 (0.72–1.16)
Bachelor degree	7883 (40.0)	1.09 (0.97–1.23)	1.02 (0.83–1.27)
Postgraduate degree	4345 (22.0)	1 (reference)	1 (reference)
Ever delayed acceptance or refuse vaccine despite availability of vaccine service			
Yes	3812 (19.3)	2.60 (2.37–2.85)***	3.14(2.65–3.72)***
No	15,902 (80.7)	1 (reference)	1(reference)

Hosmer–Lemeshow goodness-of-fit Chi-square = 16.834, *P-*value = 0.032; Unweighted Nagelkerke *R*^2^ = 0.075; Weighted Nagelkerke *R*^2^ = 0.091

*P < 0.05, **P < <0.01, ***P < <0.001

By WHO regional comparison (Additional file 5), higher vaccine hesitancy was reported with increasing age, except in the African region. Males expressed higher COVID-19 vaccine hesitancy in the region of the Americas (*OR* = 1.67; 95% *CI* 1.23–2.28) and Southeast Asia (*OR* = 1.55; 95% *CI* 1.16–2.07). In contrast, females expressed higher COVID-19 vaccine hesitancy (*OR* = 1.30; 95% *CI* 1.13–1.50) than males in the Western Pacific region. The multivariable logistic findings of factors influencing COVID-19 vaccine hesitancy for all 17 countries are shown in Additional file 6.

### Vaccine characteristics influencing vaccination acceptance

Findings on attitudes towards the vaccine characteristics revealed that 62.4% (95% *CI* 61.2–63.6) do not mind if the COVID-19 vaccination needs more than one dose (Table 1). A total of 59.7% (95% *CI* 58.4–61.0) reported only accepting a vaccine with more than 90% effectiveness and 74.5% (95% *CI* 73.4–75.5) would accept a COVID-19 vaccine with minor adverse reactions. Slightly over half (53.3%; 95% *CI* 52.0–54.6) reported only accepting a COVID-19 vaccine with a duration of protection of no less than 12 months. The majority of participants do not know about mRNA vaccines (45.1%; 95% *CI* 43.8–46.3) and 21.0% (95% *CI* 20.0–22.0) reported not accepting an mRNA vaccine. Slightly over half (51.8%; 95% *CI* 50.5–53.1) reported that they would only accept a COVID-19 vaccine from a specific country‐of‐origin. Table 3 shows the vaccine characteristics influencing vaccination acceptance by demographics of all participants. Of particular note, participants with the highest level of education of secondary school and below were more likely to accept only single-dose vaccine, an effectiveness threshold no less than 90%, and a vaccine with only minor adverse reactions. Participants of youngest age group (18–29 years) are more likely to not accept mRNA vaccines than the older age groups.

**Table 3:** Vaccine characteristics influencing vaccination acceptance by demographics (*N* = 19,702)

	Required doses of COVID-19 vaccine^a^	Effectiveness threshold of COVID-19 vaccine^b^	Adverse reactions of COVID-19 vaccine^c^	Duration of COVID-19 vaccine protection^d^	Technology used in COVID-19 vaccine^e^	Producing country of COVID-19 vaccine^f^
	Weighted *OR* (95% *CI*)	Weighted *OR* (95% *CI*)	Weighted *OR* (95% *CI*)	Weighted *OR* (95% *CI*)	Weighted *OR* (95% *CI*)	Weighted *OR* (95% *CI*)
Socio demography						
Age group, years						
18–29	1 (reference)	1 (reference)	1 (reference)	1 (reference)	1 (reference)	1 (reference)
30–39	1.63 (1.41–1.87)***	0.72 (0.63–0.83)***	0.58 (0.49–0.68)***	1.01 (0.88–1.16)	1.60 (1.03–2.47)	0.99 (0.85–1.14)
40–49	1.89 (1.61–2.22)***	0.53 (0.45–0.62)***	0.45 (0.38–0.54)***	0.99 (0.85–1.16)	2.29 (1.4–3.75)***	0.91 (0.78–1.06)
50–59	1.91 (1.59–2.28)***	0.61 (0.51–0.73)***	0.41 (0.34–0.51)***	0.98 (0.82–1.17)	2.55 (1.52–4.29)***	0.81 (0.75–1.06)
60 and above	1.14 (0.93–1.387)	0.69 (0.56–0.84)***	0.41 (0.33–0.51)***	1.01 (0.84–1.22)	1.26 (0.71–2.26)*	0.84 (0.69–1.02)
Gender						
Male	1 (reference)	1 (reference)	1 (reference)	1 (reference)	1 (reference)	1 (reference)
Female	0.86 (0.78–0.96)**	1.12 (1.01–1.25)*	1.09 (0.97–1.23)	1.07 (0.96–1.19)	1.31 (1.08–1.57)***	1.08 (0.97–1.21)
Highest education level						
Secondary school and below	1.54 (1.27–1.85)**	1.37 (1.12–1.68)***	1.04 (0.83–1.31)	1.33 (1.10–1.61)***	0.79 (0.45–1.38)	1.08 (0.90–1.30)
Certificate/A-Level/Diploma	1.42 (1.21–1.66)***	0.78 (0.66–0.91)***	0.58 (0.49–0.69)***	0.91 (0.77–1.06)	1.00 (0.64–1.59)	1.04 (0.89–1.21)
Bachelor degree	1.20 (1.04–1.38)***	0.99 (0.86–1.15)	0.86 (0.73–1.01)	1.04 (0.91–1.20)	0.78 (0.49–1.25)	0.84 (0.73–0.97)*
Postgraduate degree	1 (reference)	1 (reference)	1 (reference)	1 (reference)	1 (reference)	1 (reference)
Ever delayed acceptance or refuse vaccine despite availability of vaccine service						
Yes	1.71 (1.49–1.96)***	1.77 (1.52–2.07)***	1.82 (1.52–2.16)***	1.52 (1.32–1.75)***	1.284(0.783–2.107)	0.63 (0.55–0.73)***
No	1 (reference)	1 (reference)	1 (reference)	1 (reference)	1 (reference)	1 (reference)

Other gender was excluded due to small sample size

a: Only accept single dose vs Do not mind, b: Only accept 90% threshold vs Do not mind, c: Only accept minor adverse reactions vs Do not mind moderate adverse reactions, d: Only accept lesser than 12 months vs Do not mind moderate adverse reactions, e: Do not accept mRNA technology vs Do not know much about mRNA technology/Do not mind, f: Only accept a vaccine that is produced by specific countries vs Producing countries of a COVID-19 vaccine is not of my concern in vaccine choice

^a^*P*-value: *P* < 0.001; Nagelkerke *R*^2^: 0.044

^b^*P*-value: *P* < 0.001; Nagelkerke *R*^2^: 0.044

^c^*P*-value: 0.001; Nagelkerke *R*^2^: 0.066

^d^*P*-value: 0.001; Nagelkerke *R*^2^: 0.013

^e^*P*-value: 0.001; Nagelkerke *R*^2^: 0.013

^f^*P*-value: 0.010; Nagelkerke *R*^2^: 0.014

*P < 0.05, **P < <0.01, ***P < <0.001

**Fig. 3:**
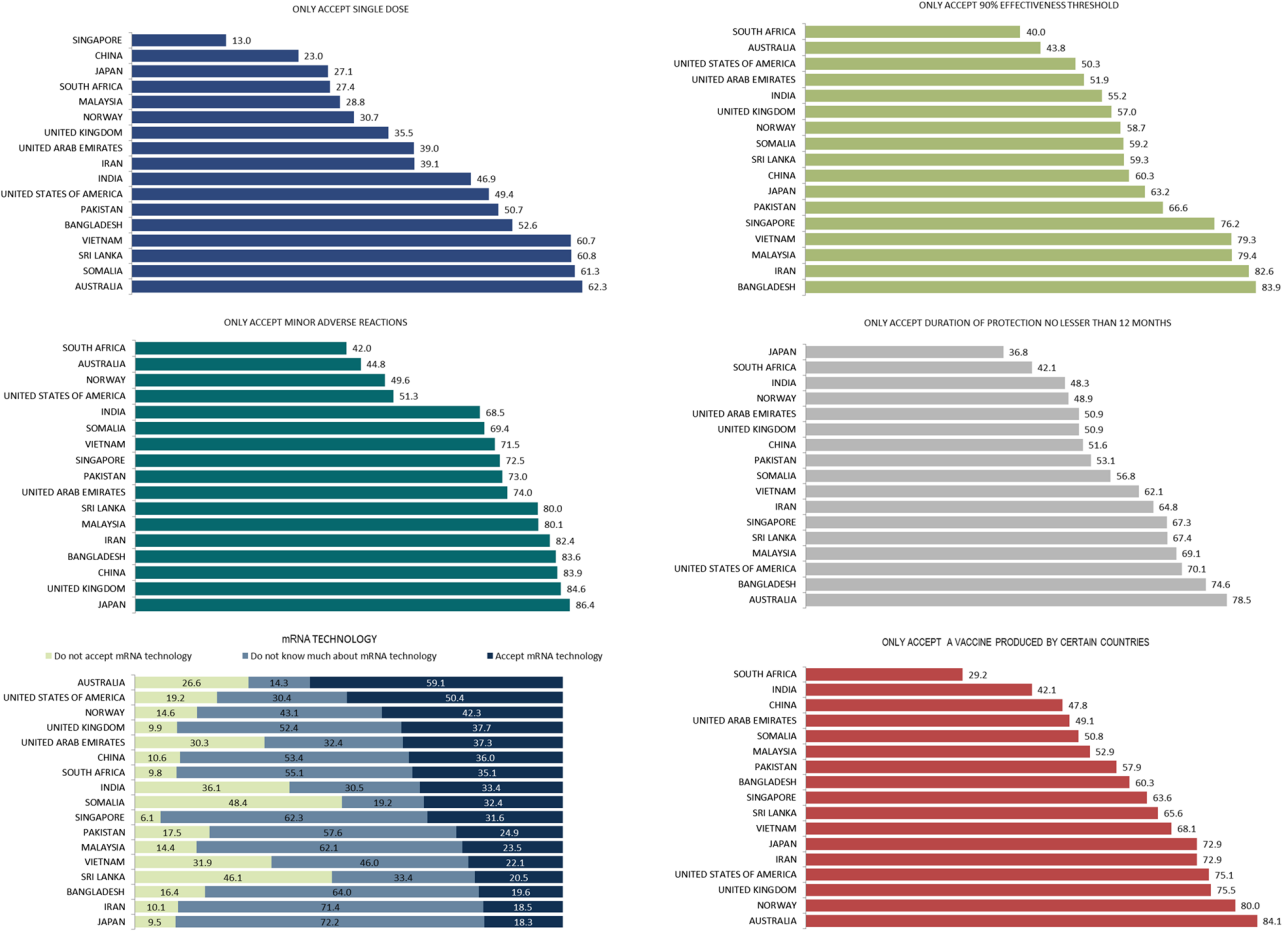
COVID-19 vaccine characteristics preferences by country. *COVID-19* Coronavirus disease 2019, *WHO* World Health Organisation

**Fig. 4:**
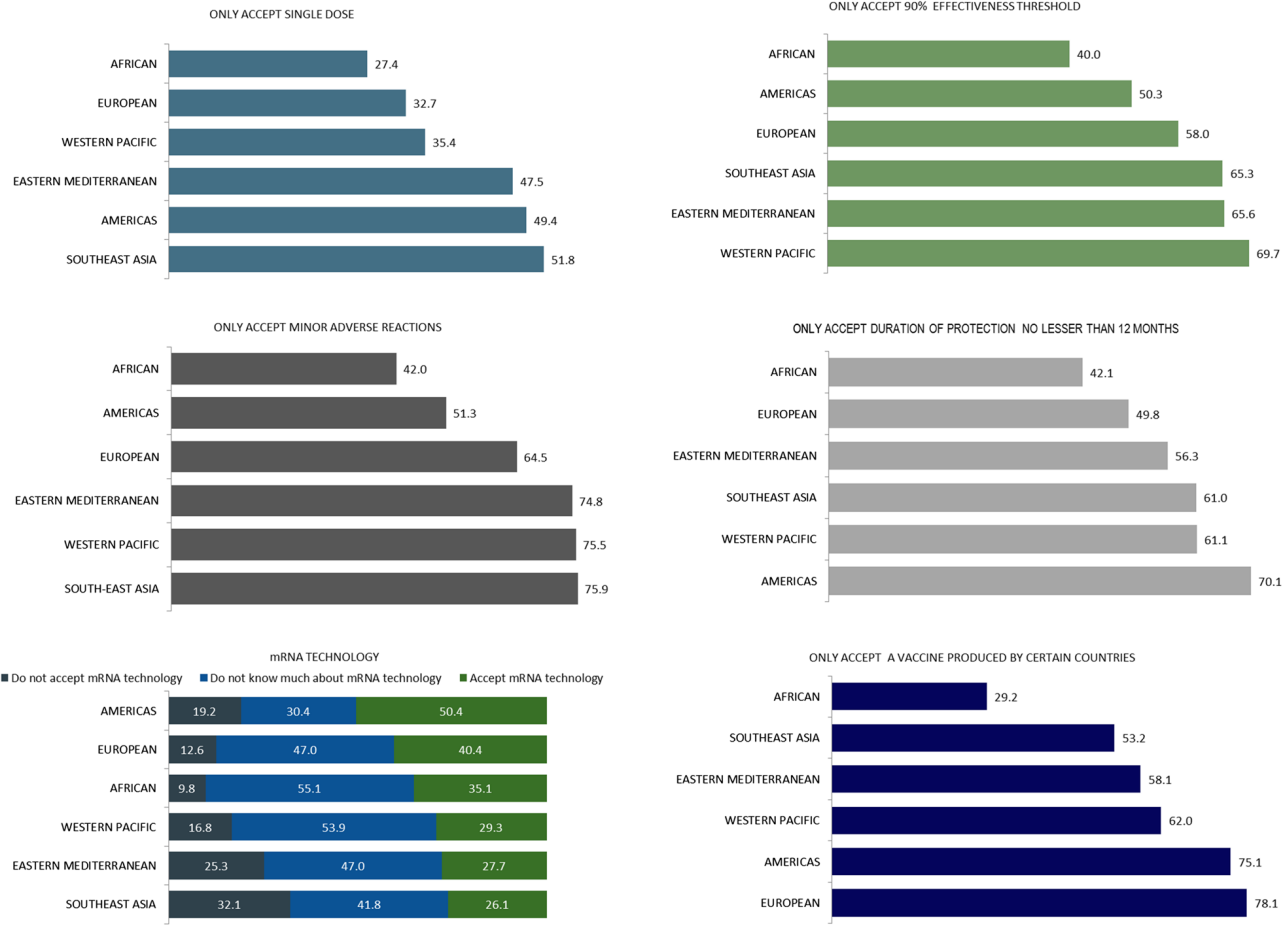
COVID-19 vaccine characteristics preferences by WHO region. *COVID-19* Coronavirus disease 2019, *WHO* World Health Organisation

The distribution of attitudes about the vaccine characteristics by individual countries is shown in Fig. 3. Australia ranked highest in perceived acceptance of a single-dose COVID-19 vaccine, a duration of protection of not less than 12 months and only accepting a vaccine produced by certain countries. Somalia (48.4%) and Sri Lanka (46.1%) recorded the highest proportion that would not accept an mRNA vaccine. Japan (72.2%) and Iran (71.4%) recorded a higher proportion that do not know about mRNA vaccines. Figure 4 shows the distribution of attitudes about the vaccine characteristics by WHO region. Countries from the Southeast Asia region reported the highest proportion not accepting mRNA technology, and only accepting minor adverse reactions and a single-dose vaccine. The highest proportion of the European and Americas regions reported only accepting a vaccine produced by certain countries. The multivariable logistic findings of demographic factors influencing COVID-19 vaccine characteristic acceptance for the 17 individual countries are shown in Additional file 7

### Desirable vaccine characteristics influencing vaccine choice

The foremost important vaccine characteristic influencing vaccine choice is adverse reactionss (40.6%; 95% *CI* 39.3–41.9) of a vaccine and effectiveness threshold (35.1%; 95% *CI* 33.9–36.4). The second most important factors were the duration of protection (29.0%; 95% *CI* 27.8–30.2) and adverse reactions of a vaccine (24.2%; 95% *CI* 23.0–25.4). The first and second most important vaccine characteristics influencing vaccine choice of the 17 individual countries are shown in Additional file 4

## Discussion

The survey assessed COVID-19 vaccination acceptance among 19,714 respondents from 17 countries across all WHO regions as soon as the United States Food and Drug Administration (FDA) authorised vaccines and their roll-out started around the world. The finding of 90.4% reporting being *likely* or *extremely likely* to accept vaccination implies a high level of COVID-19 vaccine intention. Vaccine intention varies from the highest of 96.4% (Australia) to the lowest of 65.5% (Japan). By region, the countries in Southeast Asia reported the highest acceptance and the Americas reported the lowest. As with a previous global study of COVID-19 vaccination intention [19], acceptance tended to be high in the Asian nations, where the public has strong institutional trust. A notable exception is Japan, a country known to have one of the lowest rates of vaccine confidence worldwide [20] and this was similar to the results found for COVID-19 vaccine intent in our study. A recent study of COVID-19 vaccination intention among Japanese people similarly found a vaccination intention of only 65.7% [21]. Our findings echo the relatively low intentions for COVID-19 vaccination among people in the U.S. [22–25]. Based on estimates that vaccination coverage of approximately 75% may be required to control the current epidemic [26], the current findings suggest that Iran, the U.S. and Japan (the countries with vaccination intention below the threshold) would warrant concerted efforts to improve acceptability and uptake in their populations.

In this study, hesitancy was almost two-fold higher among people aged 60 years and older than other younger age groups. Reports of deaths occurring in the elderly who received a COVID-19 vaccine made headlines worldwide [27, 28], perhaps raising some concern about the vaccines that are too risky for the elderly, resulting in an increase in hesitancy among the elderly. As COVID-19 vaccination is underway in many countries, and people aged 65 and older are the initial priority group for a COVID-19 vaccination program in many countries, providing information and support to older people is important to enhance vaccination coverage in older adults. On the whole, the prevalence of COVID-19 vaccine hesitancy remains disproportionately high in individuals who have an education level of secondary level and below, which is a consistent finding across regions and many individual countries. Higher level of COVID-19 vaccine hesitancy in people with lower education levels found in this study can be explained by pre-existing vaccine hesitancy in these groups, namely due to lower knowledge about vaccines and health literacy; in addition, lower trust in healthcare professionals, the health system and the government [29, 30]. Research shows that better educated individuals are more likely to understand public health messages and access reliable information on the safety and effectiveness of vaccines [31]. The findings bring to light the importance of developing targeted interventions within each country, directed at subgroups who are hesitant, to increase vaccination confidence and coverage.

This study shows that vaccine characteristics have an important influence on the participants’ vaccination acceptance. As the highest proportion reported only accepting a COVID-19 vaccine with minor adverse reactions, this indicates that the safety of a new COVID-19 vaccine is an extremely important characteristic for vaccine acceptance. It is possible that people worldwide are worried about the safety of vaccines because of their novelty and the fact that the COVID-19 vaccine is also the first in history being approved for emergency use and rolled out on a global scale. The public should be made known that despite being rolled out for emergency use, the COVID-19 vaccines have gone through rigorous, multi-stage testing processes, including large clinical trials, and were found to be safe and effective [32]. The recent evidence of reductions of SARS-CoV-2 infections, hospitalisations and deaths following nationwide COVID-19 vaccinations should also be informed to the public [33, 34].

Of notable importance, this study found a high proportion indicating that they would only accept vaccines with a threshold of above 90% effectiveness. There has been a widespread comparison of the efficacy rates of the COVID-19 vaccines in the news media [35, 36]. This might lead people to be more unwilling to accept vaccines with a lower level of effectiveness, having the impression that a lower level of effectiveness means that they are inferior. Nevertheless, the public should be informed that the effectiveness of these vaccines has not been compared directly, so comparative effectiveness remains largely unknown. Also, the most widely reported efficacy data is based on an endpoint of symptomatic disease, whereas there may be less variability across vaccines when considering severe disease or hospitalization as the endpoint. Given the uncertainty of the comparison of the effectiveness of the currently available COVID-19 vaccines, the public should be informed that with high immunisation rates, a vaccine with an effectiveness of just 60% or 70% may be sufficient to reach herd immunity and potentially control the pandemic [26]. Hence, it is essential to educate members about herd immunity and the importance of concerted efforts to ensure successful vaccination of a large proportion of the population to achieve high immunisation coverage rates.

In this study, a substantially high proportion of people in the Europe and Americas regions reported not accepting a COVID-19 vaccine produced by specific countries. In contrast, Southeast Asia and African regions expressed less concern surrounding the country-of-origin of the COVID-19 vaccine. The disparities in COVID-19 vaccine acceptance found in this study has tremendously important implications for respective governments in the choice of vaccine to be introduced in their countries’ vaccination implementation program. The general public should be made aware that all three of the COVID-19 vaccines currently authorised by the FDA as well as other COVID-19 vaccines that have received regulatory approval from the countries’ origin regulatory approval have been proven to be safe and effective for their intended use. There is a need to increase the public’s faith in any approved vaccine offered to them. Given the urgency in vaccine deployment and reaching high coverage, the public should be encouraged to accept the COVID-19 vaccines offered to them.

Our findings also indicate vaccine hesitancy increasing due to the uncertainty surrounding the duration of protection and the number of doses, making these of considerable concern. Unfortunately, the questions surrounding the duration of vaccine-elicited protection and the need for booster injections are currently the focus of ongoing investigations and it is unknown whether booster doses will be needed. Currently, 6 months after the authorization of the COVID-19 vaccines, real-world data from several countries has continued to demonstrate strong protection against SARS-CoV-2 infections through 6 months post-second dose [37, 38]. Despite preceding speculations that the mutations of SARS-CoV-2 would adversely affect the efficacy of the COVID-19 vaccines, to date, mounting evidence showed that the COVID-19 vaccines coffer protection against the current prevailing variants of the SARS-CoV-2. Earlier in the pandemic, evidence indicated that vaccines are unlikely to be affected by the ‘D614G’ mutation (aspartate-to-glycine change at position 614) of the SARS-CoV-2 spike protein [39]. Preliminary laboratory studies on the mRNA vaccine reported that it offers protection from multiple variants including the B.1.351 variant first found in South Africa, the B.1.1.7 variant first found in the United Kingdom, and the P.1 variant first found in Brazil [40, 41]. A recent serosurvey study showed that BNT162b2 vaccine-elicited antibodies efficiently neutralize SARS-CoV-2 authentic viruses belonging to B.1, B.1.1.7, B.1.351, B.1.525 and P.1 lineages [42]. It remains a challenge to convince the public to accept a new vaccine with unknown duration of protection. Nonetheless, highlighting these recent evidence-based facts will be useful to counteract the fear of being vaccinated with a vaccine that may no longer provide protection against COVID-19.

Our study found that a minority, particularly people from the Southeast Asia region, were concerned about getting vaccines developed using mRNA technology. The ground-breaking approach previously had not been tested in humans, causing concerns about possible safety issues [43]. It remains a challenge in introducing the new mRNA vaccines and clearly communicating that they have been adequately evaluated for safety and efficacy in clinical trials, despite the fact that they involve relatively new biotechnology [44, 45]. The mRNA vaccines have been subjected to many conspiracy theories since they were launched [46]. The benefits of the mRNA vaccine along with the current safety evidence [44, 47–49] should be highlighted to demystify the unfounded conspiracy theories and criticism.

Currently, in many countries, the public may not have the option to choose the type of COVID-19 vaccine that they favour; however, the responses to factors influencing the choice of vaccine imply that, above all, the safety and efficacy of the new vaccines are of paramount importance to the world population. Thus, efforts need to be made to build trust in the safety and efficacy of the vaccines offered to the general population within all countries. Since there are still many uncertainties surrounding the risks of the new COVID-19 vaccines, media reports of COVID-19 vaccines causing serious or lethal side effects might cause public uproar and fear, resulting in people refusing vaccination. There is a strong need to educate the public and media outlets that anecdotal evidence is not a valid way to determine safety or efficacy.

A large population-based SARS-CoV-2 seroepidemiological study in Germany reported that about 20% of individuals lost their neutralizing antibodies within 5 months post infection and neutralizing antibodies are detectable in only one-third of individuals who were tested positive [50]. A recent finding of the first long-term seroprevalence study in Wuhan, China after the lockdown lifted revealed that only around 7% of the population had COVID-19 antibodies, with approximately 40% of this population developing neutralising antibodies that potentially protect against future infection [51]. This evidence supports the need for mass vaccination to reach herd immunity and should be highlighted to all communities globally to prevent further resurgences of the pandemic. A whole-of-society approach is needed to achieve a successful global COVID-19 vaccine program.

The main limitation of this study was the use of the convenience sampling method to administer the questionnaire, which may have introduced selection bias and affected generalisation to the wider populations. Secondly, we recognise that this study has a strong representation of countries in the Southeast Asia, Eastern Mediterranean, and Western Pacific regions, but limited representation in the Americas, Africa, and European regions. Also noteworthy for the present study, the native language option of the survey questions was available for the survey conducted in China, Vietnam, Sri Lanka, the United Arab Emirates, Malaysia, and Japan, which may introduce a biased representation of English-speaking participants for countries where native language option was not available.

## Conclusions

The refusal of COVID-19 vaccination could prolong the battle against this pandemic and result in needless suffering and death. The findings provide the demographic targets of the people who most need to be reached for respective countries and regions to increase vaccine acceptance and uptake rates. It is clear that the different types of COVID-19 vaccines with diverse characteristics that are currently available may increase uncertainty and difficulty in making a decision, resulting in people delaying or refusing vaccination. Furthermore, not being able to have the option to freely choose a favoured vaccine may heighten hesitancy. Given the importance of moving quickly to roll out the vaccine to reach a herd immunity threshold, and the current situation of insufficient supply to meet the current global demand, addressing this reluctance through evidence gained from this study would be advantageous. It is of paramount importance that all countries develop individually-tailored strategies to strengthen the confidence of their population in vaccination, irrespective of the types of COVID-19 vaccines offered in their national vaccination program. Our findings may also provide insights enabling public health messages to be tailored to enhance COVID-19 vaccine uptake efforts worldwide.

## Supplementary Information

**Additional file 1.** Questionnaire.

**Additional file 2. **The population size of age 15 years and the sample population by country.

**Additional file 3. **Participant demographics, vaccine characteristics influencing vaccination acceptance and vaccine characteristics influencing choice by region.

**Additional file 4. **Participant demographics, vaccine characteristics influencing vaccination acceptance and vaccine characteristics influencing choice by country.

**Additional file 5. **Factors influencing COVID-19 vaccine hesitancy by region.

**Additional file 6. **Factors influencing COVID-19 vaccine hesitancy by country.

**Additional file 7.** Vaccine characteristics influencing vaccination acceptance by demographics for individual country.

## Data Availability

All data generated or analysed during this study are included in this published article [and its additional information files].

## Abbreviations

*CI*: Confidence interval
COVID-19: Coronavirus disease 2019
FDA: Food and Drug Administration
mRNA: Messenger RNA
*OR*: Odds ratio
SARS-CoV-2: Severe acute respiratory syndrome coronavirus 2
USA: United States of America
WHO: World Health Organisation
