# Supplementary material for: COVID-19 vaccination intention and vaccine characteristics influencing vaccination acceptance: a global survey of 17 countries
Source: Infect Dis Poverty. 2021 Oct 7;10:122. doi: 10.1186/s40249-021-00900-w (PMC8496428; doi:10.1186/s40249-021-00900-w)
Supplement: Supplementary file 1 — Additional file 1. Questionnaire. [file 40249_2021_900_MOESM1_ESM.docx]

**Acceptance and Preference of COVID-19 vaccine:**

**A global survey**

**Section A: Demographics**

| A1 | What is your age group? | | | | | | | | | | | | |
| --- | --- | --- | --- | --- | --- | --- | --- | --- | --- | --- | --- | --- | --- |
|  | [ ] 18-29 | [ ] 30-39 | | [ ] 40-49 | | [ ] 50-59 | | [ ] 60-69 | [ ] 70-79 | [ ] 80-89 | [ ] 90-99 | | [ ]  100 and above |
| A2 | What is your gender? | | | | | | | |  | | | | |
|  | [ ] Male | | | | [ ] Female | | | | [ ] Others | | | | |
| A3 | What is your highest educational attainment? | | | | | | | |  | | | | |
|  | [ ] Primary school | | [ ] Secondary school/  O-Level | | | | [ ] Certificate/  College/  A-Level/  Diploma | | [ ]  Bachelor degree | | | [ ]  Postgraduate degree | |
| A4 | Have you ever delay acceptance or refuse vaccines despite the availability of vaccine service? | | | | | | | | | | | | |
|  | [ ] Yes | | | | [ ] No | | | |  | | | | |

**Section B: COVID-19 vaccine acceptance**

| B1 | Would you accept the COVID-19 vaccine if it is recommended by the government in your country and the COVID-19 vaccination service is available? | | | |
| --- | --- | --- | --- | --- |
|  | [ ]  Extremely likely | [ ]  Likely | [ ]  Unlikely | [ ]  Extremely unlikely |

**Section C: Vaccine characteristics influencing vaccination acceptance**

| C1 | Required doses of COVID-19 vaccine | |  | |
| --- | --- | --- | --- | --- |
|  | [ ]  I **WILL ONLY ACCEPT** the vaccine if just a **single vaccine dose** is required | | [ ]  I **DO NOT MIND** if a **booster dose is required** following a primary vaccination | |
| C2 | Effectiveness threshold of COVID-19 vaccine | | | |
|  | [ ]  I **WILL ONLY ACCEPT** a vaccine that reported **nearly 90% effective or above** in preventingCOVID-19 | | [ ]  I **DO NOT MIND** receiving a vaccine that reported **below 90% effective** in preventingCOVID-19 | |
| C3 | Side-effects of COVID-19 vaccine | |  | |
|  | [ ]  I **WILL ONLY ACCEPT** a vaccine that has **minor side effects** such as soreness, swelling at the injection site that **does not disrupt daily life** | | [ ]  I **DO NOT MIND** if the vaccine has **moderate side effects** such as fever, headaches that **disrupt daily life** | |
| C4 | Duration of protection of COVID-19 vaccine | | | |
|  | [ ]  I **WILL ONLY ACCEPT** a vaccine with duration protection **no shorter than 12 months** | | [ ]  I **DO NOT MIND** if the duration of protection of vaccine **between 6 and 12 months** | |
| C5 | Technology used in COVID-19 vaccine production | | | |
|  | [ ]  I **WILL NOT ACCEPT** a COVID-19 vaccine developed using mRNA technology | [ ]  I **DO NOT MIND** receiving an mRNA COVID-19 vaccine | | [ ]  I **DO NOT KNOW MUCH** about mRNA technology |
| C6 | Producing country of COVID-19 vaccine | | | |
|  | [ ]  I **WILL ONLY ACCEPT** a vaccine that is produced by **specific countries** | | [ ]  The producing countries of a COVID-19 vaccine are **NOT OF MY CONCERN** in vaccine acceptance | |

**Section D: Factors influencing COVID-19 vaccine choice**

| D1 | Which of the following is the ***FIRST*** foremost important factor influencing your choice of COVID-19 vaccine? | | | | | | | | | | | |
| --- | --- | --- | --- | --- | --- | --- | --- | --- | --- | --- | --- | --- |
|  | [ ] Number of doses | [ ] Effectiveness threshold | | | [ ] Side effects | [ ] Duration of protection | [ ] mRNA technology | | [ ] Producing countries | | [ ]  Total cost of vaccination (including booster if required) | |
| D2 | Which of the following is the ***SECOND*** foremost important factor influencing your choice of COVID-19 vaccine? | | | | | | | | | | | |
|  | [ ] Number of doses | | [ ] Effectiveness threshold | [ ] Side effects | | [ ] Duration of protection | | [ ] mRNA technology | | [ ] Producing countries | | [ ]  Total cost of vaccination (including booster if required) |
