# Supplementary material for: COVID-19 vaccination intention and vaccine characteristics influencing vaccination acceptance: a global survey of 17 countries
Source: Infect Dis Poverty. 2021 Oct 7;10:122. doi: 10.1186/s40249-021-00900-w (PMC8496428; doi:10.1186/s40249-021-00900-w)
Supplement: Supplementary file 2 — Additional file 2. The population size of age 15 years and the sample population by country. [file 40249_2021_900_MOESM2_ESM.docx]

The population size of age 15 years and the sample population by country

|  | Country population (aged 15 and above)⁺ | Sample population | % |
| --- | --- | --- | --- |
| **African region** |  |  |  |
| South Africa | 42,221,600 | 1086 | 5.5 |
| **Eastern Mediterranean region** |  |  |  |
| Iran | 63,252,000 | 1019 | 5.2 |
| Pakistan | 143,805,900 | 1271 | 6.4 |
| Somalia | 8,554,200 | 894 | 4.5 |
| United Arab Emirates | 8,434,800 | 938 | 4.8 |
| **European region** |  |  |  |
| Norway | 4,465,800 | 1382 | 7.0 |
| United Kingdom | 55949600 | 1021 | 5.2 |
| **Region of the Americas** |  |  |  |
| United States of America | 270,096,000 | 968 | 4.9 |
| **Southeast Asia** |  |  |  |
| Bangladesh | 120,560,400 | 1094 | 5.5 |
| India | 1,019,820,000 | 1566 | 7.9 |
| Sri Lanka | 16,328,200 | 776 | 3.9 |
| **Western Pacific region** |  |  |  |
| Australia | 20,578,500 | 811 | 4.1 |
| China | 1,184,543,900 | 1373 | 7.0 |
| Japan | 110,814,000 | 1037 | 5.3 |
| Malaysia | 24,818,400 | 2175 | 11.0 |
| Singapore | 5,174,300 | 841 | 4.3 |
| Vietnam | 74,726,400 | 1462 | 7.4 |

⁺The population size data was obtained from the United Nations Population Fund (2021). World Population Dashboard. <https://www.unfpa.org/data/world-population-dashboard>.

Note: Population size of age 15 years and above was used as the population size of age 18 years and above was not available.
