# Supplementary material for: COVID-19 vaccination intention and vaccine characteristics influencing vaccination acceptance: a global survey of 17 countries
Source: Infect Dis Poverty. 2021 Oct 7;10:122. doi: 10.1186/s40249-021-00900-w (PMC8496428; doi:10.1186/s40249-021-00900-w)
Supplement: Supplementary file 3 — Additional file 3. Participant demographics, vaccine characteristics influencing vaccination acceptance and vaccine characteristics influencing choice by region. [file 40249_2021_900_MOESM3_ESM.docx]

Participant demographics, vaccine characteristics influencing vaccination acceptance and vaccine characteristics influencing choice by region

|  | **African**  **n=1086** | **Eastern Mediterranean**  **n=4122** | **European**  **n=2403** | **Region of the Americas**  **n=968** | **Southeast Asia**  **n=3436** | **Western Pacific**  **n=7699** |
| --- | --- | --- | --- | --- | --- | --- |
| **Demographics** |  |  |  |  |  |  |
| ***Age group*** |  |  |  |  |  |  |
| 18 - 29 | 194 (17.9) | 1105 (26.8) | 547 (22.8) | 158 (16.3) | 1211 (35.2) | 2018 (26.2) |
| 30 - 39 | 267 (24.6) | 1125 (27.3) | 789 (32.8) | 323 (33.4) | 834 (24.3) | 2186 (28.4) |
| 40 - 49 | 254 (23.4) | 867 (21.0) | 477 (19.9) | 250 (25.8) | 656 (19.1) | 1566 (20.3) |
| 50 - 59 | 186 (17.1) | 541 (13.1) | 380 (15.8) | 121 (12.5) | 523 (15.2) | 1000 (13.0) |
| 60 and above | 185 (17.0) | 484 (11.7) | 210 (8.7) | 116 (12.0) | 212 (6.2) | 929 (12.1) |
| ***Gender*** |  |  |  |  |  |  |
| Male | 432 (39.8) | 2041 (49.5) | 1322 (55.0) | 471 (48.7) | 1493 (43.5) | 3386 (44.0) |
| Female | 654 (60.2) | 2081 (50.5) | 1081 (45.0) | 485 (50.1) | 1943 (56.5) | 4313 (56.0) |
| Other |  |  |  | 12 (1.2) |  |  |
| **Highest education level** |  |  |  |  |  |  |
| Secondary school and below | 168 (15.5) | 880 (21.3) | 62 (2.6) | 115 (11.9) | 246 (7.2) | 1159 (15.1) |
| Certificate/A-Level/Diploma | 405 (37.3) | 813 (19.7) | 769 (32.0) | 317 (32.7) | 703 (20.5) | 1849 (24.0) |
| Bachelor degree | 224 (20.6) | 1562 (37.9) | 831 (34.6) | 441 (45.6) | 1425 (41.5) | 3400 (44.2) |
| Postgraduate degree | 289 (26.6) | 867 (21.0) | 741 (30.8) | 95 (9.8) | 1062 (30.9) | 1291 (16.8) |
| **Ever delayed acceptance or refuse vaccine despite availability of vaccine service** |  |  |  |  |  |  |
| Yes | 79 (7.3) | 1833 (44.5) | 228 (9.5) | 140 (14.5) | 568 (16.5) | 964 (12.5) |
| No | 1007 (92.7) | 2289 (55.5) | 2175 (90.5) | 828 (85.5) | 2868 (83.5) | 6735 (87.5) |
| **Vaccine characteristics influencing vaccination acceptance** |  |  |  |  |  |  |
| ***Required doses of COVID-19 vaccine*** |  |  |  |  |  |  |
| Only accept single dose | 298 (27.4) | 1957 (47.5) | 786 (32.7) | 478 (49.4) | 1781 (51.8) | 2725 (35.4) |
| Do not mind | 788 (72.6) | 2165 (52.5) | 1617 (67.3) | 490 (50.6) | 1655 (48.2) | 4974 (64.6) |
| ***Effectiveness threshold of COVID-19 vaccine*** |  |  |  |  |  |  |
| Only accept 90% threshold | 434 (40.0) | 2704 (65.6) | 1393 (58.0) | 487 (50.3) | 2243 (65.3) | 5364 (69.7) |
| Do not mind | 652 (60.0) | 1418 (34.4) | 1010 (42.0) | 481 (49.7) | 1193 (34.7) | 2335 (30.3) |
| ***Adverse reactions of COVID-19 vaccine*** |  |  |  |  |  |  |
| Only accept minor adverse reactions | 456 (42.0) | 3082 (74.8) | 1549 (64.5) | 497 (51.3) | 2609 (75.9) | 5809 (75.5) |
| Do not mind moderate adverse reactions | 630 (58.0) | 1040 (25.2)0 | 854 (35.5) | 471 (48.7) | 827 (24.1) | 1890 (24.5) |
| ***Duration of COVID-19 vaccine protection*** |  |  |  |  |  |  |
| Only accept no lesser than 12 months | 457 (42.1) | 2320 (56.3) | 1196 (49.8) | 679 (70.1) | 2096 (61.0) | 4704 (61.1) |
| Do not mind | 629 (57.9) | 1802 (43.7) | 1207 (50.2) | 289 (29.9) | 1340 (39.0) | 2995 (38.9) |
| ***Technology used in COVID-19 vaccine*** |  |  |  |  |  |  |
| Do not accept mRNA technology | 1086 (9.8) | 1042 (25.30 | 303 (12.6) | 186 (19.2) | 1103 (32.1) | 1290 (16.8) |
| Do not mind | 382 (35.2) | 1144 (27.8) | 970 (40.4) | 488 (50.4) | 897 (26.1) | 2263 (22.94) |
| Do not know much about mRNA technology | 598 (55.1) | 1936 (47.0) | 1130 (47.0) | 294 (30.4) | 1436 (41.8) | 4146 (53.9) |
| ***Producing country of COVID-19 vaccine*** |  |  |  |  |  |  |
| Only accept a vaccine that is produced by specific countries | 317 (29.2) | 2394 (58.1) | 1877 (78.1) | 727 (75.1) | 1829 (53.2) | 4775 (62.0) |
| Producing countries of a COVID-19 vaccine is not of my concern in vaccine acceptance | 769 (70.8) | 1728 (41.9) | 526 (21.9) | 241 (24.9) | 1607 (46.8) | 2924 (38.0) |
| **First foremost important vaccine characteristics influencing COVID-19 vaccine choice** |  |  |  |  |  |  |
| Effectiveness threshold | 441 (40.6) | 976 (23.9) | 1280 (53.3) | 336 (34.7) | 1301 (37.9) | 3385 (44.0) |
| Adverse reactions | 276 (25.4) | 1317 (32.2) | 579 (24.1) | 384 (39.7) | 1145 (33.3) | 2686 (34.9) |
| Duration of protection | 210 (19.3) | 506 (12.4) | 140 (5.8) | 74 (7.6) | 400 (11.6) | 418 (5.8) |
| Administration doses | 77 (7.1) | 413 (10.1) | 192 (8.0) | 55 (5.7) | 324 (9.4) | 362 (4.7) |
| Vaccination cost | 22 (2.0) | 370 (9.0) | 20 (0.8) | 38 (3.9) | 151 (4.4) | 306 (4.0) |
| Country of origin | 26 (2.4) | 271 (6.6) | 63 (2.6) | 38 (3.9) | 55 (1.6) | 408 (5.3) |
| mRNA technology | 34 (3.1) | 239 (5.8) | 129 (5.4) | 43 (4.4) | 60 (1.7) | 134 (1.7) |
| **Second important vaccine characteristics influencing COVID-19 vaccine choice** |  |  |  |  |  |  |
| Adverse reactions | 264 (24.3) | 863 (20.9) | 360 (15.0) | 179 (18.5) | 1109 (32.3) | 2365 (30.7) |
| Duration of protection | 411 (37.8) | 951 (23.1) | 668 (27.8) | 231 (23.9) | 920 (26.8) | 1449 (18.8) |
| Effectiveness threshold | 190 (17.5) | 726 (17.6) | 67 (2.8) | 104 (10.7) | 704 (20.5) | 1938 (28.5) |
| Country of origin | 72 (6.6) | 577 (14.0) | 1039 (43.2) | 258 (26.7) | 101 (2.9) | 386 (5.0) |
| Cost of vaccination | 29 (2.7) | 534 (13.0) | 135 (5.6) | 73 (7.5) | 214 (6.2) | 779 (10.1) |
| Administration doses | 96 (8.8) | 327 (7.9) | 61 (2.5) | 93 (9.6) | 267 (7.8) | 585 (7.6) |
| mRNA technology | 24 (2.2) | 144 (3.5) | 73 (3.0) | 30 (3.1) | 121 (3.5) | 197 (2.6) |
