# Supplementary material for: COVID-19 vaccination intention and vaccine characteristics influencing vaccination acceptance: a global survey of 17 countries
Source: Infect Dis Poverty. 2021 Oct 7;10:122. doi: 10.1186/s40249-021-00900-w (PMC8496428; doi:10.1186/s40249-021-00900-w)
Supplement: Supplementary file 4 — Additional file 4. Participant demographics, vaccine characteristics influencing vaccination acceptance and vaccine characteristics influencing choice by country. [file 40249_2021_900_MOESM4_ESM.docx]

Participant demographics, vaccine characteristics influencing vaccination acceptance and vaccine characteristics influencing choice by country

|  | **Australia**  **n=811** | **Bangladesh**  **n=1094** | **China**  **n=1373** | **India**  **n=1566** | **Iran**  **n=1019** | **Japan**  **n=1037** |
| --- | --- | --- | --- | --- | --- | --- |
| **Demographics** |  |  |  |  |  |  |
| ***Age group*** |  |  |  |  |  |  |
| 18 - 29 | 123 (15.2) | 470 (43.0) | 760 (55.4) | 481 (30.7) | 297 (29.1) | 182 (17.6) |
| 30 - 39 | 265 (32.7) | 395 (36.1) | 357 (26.0) | 324 (20.7) | 287 (28.2) | 200 (19.3) |
| 40 - 49 | 277 (34.2) | 125 (11.4) | 134 (9.8) | 323 (20.6) | 204 (20.0) | 207 (20.0) |
| 50 - 59 | 103 (12.7) | 49 (4.5) | 77 (5.6) | 307 (19.6) | 134 (13.2) | 167 (16.1) |
| 60 and above | 43 (5.3) | 55 (5.0) | 45 (3.3) | 131 (8.4) | 97 (9.5) | 281 (27.1) |
| ***Gender*** |  |  |  |  |  |  |
| Male | 423 (52.2) | 551 (50.4) | 593 (43.2) | 743 (47.4) | 488 (47.9) | 456 (44.0) |
| Female | 388 (47.8) | 543 (49.6) | 780 (56.8) | 823 (52.6) | 531 (52.1) | 581 (56.0) |
| ***Highest education level*** |  |  |  |  |  |  |
| Secondary school and below | 36 (4.4) | 97 (8.9) | 177 (12.9) | 133 (8.5) | 48 (4.7) | 237 (22.9) |
| Certificate/A-Level/Diploma | 276 (34.0) | 79 (7.2) | 243 (17.7) | 422 (26.9) | 205 (20.1) | 199 (19.2) |
| Bachelor degree | 412 (50.8) | 403 (36.8) | 693 (50.5) | 560 (35.8) | 490 (48.1) | 417 (40.2) |
| Postgraduate degree | 87 (10.7) | 515 (47.1) | 260 (18.9) | 451 (28.8) | 276 (27.1) | 184 (17.7) |
| ***Ever delayed acceptance or refuse vaccine despite availability of vaccine service*** |  |  |  |  |  |  |
| Yes | 90 (11.1) | 356 (32.5) | 302 (22.0) | 150 (9.6) | 715 (70.2) | 161 (15.5) |
| No | 721 (88.9) | 738 (67.5) | 1071 (78.0) | 1416 (90.4) | 304 (29.8) | 876 (84.5) |
| **Vaccine characteristics influencing vaccination acceptance** |  |  |  |  |  |  |
| ***Required doses of COVID-19 vaccine*** |  |  |  |  |  |  |
| Only accept single dose | 505 (62.3) | 575 (52.6) | 316 (23.0) | 734 (46.9) | 398 (39.1) | 281 (27.1) |
| Do not mind | 306 (37.7) | 519 (47.4) | 1057 (77.0) | 832 (53.1) | 621 (60.9) | 758 (72.9) |
| ***Effectiveness threshold of COVID-19 vaccine*** |  |  |  |  |  |  |
| Only accept 90% threshold | 355 (43.8) | 918 (83.9) | 828 (60.3) | 865 (55.2) | 842 (82.6) | 655 (63.2) |
| Do not mind | 456 (56.2) | 176 (16.1) | 545 (39.7) | 701 (44.8) | 177 (17.4) | 382 (36.8) |
| ***Adverse reaction of COVID-19 vaccine*** |  |  |  |  |  |  |
| Only accept minor adverse reactions | 363 (44.8) | 915 (83.6) | 1152 (83.9) | 1073 (68.5) | 840 (82.4) | 896 (86.4) |
| Do not mind moderate adverse reactions | 448 (55.2) | 179 (16.4) | 221 (16.1) | 493 (31.5) | 179 (17.6) | 141 (13.6) |
| ***Duration of COVID-19 vaccine protection*** |  |  |  |  |  |  |
| Only accept no lesser than 12 months | 637 (78.5) | 816 (74.6) | 708 (51.6) | 757 (48.3) | 660 (64.8) | 382 (36.8) |
| Do not mind | 174 (21.5) | 278 (25.4) | 665 (48.4) | 809 (51.7) | 359 (35.2) | 655 (63.2) |
| ***Technology used in COVID-19 vaccine*** |  |  |  |  |  |  |
| Do not accept mRNA technology | 216 (26.6) | 179 (16.4) | 145 (10.6) | 566 (36.1) | 103 (10.1) | 98 (9.5) |
| Do not mind | 479 (59.1) | 215 (19.7) | 495 (36.1) | 523 (33.4) | 188 (18.4) | 190 (18.3) |
| Do not know much about mRNA technology | 116 (14.3) | 700 (64.0) | 733 (53.4) | 477 (30.5) | 728 (71.4) | 749 (72.2) |
| ***Producing country of COVID-19 vaccine*** |  |  |  |  |  |  |
| Only accept a vaccine that is produced by specific countries | 682 (84.1) | 660 (60.3) | 656 (47.8) | 660 (42.1) | 743 (72.9) | 756 (72.9) |
| Producing countries of a COVID-19 vaccine is not of my concern in vaccine acceptance | 129 (15.9) | 434 (39.7) | 717 (52.2) | 906 (57.9) | 276 (27.1) | 281 (27.1) |
| **First foremost important vaccine characteristics influencing COVID-19 vaccine choice** |  |  |  |  |  |  |
| Effectiveness threshold | 416 (51.3) | 325 (29.7) | 419 (30.5) | 617 (39.4) | 16 (1.6) | 482 (46.5) |
| Adverse reactions | 253 (31.2) | 280 (25.6) | 676 (49.2) | 585 (37.4) | 296 (29.9) | 424 (40.9) |
| Duration of protection | 60 (7.4) | 191 (17.5) | 113 (8.2) | 139 (8.9) | 177 (17.9) | 7 (0.7) |
| Administration doses | 28 (3.5) | 143 (13.1) | 68 (5.0) | 145 (9.3) | 166 (16.8) | 23 (2.2) |
| Vaccination cost | 14 (1.7) | 101 (9.2) | 61 (4.4) | 40 (2.6) | 188 (19.0) | 43 (4.1) |
| Country or origin | 18 (2.2) | 31 (2.8) | 36 (2.6) | 18 (1.1) | 31 (3.1) | 55 (5.3) |
| mRNA technology | 22 (2.7) | 23 (2.1) | - | 22 (1.4) | 115 (11.6) | 3 (0.3) |
| **Second important vaccine characteristics influencing COVID-19 vaccine choice** |  |  |  |  |  |  |
| Adverse reactions | 147 (18.1) | 278 (25.4) | 387 (28.2) | 536 (34.2) | 175 (17.2) | 385 (37.1) |
| Duration of protection | 252 (31.1) | 304 (27.8) | 264 (18.5) | 413 (26.4) | 256 (25.1) | 92 (8.9) |
| Effectiveness threshold | 81 (10.0) | 160 (14.6) | 438 (31.9) | 350 (22.3) | 244 (23.9) | 337 (32.5) |
| Country of origin | 249 (30.7) | 52 (4.8) | 61 (4.4) | 25 (1.6) | 198 (19.4) | 90 (8.7) |
| Cost of vaccination | 32 (3.9) | 125 (11.4) | 142 (10.3) | 76 (4.9) | 79 (7.8) | 80 (7.7) |
| Administration doses | 25 (3.1) | 148 (13.5) | 91 (6.6) | 99 (6.3) | 26 (2.6) | 41 (4.0) |
| mRNA technology | 25 (3.1) | 67 (4.3) | - | 67 (4.3) | 41 (4.0) | 12 (1.2) |

|  | **Malaysia**  **n=2175** | **Norway**  **n=1382** | **Pakistan**  **n=1271** | **Singapore**  **n=841** | **Somalia**  **n=894** | **South Africa**  **n=1086** |
| --- | --- | --- | --- | --- | --- | --- |
| **Demographics** |  |  |  |  |  |  |
| ***Age group*** |  |  |  |  |  |  |
| 18-29 | 413 (19.0) | 303 (21.9) | 460 (36.2) | 73 (8.7) | 178 (19.9) | 194 (17.9) |
| 30-39 | 590 (27.1) | 390 (28.2) | 279 (22.0) | 231 (27.5) | 266 (29.8) | 267 (24.6) |
| 40-49 | 506 (23.3) | 295 (21.3) | 152 (12.0) | 211 (25.1) | 243 (27.2) | 254 (23.4) |
| 50-59 | 324 (14.9) | 232 (16.8) | 114 (9.0) | 185 (22.0) | 163 (18.2) | 186 (17.1) |
| 60 and above | 342 (15.7) | 162 (11.7) | 266 (20.9) | 141 (16.8) | 44 (4.9) | 185 (17.0) |
| ***Gender*** |  |  |  |  |  |  |
| Male | 814 (37.4) | 758 (54.8) | 652 (51.3) | 343 (40.8) | 442 (49.4) | 432 (39.8) |
| Female | 1361 (62.6) | 624 (45.2) | 619 (48.7) | 498 (59.2) | 452 (50.6) | 654 (60.2) |
| ***Highest education level*** |  |  |  |  |  |  |
| Secondary school and below | 356 (16.4) | 55 (4.0) | 353 (27.8) | 84 (10.0) | 239 (26.7) | 168 (15.5) |
| Certificate/A-Level/Diploma | 511 (23.5) | 471 (34.1) | 93 (7.3) | 150 (17.8) | 283 (31.7) | 405 (37.3) |
| Bachelor degree | 883 (40.6) | 489 (35.4) | 402 (31.6) | 413 (49.1) | 294 (32.9) | 224 (20.6) |
| Postgraduate degree | 425 (19.5) | 367 (26.6) | 423 (33.3) | 194 (23.1) | 78 (8.7) | 289 (26.6) |
| ***Ever delayed acceptance or refuse vaccine despite availability of vaccine service*** |  |  |  |  |  |  |
| Yes | 189 (8.7) | 141 (10.2) | 415 (32.7) | 112 (13.3) | 348 (38.9) | 79 (7.3) |
| No | 1986 (91.3) | 1241 (89.8) | 856 (67.3) | 729 (86.7) | 546 (61.1) | 1007 (92.7) |
| **Vaccine characteristics influencing vaccination acceptance** |  |  |  |  |  |  |
| ***Required doses of COVID-19 vaccine*** |  |  |  |  |  |  |
| Only accept single dose | 627 (28.8) | 424 (30.7) | 645 (50.7) | 109 (13.0) | 548 (61.3) | 298 (27.4) |
| Do not mind | 1548 (71.2) | 958 (69.3) | 626 (49.3) | 732 (87.0) | 346 (38.7) | 788 (72.6) |
| ***Effectiveness threshold of COVID-19 vaccine*** |  |  |  |  |  |  |
| Only accept 90% threshold | 1726 (79.4) | 811 (58.7) | 846 (66.6) | 641 (76.2) | 529 (59.2) | 434 (40.0) |
| Do not mind | 449 (20.6) | 571 (41.3) | 425 (33.4) | 200 (23.8) | 365 (40.8) | 652 (60.0) |
| ***Adverse reactions of COVID-19 vaccine*** |  |  |  |  |  |  |
| Only accept minor adverse reactions | 1743 (80.1) | 685 (49.6) | 928 (73.0) | 610 (72.5) | 620 (69.4) | 456 (42.0) |
| Do not mind moderate adverse reactions | 432 (19.1) | 697 (50.4) | 343 (27.0) | 231 (27.5) | 274 (30.6) | 630 (58.0) |
| ***Duration of COVID-19 vaccine protection*** |  |  |  |  |  |  |
| Only accept no lesser than 12 months | 1503 (69.1) | 676 (48.9) | 675 (53.1) | 566 (67.3) | 508 (56.8) | 457 (42.1) |
| Do not mind | 672 (30.9) | 706 (51.1) | 596 (46.9) | 275 (32.7) | 386 (43.2) | 629 (57.9) |
| ***Technology used in COVID-19 vaccine*** |  |  |  |  |  |  |
| Do not accept mRNA technology | 313 (14.4) | 202 (14.6) | 222 (17.5) | 51 (6.1) | 433 (48.4) | 106 (9.8) |
| Do not mind | 511 (23.5) | 585 (42.3) | 317 (24.9) | 266 (31.6) | 289 (32.3) | 382 (35.2) |
| Do not know much about mRNA technology | 1351 (62.1) | 595 (43.1) | 732 (57.6) | 524 (62.3) | 172 (19.2) | 598 (55.1) |
| ***Producing country of COVID-19 vaccine*** |  |  |  |  |  |  |
| Only accept a vaccine that is produced by specific countries | 1151 (52.9) | 1106 (80.0) | 736 (57.9) | 535 (63.6) | 454 (50.8) | 317 (29.2) |
| Producing countries of a COVID-19 vaccine is not of my concern in vaccine acceptance | 1024 (47.1) | 276 (20.0) | 535 (42.1) | 306 (36.4) | 440 (49.2) | 769 (70.8) |
| **First foremost important vaccine characteristics influencing COVID-19 vaccine choice** |  |  |  |  |  |  |
| Effectiveness threshold | 1085 (49.9) | 664 (48.0) | 423 (33.3) | 388 (46.1) | 273 (29.1) | 441 (40.6) |
| Adverse reactions | 723 (33.2) | 386 (27.9) | 507 (39.9) | 311 (37.0) | 228 (24.3) | 276 (25.4) |
| Duration of protection | 122 (5.6) | 103 (7.5) | 104 (8.2) | 35 (4.2) | 126 (13.4) | 210 (19.3) |
| Administration doses | 68 (5.0) | 118 (8.5) | 100 (7.9) | 8 (1.0) | 74 (7.9) | 77 (7.1) |
| Vaccination cost | 82 (3.8) | 10 (0.7) | 63 (5.0) | 19 (2.3) | 57 (6.1) | 22 (2.0) |
| Country or origin | 70 (3.2) | 20 (1.4) | 41 (3.2) | 58 (6.9) | 116 (12.4) | 26 (2.4) |
| mRNA technology | 62 (2.9) | 81 (5.9) | 33 (2.6) | 22 (2.6) | 64 (6.8) | 34 (3.1) |
| **Second important vaccine characteristics influencing COVID-19 vaccine choice** |  |  |  |  |  |  |
| Adverse reactions | 765 (35.2) | 98 (7.1) | 296 (23.3) | 257 (30.6) | 203 (21.6) | 264 (24.3) |
| Duration of protection | 475 (21.8) | 275 (19.9) | 315 (24.8) | 202 (24.0) | 199 (21.2) | 411 (37.8) |
| Effectiveness threshold | 572 (26.3) | 41 (3.0) | 202 (15.9) | 208 (24.7) | 119 (12.7) | 190 (17.5) |
| Country of origin | 61 (4.4) | 789 (57.1) | 144 (11.3) | 86 (10.2) | 147 (15.7) | 72 (6.6) |
| Cost of vaccination | 142 (10.3) | 112 (8.1) | 131 (10.3) | 30 (3.6) | 158 (16.8) | 29 (2.7) |
| Administration doses | 77 (3.5) | 18 (1.3) | 152 (12.0) | 24 (2.9) | 67 (7.1) | 96 (8.8) |
| mRNA technology | 55 (2.5) | 49 (3.5) | 31 (2.4) | 34 (4.0) | 45 (4.8) | 24 (2.2) |

|  | **Sri Lanka**  **n=776** | **United Arab Emirates**  **n=938** | **United Kingdom**  **n=1021** | **United States of America**  **n=968** | **Vietnam**  **n=1462** |
| --- | --- | --- | --- | --- | --- |
| **Demographics** |  |  |  |  |  |
| ***Age group*** |  |  |  |  |  |
| 18-29 | 260 (33.5) | 170 (18.1) | 244 (23.9) | 158 (16.3) | 467 (31.9) |
| 30-39 | 115 (14.8) | 293 (31.2) | 399 (39.1) | 323 (33.4) | 543 (37.1) |
| 40-49 | 208 (26.8) | 268 (28.6) | 182 (17.8) | 250 (25.8) | 231 (15.8) |
| 50-59 | 167 (21.5) | 130 (13.9) | 148 (14.5) | 121 (12.5) | 144 (9.8) |
| 60 and above | 26 (3.4) | 77 (8.2) | 48 (4.7) | 116 (12.0) | 77 (5.3) |
| ***Gender*** |  |  |  |  |  |
| Male | 199 (25.6) | 459 (48.9) | 564 (55.2) | 471 (48.7) | 757 (51.8) |
| Female | 577 (74.4) | 479 (51.1) | 457 (44.8) | 485 (50.1) | 705 (48.2) |
| Other |  |  |  | 12 (1.2) |  |
| ***Highest education level*** |  |  |  |  |  |
| Secondary school and below | 16 (2.1) | 240 (25.6) | 7 (0.7) | 115 (11.9) | 269 (18.4) |
| Certificate/A-Level/Diploma | 202 (26.0) | 232 (24.7) | 298 (29.2) | 317 (32.7) | 470 (32.1) |
| Bachelor degree | 462 (59.5) | 376 (40.1) | 342 (33.5) | 441 (45.6) | 582 (39.8) |
| Postgraduate degree | 96 (12.4) | 90 (9.6) | 374 (36.6) | 95 (9.8) | 141 (9.6) |
| ***Ever delayed acceptance or refuse vaccine despite availability of vaccine service*** |  |  |  |  |  |
| Yes | 62 (8.0) | 355 (37.8) | 87 (8.5) | 140 (14.5) | 110 (7.5) |
| No | 714 (92.0) | 583 (62.2) | 934 (91.5) | 828 (85.5) | 1352 (92.5) |
| **Vaccine characteristics influencing vaccination acceptance** |  |  |  |  |  |
| ***Required doses of COVID-19 vaccine*** |  |  |  |  |  |
| Only accept single dose | 472 (60.8) | 366 (39.0) | 362 (35.5) | 478 (49.4) | 887 (60.7) |
| Do not mind | 304 (39.2) | 572 (61.0) | 659 (64.5) | 490 (50.6) | 575 (39.3) |
| ***Effectiveness threshold of COVID-19 vaccine*** |  |  |  |  |  |
| Only accept 90% threshold | 460 (59.3) | 487 (51.9) | 582 (57.0) | 487 (50.3) | 1159 (79.3) |
| Do not mind | 316 (40.7) | 451 (48.1) | 439 (43.0) | 481 (49.7) | 303 (20.7) |
| ***Adverse reactions of COVID-19 vaccine*** |  |  |  |  |  |
| Only accept minor adverse reactions | 621 (80.0) | 394 (74.0) | 864 (84.6) | 497 (51.3) | 1045 (71.5) |
| Do not mind moderate adverse reactions | 155 (20.0) | 244 (26.0) | 157 (15.4) | 471 (48.7) | 417 (28.5) |
| ***Duration of COVID-19 vaccine protection*** |  |  |  |  |  |
| Only accept no lesser than 12 months | 523 (67.4) | 477 (50.9) | 520 (50.9) | 679 (70.1) | 908 (62.1) |
| Do not mind | 253 (32.6) | 461 (49.1) | 501 (49.1) | 289 (29.9) | 554 (37.9) |
| ***Technology used in COVID-19 vaccine*** |  |  |  |  |  |
| Do not accept mRNA technology | 358 (46.1) | 284 (30.3) | 101 (9.9) | 189 (19.2) | 467 (31.9) |
| Do not mind | 159 (20.5) | 350 (37.3) | 385 (37.7) | 488 (50.4) | 322 (22.0) |
| Do not know much about mRNA technology | 259 (33.4) | 304 (32.4) | 535 (52.4) | 294 (30.4) | 673 (46.0) |
| ***Producing country of COVID-19 vaccine*** |  |  |  |  |  |
| Only accept a vaccine that is produced by specific countries | 509 (65.6) | 461 (49.1) | 771 (75.5) | 727 (75.1) | 995 (68.1) |
| Producing countries of a COVID-19 vaccine is not of my concern in vaccine acceptance | 267 (34.4) | 477 (50.9) | 250 (24.5) | 241 (24.9) | 467 (31.9) |
| **First foremost important vaccine characteristics influencing COVID-19 vaccine choice** |  |  |  |  |  |
| Effectiveness threshold | 359 (46.3) | 264 (29.5) | 616 (60.3) | 536 (34.7) | 595 (40.7) |
| Adverse reactions | 280 (36.1) | 286 (32.0) | 193 (18.9) | 384 (39.7) | 299 (20.5) |
| Duration of protection | 70 (9.0) | 99 (11.1) | 37 (3.6) | 74 (7.6) | 81 (5.5) |
| Administration doses | 36 (4.6) | 73 (8.2) | 74 (7.2) | 55 (5.7) | 204 (14.0) |
| Vaccination cost | 10 (1.3) | 62 (6.9) | 10 (1.0) | 38 (3.9) | 87 (6.0) |
| Country of origin | 6 (0.8) | 83 (9.3) | 43 (4.2) | 38 (3.9) | 171 (11.7) |
| mRNA technology | 15 (1.9) | 27 (3.0) | 48 (4.7) | 43 (4.4) | 25 (1.7) |
| **Second important vaccine characteristics influencing COVID-19 vaccine choice** |  |  |  |  |  |
| Adverse reactions | 295 (38.0) | 189 (21.1) | 262 (25.7) | 179 (18.5) | 424 (29.0) |
| Duration of protection | 203 (26.2) | 181 (20.2) | 393 (38.5) | 231 (23.9) | 174 (11.9) |
| Effectiveness threshold | 194 (25.0) | 161 (18.0) | 26 (2.5) | 104 (10.7) | 302 (20.7) |
| Country of origin | 24 (3.1) | 88 (9.8) | 250 (24.5) | 258 (26.7) | 178 (12.2) |
| Cost of vaccination | 13 (1.7) | 166 (18.6) | 23 (2.3) | 73 (7.5) | 185 (12.7) |
| Administration doses | 20 (2.6) | 82 (9.2) | 43 (4.2) | 93 (9.6) | 128 (8.8) |
| mRNA technology | 27 (3.5) | 27 (3.0) | 24 (2.4) | 30 (3.1) | 71 (4.9) |
