# Supplementary material for: COVID-19 vaccination intention and vaccine characteristics influencing vaccination acceptance: a global survey of 17 countries
Source: Infect Dis Poverty. 2021 Oct 7;10:122. doi: 10.1186/s40249-021-00900-w (PMC8496428; doi:10.1186/s40249-021-00900-w)
Supplement: Supplementary file 5 — Additional file 5. Factors influencing COVID-19 vaccine hesitancy by region. [file 40249_2021_900_MOESM5_ESM.docx]

Factors influencing COVID–19 vaccine hesitancy by WHO region

|  | **Unlikely/ Extremely unlikely to accept COVID-19 vaccine** | | | | | |
| --- | --- | --- | --- | --- | --- | --- |
|  | **African^a^**  **(n=93)** | **Eastern Mediterranean^b^**  **(n=825)** | **European^c^**  **(n=149)** | **Region of the Americas^d^**  **(n=284)** | **Southeast Asia^f^**  **(n=221)** | **Western Pacific^g^**  **(n=946)** |
| Demographics |  |  |  |  |  |  |
| **Age group** |  |  |  |  |  |  |
| 18–29 | 3.68 (1.60–8.45)** | 1 (ref) | 1 (ref) | 1 (ref) | 1.39 (0.71–2.71) | 1 (ref) |
| 30–39 | 1.53 (0.66–3.56) | 1.03 (0.83–1.28) | 1.68 (0.94–2.98) | 0.83 (0.53–1.31) | 1.12 (0.56–2.23) | 1.66 (1.33–2.07)*** |
| 40–49 | 1.46 (0.62–3.46) | 1.03 (0.82–1.31) | 1.42 (0.75–2.69) | 0.80 (0.89–1.30) | 0.92 (0.44–1.90) | 2.07 (1.65–2.61)*** |
| 50–59 | 2.05 (0.87–4.85) | 1.07 (0.82–1.40) | 3.35 (1.87–6.01)*** | 1.53 (0.89–2.61) | 1.10 (0.52–2.32) | 2.68 (2.09–3.42)*** |
| 60 and above | 1 (ref) | 1.76 (1.35–2.29)*** | 3.17 (1.66–6.07)*** | 4.09 (2.38–7.03)*** | 1 (ref) | 3.32 (2.60–4.24)*** |
| **Gender** |  |  |  |  |  |  |
| Male | 1.06 (0.65–1.71) | 1 (ref) | 1.03 (0.72–1.48) | 1.67 (1.23–2.28)** | 1.55 (1.16–2.07)** | 1 (ref) |
| Female | 1 (ref) | 1.08 (0.92–1.26) | 1 (ref) | 1 (ref) | 1 (ref) | 1.30 (1.13–1.50)*** |
| Other | - | - | - | - | - | - |
| **Highest education level** |  |  |  |  |  |  |
| Secondary school and below | 1 (ref) | 1.73 (1.36–2.21)*** | 3.30 (1.56–6.98)** | 2.48 (1.35–4.55)** | 2.54 (1.62–4.00)*** | 1.98 (1.57–2.50)*** |
| Certificate/A–Level/Diploma | 1.02 (0.46–2.26) | 1.02 (0.79–1.32) | 0.82 (0.52–1.29) | 0.78 (0.46–1.31) | 0.73 (0.45–1.18) | 1.14 (0.91–1.43) |
| Bachelor degree | 1.43 (0.65–3.13) | 0.98 (0.79–1.23) | 0.97 (0.63–1.51) | 0.63 (0.38–1.05) | 1.17 (0.84–1.65) | 1.01 (0.82–1.25) |
| Postgraduate degree | 2.06 (0.95–4.43) | 1 (ref) | 1 (ref) | 1 (ref) | 1 (ref) | 1 (ref) |
| **Ever delayed acceptance or refuse vaccine despite availability of vaccine service** |  |  |  |  |  |  |
| Yes | 15.16 (8.83–26.02)*** | 1.31 (1.12–1.53)** | 5.56 (3.72–8.33)*** | 0.88 (0.58–1.35) | 3.41 (2.54–4.58)*** | 3.29 (2.78–3.90)*** |
| No | 1 (ref) | 1 (ref) | 1 (ref) | 1 (ref) | 1 (ref) | 1 (ref) |

^a^Hosmer–Lemeshow test, chi–square: 7.552, *P-*value: 0.478; Nagelkerke *R^2^* : 0.212

^b^Hosmer–Lemeshow test, chi–square: 53.177, *P-*value: : *P*<0.001; Nagelkerke *R^2^* : 0.039

^c^Hosmer–Lemeshow test, chi–square: 36.248, *P-*value: *P*<0.001; Nagelkerke *R^2^* : 0.134

^d^Hosmer–Lemeshow test, chi–square: 12.606, *P-*value: 0.126; Nagelkerke *R^2^* : 0.163

^e^Hosmer–Lemeshow test, chi–square: 28.174, *P-*value: *P*<0.001; Nagelkerke *R^2^* : 0.085

^f^Hosmer–Lemeshow test, chi–square: 10.189, *P-*value: 0.252; Nagelkerke *R^2^* : 0.090
