# Supplementary material for: COVID-19 vaccination intention and vaccine characteristics influencing vaccination acceptance: a global survey of 17 countries
Source: Infect Dis Poverty. 2021 Oct 7;10:122. doi: 10.1186/s40249-021-00900-w (PMC8496428; doi:10.1186/s40249-021-00900-w)
Supplement: Supplementary file 6 — Additional file 6. Factors influencing COVID-19 vaccine hesitancy by country. [file 40249_2021_900_MOESM6_ESM.docx]

Factors influencing COVID–19 vaccine hesitancy by country

| Australia | All participants  n=(811) | Extremely unlikely/ unlikely *vs* extremely likely/likely to accept COVID–19 vaccination  (n=29) | |
| --- | --- | --- | --- |
|  |  | OR (95% *CI*) | *P*–value |
| Demographics |  |  |  |
| ***Age group*** |  |  |  |
| 18–29 | 123 (15.2) | 1.31 (0.22–7.94) | 0.769 |
| 30–39 | 265 (32.7) | 0.61 (0.10–3.65) | 0.587 |
| 40–49 | 277 (34.2) | 0.30 (0.05–1.74) | 0.177 |
| 50–59 | 103 (12.7) | 0.91 (0.16–5.23) | 0.915 |
| 60 and above | 43 (5.3) | 1 (ref) |  |
| ***Gender*** |  |  |  |
| Male | 423 (52.2) | 2.25 (0.85–6.00) | 0.105 |
| Female | 388 (47.8) | 1 (ref) |  |
| ***Highest education level*** |  |  |  |
| Secondary school and below | 36 (4.4) | 48.28 (5.68–410.29) | *P*<0.001 |
| Certificate/A-Level/Diploma | 276 (34.0) | 2.80 (0.37–21.11) | 0.319 |
| Bachelor degree | 412 (50.8) | 2.39 (0.38–15.12) | 0.354 |
| Postgraduate degree | 87 (10.7) | 1 (ref) |  |
| ***Ever delayed acceptance or refuse vaccine despite availability of vaccine service*** |  |  |  |
| Yes | 90 (11.1) | 2.00 (0.67–6.02) | 0.217 |
| No | 721 (88.9) | 1 (ref) |  |
| Vaccine characteristics influencing vaccination acceptance |  |  |  |
| ***Required doses of COVID-19 vaccine*** |  |  |  |
| Only accept single dose | 505 (62.3) | 2.92 (0.97–8.75) | 0.056 |
| Do not mind | 306 (37.7) | 1 (ref) |  |
| ***Effectiveness threshold of COVID-19 vaccine*** |  |  |  |
| Only accept 90% threshold | 355 (43.8) | 3.97 (1.42–11.11) | 0.009 |
| Do not mind | 456 (56.2) | 1 (ref) |  |
| ***Adverse reactions of COVID-19 vaccine*** |  |  |  |
| Only accept minor adverse reactions | 363 (44.8) | 10.11 (2.65–38.55) | 0.001 |
| Do not mind moderate adverse reaction | 448 (55.2) | 1 (ref) |  |
| ***Duration of COVID-19 vaccine protection*** |  |  |  |
| Only accept lesser than 12 months | 637 (78.5) | 6.56 (1.35–32.00) | 0.020 |
| Do not mind | 174 (21.5) | 1 (ref) |  |
| ***Technology used in COVID-19 vaccine*** |  |  |  |
| Do not accept mRNA technology | 216 (26.6) | 9.48 (2.04–43.96) | 0.004 |
| Do not mind | 479 (59.1) | 0.67 (0.11–4.15) | 0.670 |
| Do not know mRNA technology | 116 (14.3) | 1 (ref) |  |
| ***Producing country of COVID-19 vaccine*** |  |  |  |
| Only accept a vaccine that is produced by specific countries | 682 (84.1) | 1.19 (0.33–4.28) | 0.787 |
| Producing countries of a COVID-19 vaccine is not of my concern in vaccine acceptance | 129 (15.9) | 1 (ref) |  |

Hosmer–Lemeshow test, chi–square: 1.710, *P*–value: 0.989; Nagelkerke *R^2^* : 0.467

| Bangladesh | All participants  n=(1094) | Extremely unlikely/ unlikely *vs* extremely likely/likely to accept COVID–19 vaccination  (n=86) | |
| --- | --- | --- | --- |
|  |  | OR (95% CI) | p–value |
| Demographics |  |  |  |
| ***Age group*** |  |  |  |
| 18–29 | 470 (43.0) | 0.41 (0.14–1.17) | 0.097 |
| 30–39 | 395 (36.1) | 0.84 (0.30–2.32) | 0.737 |
| 40–49 | 125 (11.4) | 0.98 (0.33–2.93) | 0.507 |
| 50–59 | 49 (4.5) | 1.41 (0.40–4.99) | 0.595 |
| 60 and above | 55 (5.0) | 1 (ref) |  |
| ***Gender*** |  |  |  |
| Male | 551 (50.4) | 1.78 (1.04–3.04) | 0.035 |
| Female | 543 (49.6) | 1 (ref) |  |
| ***Highest education level*** |  |  |  |
| Secondary school and below | 97 (8.9) | 7.63 (3.97–14.68) | p<0.001 |
| Certificate/A–Level/Diploma | 79 (7.2) | 0.79 (0.23–2.78) | 0.719 |
| Bachelor degree | 403 (36.8) | 1.26 (0.71–2.27) | 0.431 |
| Postgraduate degree | 515 (47.1) | 1 (ref) |  |
| *Ever delayed acceptance or refuse vaccine despite* availability of vaccine service |  |  |  |
| Yes | 356 (32.5) | 1.17 (0.69–1.97) | 0.566 |
| No | 738 (67.5) | 1 (ref) |  |
| Vaccine characteristics influencing vaccination acceptance |  |  |  |
| ***Required doses of COVID–19 vaccine*** |  |  |  |
| Only accept single dose | 575 (52.6) | 1.51 (0.86–2.65) | 0.151 |
| Do not mind | 519 (47.4) | 1 (ref) |  |
| ***Effectiveness threshold of COVID–19 vaccine*** |  |  |  |
| Only accept 90% threshold | 918 (83.9) | 1.17 (0.61–2.25) | 0.630 |
| Do not mind | 176 (16.1) | 1 (ref) |  |
| ***Adverse reactions of COVID–19 vaccine*** |  |  |  |
| Only accept minor adverse reactions | 915 (83.6) | 1.32 (0.58–3.01) | 0.515 |
| Do not mind moderate adverse reactions | 179 (16.4) | 1 (ref) |  |
| ***Duration of COVID–19 vaccine protection*** |  |  |  |
| Only accept lesser than 12 months | 816 (74.6) | 1.27 (0.58–2.78) | 0.549 |
| Do not mind | 278 (25.4) | 1 (ref) |  |
| ***Technology used in COVID–19 vaccine*** |  |  |  |
| Do not accept mRNA technology | 179 (16.4) | 0.85 (0.42–1.72) | 0.657 |
| Do not mind | 215 (19.7) | 0.97 (0.49–1.93) | 0.937 |
| Do not know mRNA technology | 700 (64.0) | 1 (ref) |  |
| ***Producing country of COVID–19 vaccine*** |  |  |  |
| Only accept a vaccine that is produced by specific countries | 660 (60.3) | 0.79 (0.46–1.34) | 0.380 |
| Producing countries of a COVID–19 vaccine is not of my concern in vaccine acceptance | 434 (39.7) | 1 (ref) |  |

Hosmer–Lemeshow test, chi–square: 10.800, p–value: 0.213; Nagelkerke R^2^ : 0.180

| China | All participants  n=(1373) | Extremely unlikely/ unlikely *vs* extremely likely/likely to accept COVID–19 vaccination  (n=65) | |
| --- | --- | --- | --- |
|  |  | OR (95% CI) | p–value |
| Demographics |  |  |  |
| ***Age group*** |  |  |  |
| 18–29 | 760 (55.4) | 0.26 (0.09–0.78) | 0.016 |
| 30–39 | 357 (26.0) | 0.35 (0.11–1.08) | 0.066 |
| 40–49 | 134 (9.8) | 0.67 (0.21–2.16) | 0.507 |
| 50–59 | 77 (5.6) | 0.28 (0.06–1.31) | 0.255 |
| 60 and above | 45 (3.3) | 1 (ref) |  |
| ***Gender*** |  |  |  |
| Male | 593 (43.2) | 1.37 (0.80–2.36) | 0.255 |
| Female | 780 (56.8) | 1 (ref) |  |
| ***Highest education level*** |  |  |  |
| Secondary school and below | 177 (12.9) | 0.78 (0.30–2.05) | 0.614 |
| Certificate/A–Level/Diploma | 243 (17.7) | 0.81 (0.35–1.89) | 0.624 |
| Bachelor degree | 693 (50.5) | 0.86 (0.42–1.76) | 0.688 |
| Postgraduate degree | 260 (18.9) | 1 (ref) |  |
| ***Ever delayed acceptance or refuse vaccine despite* availability of vaccine service** |  |  |  |
| Yes | 302 (22.0) | 3.48 (2.00–6.07) | p<0.001 |
| No | 1071 (78.0) | 1 (ref) |  |
| Vaccine characteristics influencing vaccination acceptance |  |  |  |
| ***Required doses of COVID–19 vaccine*** |  |  |  |
| Only accept single dose | 316 (23.0) | 2.21 (1.22–3.99) | 0.009 |
| Do not mind | 1057 (77.0) | 1 (ref) |  |
| ***Effectiveness threshold of COVID–19 vaccine*** |  |  |  |
| Only accept 90% threshold | 828 (60.3) | 1.17 (0.61–2.25) | 0.630 |
| Do not mind | 545 (39.7) | 1 (ref) |  |
| ***Adverse reactions of COVID–19 vaccine*** |  |  |  |
| Only accept minor adverse reactions | 1152 (83.9) | 0.49 (0.25–0.97) | 0.039 |
| Do not mind moderate adverse reactions | 221 (16.1) | 1 (ref) |  |
| ***Duration of COVID–19 vaccine protection*** |  |  |  |
| Only accept lesser than 12 months | 708 (51.6) | 0.93 (0.51–1.70) | 0.811 |
| Do not mind | 665 (48.4) | 1 (ref) |  |
| ***Technology used in COVID–19 vaccine*** |  |  |  |
| Do not accept mRNA technology | 145 (10.6) | 1.77 (0.88–3.58) | 0.111 |
| Do not mind | 495 (36.1) | 0.73 (0.39–1.39) | 0.337 |
| Do not know mRNA technology | 733 (53.4) | 1 (ref) |  |
| ***Producing country of COVID–19 vaccine*** |  |  |  |
| Only accept a vaccine that is produced by specific countries | 656 (47.8) | 0.64 (0.37–1.10) | 0.108 |
| Producing countries of a COVID–19 vaccine is not of my concern in vaccine acceptance | 717 (52.2) | 1 (ref) |  |

Hosmer–Lemeshow test, chi–square: 4.939, p–value: 0.764; Nagelkerke R^2^ : 0.149

| India | All participants  n=(1094) | Extremely unlikely/ unlikely *vs* extremely likely/likely to accept COVID–19 vaccination  (n=76) | |
| --- | --- | --- | --- |
|  |  | OR (95% CI) | p–value |
| Demographics |  |  |  |
| ***Age group*** |  |  |  |
| 18–29 | 481 (30.7) | 0.97 (0.27–3.44) | 0.958 |
| 30–39 | 324 (20.7) | 0.77 (0.20–3.00) | 0.709 |
| 40–49 | 323 (20.6) | 1.03 (0.27–3.96) | 0.964 |
| 50–59 | 307 (19.6) | 1.19 (0.31–4.53) | 0.799 |
| 60 and above | 131 (8.4) | 1 (ref) |  |
| ***Gender*** |  |  |  |
| Male | 743 (47.4) | 1.21 (0.73–2.01) | 0.452 |
| Female | 823 (52.6) | 1 (ref) |  |
| ***Highest education level*** |  |  |  |
| Secondary school and below | 133 (8.5) | 0.82 (0.31–2.17) | 0.691 |
| Certificate/A–Level/Diploma | 422 (26.9) | 0.52 (0.24–1.15) | 0.108 |
| Bachelor degree | 560 (35.8) | 0.88 (0.49–1.58) | 0.670 |
| Postgraduate degree | 451 (28.8) | 1 (ref) |  |
| ***Ever delayed acceptance or refuse vaccine despite* availability of vaccine** service |  |  |  |
| Yes | 150 (9.6) | 9.48 (5.58–16.11) | p<0.001 |
| No | 1416 (90.4) | 1 (ref) |  |
| Vaccine characteristics influencing vaccination acceptance |  |  |  |
| ***Required doses of COVID–19 vaccine*** |  |  |  |
| Only accept single dose | 734 (46.9) | 1.13 (0.65–1.96) | 0.667 |
| Do not mind | 832 (53.1) | 1 (ref) |  |
| ***Effectiveness threshold of COVID–19 vaccine*** |  |  |  |
| Only accept 90% threshold | 865 (55.2) | 3.03 (1.43–6.41) | 0.004 |
| Do not mind | 701 (44.8) | 1 (ref) |  |
| ***Adverse reactions of COVID–19 vaccine*** |  |  |  |
| Only accept minor adverse reactions | 1073 (68.5) | 1.03 (0.50–2.24) | 0.945 |
| Do not mind moderate adverse reactions | 493 (31.5) | 1 (ref) |  |
| ***Duration of COVID–19 vaccine protection*** |  |  |  |
| Only accept lesser than 12 months | 757 (48.3) | 1.23 (0.68–2.24) | 0.491 |
| Do not mind | 809 (51.7) | 1 (ref) |  |
| ***Technology used in COVID–19 vaccine*** |  |  |  |
| Do not accept mRNA technology | 566 (36.1) | 0.52 (0.28–0.98) | 0.043 |
| Do not mind | 523 (33.4) | 0.49 (0.25–0.95) | 0.034 |
| Do not know mRNA technology | 477 (30.5) | 1 (ref) |  |
| ***Producing country of COVID–19 vaccine*** |  |  |  |
| Only accept a vaccine that is produced by specific countries | 660 (42.1) | 0.97 (0.58–1.64) | 0.915 |
| Producing countries of a COVID–19 vaccine is not of my concern in vaccine acceptance | 906 (57.9) | 1 (ref) |  |

Hosmer–Lemeshow test, chi–square: 7.376, p–value: 0.497; Nagelkerke R^2^ : 0.222

| Iran | All participants  n=(1019) | Extremely unlikely/ unlikely *vs* extremely likely/likely to accept COVID–19 vaccination  (n=284) | |
| --- | --- | --- | --- |
|  |  | OR (95% CI) | p–value |
| Demographics |  |  |  |
| ***Age group*** |  |  |  |
| 18–29 | 297 (29.1) | 0.67 (0.33–1.33) | 0.249 |
| 30–39 | 287 (28.2) | 0.99 (0.49–2.00) | 0.984 |
| 40–49 | 204 (20.0) | 1.30 (0.64–2.64) | 0.468 |
| 50–59 | 134 (13.2) | 0.87 (0.41–1.88) | 0.729 |
| 60 and above | 97 (9.5) | 1 (ref) |  |
| ***Gender*** |  |  |  |
| Male | 488 (47.9) | 1.15 (0.80–1.65) | 0.445 |
| Female | 531 (52.1) | 1 (ref) |  |
| ***Highest education level*** |  |  |  |
| Secondary school and below | 48 (4.7) | 0.10 (0.03–0.40) | 0.001 |
| Certificate/A–Level/Diploma | 205 (20.1) | 0.59 (0.35–0.99) | 0.047 |
| Bachelor degree | 490 (48.1) | 0.77 (0.51–1.17) | 0.219 |
| Postgraduate degree | 276 (27.1) | 1 (ref) |  |
| ***Ever delayed acceptance or refuse vaccine despite* availability of vaccine service** |  |  |  |
| Yes | 715 (70.2) | 0.07 (0.45–0.11) | p<0.001 |
| No | 304 (29.8) | 1 (ref) |  |
| Vaccine characteristics influencing vaccination acceptance |  |  |  |
| ***Required doses of COVID–19 vaccine*** |  |  |  |
| Only accept single dose | 398 (39.1) | 1.46 (0.99–2.14) | 0.051 |
| Do not mind | 621 (60.9) | 1 (ref) |  |
| ***Effectiveness threshold of COVID–19 vaccine*** |  |  |  |
| Only accept 90% threshold | 842 (82.6) | 0.60 (0.33–1.06) | 0.080 |
| Do not mind | 177 (17.4) | 1 (ref) |  |
| ***Adverse reactions of COVID–19 vaccine*** |  |  |  |
| Only accept minor adverse reactions | 840 (82.4) | 1.67 (0.94–2.97) | 0.083 |
| Do not mind moderate adverse reactions | 179 (17.6) | 1 (ref) |  |
| ***Duration of COVID–19 vaccine protection*** |  |  |  |
| Only accept lesser than 12 months | 660 (64.8) | 1.38 (0.91–2.10) | 0.130 |
| Do not mind | 359 (35.2) | 1 (ref) |  |
| ***Technology used in COVID–19 vaccine*** |  |  |  |
| Do not accept mRNA technology | 103 (10.1) | 0.87 (0.50–1.52) | 0.625 |
| Do not mind | 188 (18.4) | 1.80 (1.13–2.85) | 0.013 |
| Do not know mRNA technology | 728 (71.4) | 1 (ref) |  |
| ***Producing country of COVID–19 vaccine*** |  |  |  |
| Only accept a vaccine that is produced by specific countries | 743 (72.9) | 2.22 (1.44–3.42) | p<0.001 |
| Producing countries of a COVID–19 vaccine is not of my concern in vaccine acceptance | 276 (27.1) | 1 (ref) |  |

Hosmer–Lemeshow test, chi–square: 13.286, p–value: 0.102; Nagelkerke R^2^ : 0.411

| Japan | All participants  n=(1037) | Extremely unlikely/ unlikely *vs* extremely likely/likely to accept COVID–19 vaccination  n= 1037 | |
| --- | --- | --- | --- |
|  |  | OR (95% CI) | p–value |
| Demographics |  |  |  |
| ***Age group*** |  |  |  |
| 18–29 | 182 (17.6) | 1.06 (0.65–1.73) | 0.822 |
| 30–39 | 200 (19.3) | 1.80 (1.15–2.84) | 0.011 |
| 40–49 | 207 (20.0) | 2.34 (1.51–3.62) | p<0.001 |
| 50–59 | 167 (16.1) | 1.58 (0.98–2.53) | 0.059 |
| 60 and above | 281 (27.1) | 1 (ref) |  |
| ***Gender*** |  |  |  |
| Male | 456 (44.0) | 0.40 (0.29–0.55) | p<0.001 |
| Female | 581 (56.0) | 1 (ref) |  |
| ***Highest education level*** |  |  |  |
| Secondary school and below | 237 (22.9) | 0.94 (0.57–1.55) | 0.461 |
| Certificate/A–Level/Diploma | 199 (19.2) | 0.79 (0.47–1.33) | 0.643 |
| Bachelor degree | 417 (40.2) | 0.85 (0.55–1.33) | 0.799 |
| Postgraduate degree | 184 (17.7) | 1 (ref) |  |
| ***Ever delayed acceptance or refuse vaccine despite availability of vaccine service*** |  |  |  |
| Yes | 161 (15.5) | 3.14 (2.10–4.69) | p<0.001 |
| No | 876 (84.5) | 1 (ref) |  |
| Vaccine characteristics influencing vaccination acceptance |  |  |  |
| ***Required doses of COVID–19 vaccine*** |  |  |  |
| Only accept single dose | 281 (27.1) | 4.46 (3.11–6.41) | p<0.001 |
| Do not mind | 758 (72.9) | 1 (ref) |  |
| ***Effectiveness threshold of COVID–19 vaccine*** |  |  |  |
| Only accept 90% threshold | 655 (63.2) | 1.23 (0.85–1.79) | 0.275 |
| Do not mind | 382 (36.8) | 1 (ref) |  |
| ***Adverse reactions of COVID–19 vaccine*** |  |  |  |
| Only accept minor adverse reactions | 896 (86.4) | 0.75 (0.47–1.21) | 0.239 |
| Do not mind moderate adverse reactions | 141 (13.6) | 1 (ref) |  |
| ***Duration of COVID–19 vaccine protection*** |  |  |  |
| Only accept lesser than 12 months | 382 (36.8) | 0.96 (0.68–1.37) | 0.835 |
| Do not mind | 655 (63.2) | 1 (ref) |  |
| ***Technology used in COVID–19 vaccine*** |  |  |  |
| Do not accept mRNA technology | 98 (9.5) | 1.43 (0.87–2.33) | 0.158 |
| Do not mind | 190 (18.3) | 0.33 (0.20–0.53) | p<0.001 |
| Do not know mRNA technology | 749 (72.2) | 1 (ref) |  |
| ***Producing country of COVID–19 vaccine*** |  |  |  |
| Only accept a vaccine that is produced by specific countries | 756 (72.9) | 0.78 (0.55–1.10) | 0.159 |
| Producing countries of a COVID–19 vaccine is not of my concern in vaccine acceptance | 281 (27.1) | 1 (ref) |  |

Hosmer–Lemeshow test, chi–square: 4.248, p–value: 0.834; Nagelkerke R^2^ : 0.293

| Malaysia | All participants  n=(2175) | Extremely unlikely/ unlikely *vs* extremely likely/likely to accept COVID–19 vaccination  (n=228) | |
| --- | --- | --- | --- |
|  |  | OR (95% CI) | p–value |
| Demographics |  |  |  |
| ***Age group*** |  |  |  |
| 18–29 | 413 (19.0) | 0.40 (0.23–0.69) | 0.001 |
| 30–39 | 590 (27.1) | 0.58 (0.37–0.91) | 0.577 |
| 40–49 | 506 (23.3) | 0.40 (0.24–0.66) | p<0.001 |
| 50–59 | 324 (14.9) | 0.72 (0.44–1.17) | 0.183 |
| 60 and above | 342 (15.7) | 1 (ref) |  |
| ***Gender*** |  |  |  |
| Male | 814 (37.4) | 1.18 (0.85–1.64) | 0.311 |
| Female | 1361 (62.6) | 1 (ref) |  |
| ***Highest education level*** |  |  |  |
| Secondary school and below | 356 (16.4) | 0.40 (0.22–0.72) | 0.002 |
| Certificate/A–Level/Diploma | 511 (23.5) | 1.15 (0.71–1.87) | 0.569 |
| Bachelor degree | 883 (40.6) | 1.16 (0.75–1.81) | 0.502 |
| Postgraduate degree | 425 (19.5) | 1 (ref) |  |
| ***Ever delayed acceptance or refuse vaccine despite availability of vaccine service*** |  |  |  |
| Yes | 189 (8.7) | 4.21 (2.86–6.18) | p<0.001 |
| No | 1986 (91.3) | 1 (ref) |  |
| Vaccine characteristics influencing vaccination acceptance |  |  |  |
| ***Required doses of COVID–19 vaccine*** |  |  |  |
| Only accept single dose | 627 (28.8) | 3.38 (2.42–4.73) | p<0.001 |
| Do not mind | 1548 (71.2) | 1 (ref) |  |
| ***Effectiveness threshold of COVID–19 vaccine*** |  |  |  |
| Only accept 90% threshold | 1726 (79.4) | 1.39 (0.81–2.38) | 0.237 |
| Do not mind | 449 (20.6) | 1 (ref) |  |
| ***Adverse reaction of COVID–19 vaccine*** |  |  |  |
| Only accept minor adverse reactions | 1743 (80.1) | 1.19 (0.66–2.14) | 0.565 |
| Do not mind moderate adverse reactions | 432 (19.1) | 1 (ref) |  |
| ***Duration of COVID–19 vaccine protection*** |  |  |  |
| Only accept lesser than 12 months | 1503 (69.1) | 1.92 (1.16–3.17) | 0.011 |
| Do not mind | 672 (30.9) | 1 (ref) |  |
| ***Technology used in COVID–19 vaccine*** |  |  |  |
| Do not accept mRNA technology | 313 (14.4) | 3.47 (2.45–4.91) | p<0.001 |
| Do not mind | 511 (23.5) | 0.46 (0.26–0.83) | 0.010 |
| Do not know mRNA technology | 1351 (62.1) | 1 (ref) |  |
| ***Producing country of COVID–19 vaccine*** |  |  |  |
| Only accept a vaccine that is produced by specific countries | 1151 (52.9) | 1.20 (0.85–1.69) | 0.292 |
| Producing countries of a COVID–19 vaccine is not of my concern in vaccine acceptance | 1024 (47.1) | 1 (ref) |  |

Hosmer–Lemeshow test, chi–square: 12.103, p–value: 0.147; Nagelkerke R^2^ : 0.307

| Norway | All participants  n=(1382) | Extremely unlikely/ unlikely *vs* extremely likely/likely to accept COVID–19 vaccination  (n=64) | |
| --- | --- | --- | --- |
|  |  | OR (95% CI) | p–value |
| Demographics |  |  |  |
| ***Age group*** |  |  |  |
| 18–29 | 303 (21.9) | 0.44 (0.15–1.27) | 0.127 |
| 30–39 | 390 (28.2) | 0.93 (0.33–2.56) | 0.881 |
| 40–49 | 295 (21.3) | 0.62 (0.22–1.77) | 0.372 |
| 50–59 | 232 (16.8) | 0.62 (0.23–1.71) | 0.357 |
| 60 and above | 162 (11.7) | 1 (ref) |  |
| ***Gender*** |  |  |  |
| Male | 758 (54.8) | 1.32 (0.66–2.65) | 0.432 |
| Female | 624 (45.2) | 1 (ref) |  |
| ***Highest education level*** |  |  |  |
| Secondary school and below | 55 (4.0) | 5.68 (1.99–16.21) | 0.001 |
| Certificate/A–Level/Diploma | 471 (34.1) | 0.62 (0.25–1.56) | 0.313 |
| Bachelor degree | 489 (35.4) | 0.80 (0.35–1.80) | 0.583 |
| Postgraduate degree | 367 (26.6) | 1 (ref) |  |
| ***Ever delayed acceptance or refuse vaccine despite availability of vaccine service*** |  |  |  |
| Yes | 141 (10.2) | 7.33 (3.55–15.12) | p<0.001 |
| No | 1241 (89.8) | 1 (ref) |  |
| Vaccine characteristics influencing vaccination acceptance |  |  |  |
| ***Required doses of COVID–19 vaccine*** |  |  |  |
| Only accept single dose | 424 (30.7) | 2.52 (1.27–4.99) | 0.008 |
| Do not mind | 958 (69.3) | 1 (ref) |  |
| ***Effectiveness threshold of COVID–19 vaccine*** |  |  |  |
| Only accept 90% threshold | 811 (58.7) | 2.76 (1.22–6.23) | 0.015 |
| Do not mind | 571 (41.3) | 1 (ref) |  |
| ***Adverse reactions of COVID–19 vaccine*** |  |  |  |
| Only accept minor adverse reactions | 685 (49.6) | 1.55 (0.71–3.36) | 0.273 |
| Do not mind moderate adverse reactions | 697 (50.4) | 1 (ref) |  |
| ***Duration of COVID–19 vaccine protection*** |  |  |  |
| Only accept lesser than 12 months | 676 (48.9) | 2.68 (1.30–5.52) | 0.007 |
| Do not mind | 706 (51.1) | 1 (ref) |  |
| ***Technology used in COVID–19 vaccine*** |  |  |  |
| Do not accept mRNA technology | 202 (14.6) | 2.05 (0.93–4.52) | 0.076 |
| Do not mind | 585 (42.3) | 0.61 (0.25–1.46) | 0.010 |
| Do not know mRNA technology | 595 (43.1) | 1 (ref) |  |
| ***Producing country of COVID–19 vaccine*** |  |  |  |
| Only accept a vaccine that is produced by specific countries | 1106 (80.0) | 2.63 (1.19–5.84) | 0.018 |
| Producing countries of a COVID–19 vaccine is not of my concern in vaccine acceptance | 276 (20.0) | 1 (ref) |  |

Hosmer–Lemeshow test, chi–square: 19.296, p–value: 0.013; Nagelkerke R^2^ : 0.403

| Pakistan | All participants  (n=1271) | Extremely unlikely/ unlikely *vs* extremely likely/likely to accept COVID–19 vaccination  (n=272) | |
| --- | --- | --- | --- |
|  |  | OR (95% CI) | p–value |
| Demographics |  |  |  |
| ***Age group*** |  |  |  |
| 18–29 | 460 (36.2) | 0.32 (0.18–0.58) | p<0.001 |
| 30–39 | 279 (22.0) | 0.22 (0.12–0.43) | p<0.001 |
| 40–49 | 152 (12.0) | 0.54 (0.26–1.09) | 0.086 |
| 50–59 | 114 (9.0) | 0.78 (0.43–1.42) | 0.416 |
| 60 and above | 266 (20.9) | 1 (ref) |  |
| ***Gender*** |  |  |  |
| Male | 652 (51.3) | 0.76 (0.54–1.06) | 0.105 |
| Female | 619 (48.7) | 1 (ref) |  |
| ***Highest education level*** |  |  |  |
| Secondary school and below | 353 (27.8) | 2.09 (1.20–3.65) | 0.010 |
| Certificate/A–Level/Diploma | 93 (7.3) | 1.14 (0.57–2.27) | 0.712 |
| Bachelor degree | 402 (31.6) | 0.96 (0.59–1.55) | 0.851 |
| Postgraduate degree | 423 (33.3) | 1 (ref) |  |
| ***Ever delayed acceptance or refuse vaccine despite availability of vaccine service*** |  |  |  |
| Yes | 415 (32.7) | 4.29 (3.03–6.08) | p<0.001 |
| No | 856 (67.3) | 1 (ref) |  |
| Vaccine characteristics influencing vaccination acceptance |  |  |  |
| ***Required doses of COVID–19 vaccine*** |  |  |  |
| Only accept single dose | 645 (50.7) | 2.54 (1.62–3.97) | p<0.001 |
| Do not mind | 626 (49.3) | 1 (ref) |  |
| ***Effectiveness threshold of COVID–19 vaccine*** |  |  |  |
| Only accept 90% threshold | 846 (66.6) | 1.61 (1.02–2.54) | 0.041 |
| Do not mind | 425 (33.4) | 1 (ref) |  |
| ***Adverse reactions of COVID–19 vaccine*** |  |  |  |
| Only accept minor adverse reactions | 928 (73.0) | 0.77 (0.47–1.27) | 0.306 |
| Do not mind moderate adverse reactions | 343 (27.0) | 1 (ref) |  |
| ***Duration of COVID–19 vaccine protection*** |  |  |  |
| Only accept lesser than 12 months | 675 (53.1) | 2.87 (1.92–4.30) | p<0.001 |
| Do not mind | 596 (46.9) | 1 (ref) |  |
| ***Technology used in COVID–19 vaccine*** |  |  |  |
| Do not accept mRNA technology | 222 (17.5) | 1.02 (0.65–1.61) | 0.931 |
| Do not mind | 317 (24.9) | 0.98 (0.63–1.52) | 0.915 |
| Do not know mRNA technology | 732 (57.6) | 1 (ref) |  |
| ***Producing country of COVID–19 vaccine*** |  |  |  |
| Only accept a vaccine that is produced by specific countries | 736 (57.9) | 1.19 (0.82–1.72) | 0.369 |
| Producing countries of a COVID–19 vaccine is not of my concern in vaccine acceptance | 535 (42.1) | 1 (ref) |  |

Hosmer–Lemeshow test, chi–square: 11.353, p–value: 0.183; Nagelkerke R^2^ : 0.419

| Singapore | All participants (n=841) | Extremely unlikely/ unlikely *vs* extremely likely/likely to accept COVID–19 vaccination  (n=96) | |
| --- | --- | --- | --- |
|  |  | OR (95% CI) | p–value |
| Demographics |  |  |  |
| ***Age group*** |  |  |  |
| 18–29 | 73 (8.7) | 1.16 (0.34–3.92) | 0.816 |
| 30–39 | 231 (27.5) | 1.05 (0.42–2.61) | 0.919 |
| 40–49 | 211 (25.1) | 1.32 (0.55–3.17) | 0.532 |
| 50–59 | 185 (22.0) | 1.21 (0.50–2.61) | 0.675 |
| 60 and above | 141 (16.8) | 1 (ref) |  |
| ***Gender*** |  |  |  |
| Male | 343 (40.8) | 1.41 (0.82–2.41) | 0.219 |
| Female | 498 (59.2) | 1 (ref) |  |
| ***Highest education level*** |  |  |  |
| Secondary school and below | 84 (10.0) | 0.31 (0.10–0.96) | 0.042 |
| Certificate/A–Level/Diploma | 150 (17.8) | 0.47 (0.21–1.04) | 0.064 |
| Bachelor degree | 413 (49.1) | 0.58 (0.30–1.13) | 0.109 |
| Postgraduate degree | 194 (23.1) | 1 (ref) |  |
| ***Ever delayed acceptance or refuse vaccine despite availability of vaccine service*** |  |  |  |
| Yes | 112 (13.3) | 4.03 (2.24–7.25) | p<0.001 |
| No | 729 (86.7) | 1 (ref) |  |
| Vaccine characteristics influencing vaccination acceptance |  |  |  |
| ***Required doses of COVID–19 vaccine*** |  |  |  |
| Only accept single dose | 109 (13.0) | 6.77 (3.86–11.89) | p<0.001 |
| Do not mind | 732 (87.0) | 1 (ref) |  |
| ***Effectiveness threshold of COVID–19 vaccine*** |  |  |  |
| Only accept 90% threshold | 641 (76.2) | 3.01 (1.20–7.60) | 0.019 |
| Do not mind | 200 (23.8) | 1 (ref) |  |
| ***Adverse reactions of COVID–19 vaccine*** |  |  |  |
| Only accept minor adverse reactions | 610 (72.5) | 2.39 (0.89–6.43) | 0.084 |
| Do not mind moderate adverse reactions | 231 (27.5) | 1 (ref) |  |
| ***Duration of COVID–19 vaccine protection*** |  |  |  |
| Only accept lesser than 12 months | 566 (67.3) | 1.95 (0.92–4.13) | 0.083 |
| Do not mind | 275 (32.7) | 1 (ref) |  |
| ***Technology used in COVID–19 vaccine*** |  |  |  |
| Do not accept mRNA technology | 51 (6.1) | 2.23 (1.02–4.88) | 0.044 |
| Do not mind | 266 (31.6) | 0.21 (0.08–0.54) | 0.001 |
| Do not know mRNA technology | 524 (62.3) | 1 (ref) |  |
| ***Producing country of COVID–19 vaccine*** |  |  |  |
| Only accept a vaccine that is produced by specific countries | 535 (63.6) | 1.44 (0.80–2.59) | 0.227 |
| Producing countries of a COVID–19 vaccine is not of my concern in vaccine acceptance | 306 (36.4) | 1 (ref) |  |

Hosmer–Lemeshow test, chi–square: 10.315, p–value: 0.244; Nagelkerke R^2^ : 0.366

| Somalia | All participants  (n=894) | Extremely unlikely/ unlikely *vs* extremely likely/likely to accept COVID–19 vaccination  (n=121) | |
| --- | --- | --- | --- |
|  |  | OR (95% CI) | p–value |
| Demographics |  |  |  |
| ***Age group*** |  |  |  |
| 18–29 | 178 (19.9) | 0.40 (0.16–0.98) | 0.045 |
| 30–39 | 266 (29.8) | 0.60 (0.26–1.42) | 0.249 |
| 40–49 | 243 (27.2) | 0.37 (0.15–0.92) | 0.033 |
| 50–59 | 163 (18.2) | 0.28 (0.11–0.75) | 0.012 |
| 60 and above | 44 (4.9) | 1 (ref) |  |
| ***Gender*** |  |  |  |
| Male | 442 (49.4) | 0.86 (0.56–1.30) | 0.469 |
| Female | 452 (50.6) | 1 (ref) |  |
| ***Highest education level*** |  |  |  |
| Secondary school and below | 239 (26.7) | 4.57 (1.81–11.56) | 0.001 |
| Certificate/A–Level/Diploma | 283 (31.7) | 3.06 (1.20–7.85) | 0.020 |
| Bachelor degree | 294 (32.9) | 1.26 (0.47–3.33) | 0.647 |
| Postgraduate degree | 78 (8.7) | 1 (ref) |  |
| ***Ever delayed acceptance or refuse vaccine despite availability of vaccine service*** |  |  |  |
| Yes | 348 (38.9) | 2.15 (1.40–3.29) | p<0.001 |
| No | 546 (61.1) | 1 (ref) |  |
| Vaccine characteristics influencing vaccination acceptance |  |  |  |
| ***Required doses of COVID–19 vaccine*** |  |  |  |
| Only accept single dose | 548 (61.3) | 0.99 (0.64–1.53) | 0.950 |
| Do not mind | 346 (38.7) | 1 (ref) |  |
| ***Effectiveness threshold of COVID–19 vaccine*** |  |  |  |
| Only accept 90% threshold | 529 (59.2) | 1.19 (0.75–1.89) | 0.463 |
| Do not mind | 365 (40.8) | 1 (ref) |  |
| ***Adverse reactions of COVID–19 vaccine*** |  |  |  |
| Only accept minor adverse reactions | 620 (69.4) | 1.44 (0.90–2.32) | 0.130 |
| Do not mind moderate adverse reactions | 274 (30.6) | 1 (ref) |  |
| ***Duration of COVID–19 vaccine protection*** |  |  |  |
| Only accept lesser than 12 months | 508 (56.8) | 1.74 (1.10–2.75) | 0.017 |
| Do not mind | 386 (43.2) | 1 (ref) |  |
| ***Technology used in COVID–19 vaccine*** |  |  |  |
| Do not accept mRNA technology | 433 (48.4) | 0.36 (0.22–0.59) | p<0.001 |
| Do not mind | 289 (32.3) | 0.24 (0.14–0.43) | p<0.001 |
| Do not know mRNA technology | 172 (19.2) | 1 (ref) |  |
| ***Producing country of COVID–19 vaccine*** |  |  |  |
| Only accept a vaccine that is produced by specific countries | 454 (50.8) | 1.43 (0.93–2.21) | 0.105 |
| Producing countries of a COVID–19 vaccine is not of my concern in vaccine acceptance | 440 (49.2) | 1 (ref) |  |

Hosmer–Lemeshow test, chi–square: 3.441, p–value: 0.904; Nagelkerke R^2^ : 0.204

| South Africa | All participants  (n=1086) | Extremely unlikely/ unlikely *vs* extremely likely/likely to accept COVID–19 vaccination  (n=93) | |
| --- | --- | --- | --- |
|  |  | OR (95% CI) | p–value |
| Demographics |  |  |  |
| ***Age group*** |  |  |  |
| 18–29 | 194 (17.9) | 4.21 (1.58–11.22) | 0.004 |
| 30–39 | 267 (24.6) | 1.62 (0.57–4.60) | 0.363 |
| 40–49 | 254 (23.4) | 1.75 (0.62–4.95) | 0.296 |
| 50–59 | 186 (17.1) | 2.16 (0.76–6.17) | 0.150 |
| 60 and above | 185 (17.0) | 1 (ref) |  |
| ***Gender*** |  |  |  |
| Male | 432 (39.8) | 0.90 (0.49–1.64) | 0.731 |
| Female | 654 (60.2) | 1 (ref) |  |
| ***Highest education level*** |  |  |  |
| Secondary school and below | 168 (15.5) | 0.39 (0.15–1.03) | 0.058 |
| Certificate/A–Level/Diploma | 405 (37.3) | 0.33 (0.15–0.70) | 0.004 |
| Bachelor degree | 224 (20.6) | 0.65 (0.29–1.44) | 0.290 |
| Postgraduate degree | 289 (26.6) | 1 (ref) |  |
| ***Ever delayed acceptance or refuse vaccine despite availability of vaccine service*** |  |  |  |
| Yes | 79 (7.3) | 6.54 (3.20–13.37) | p<0.001 |
| No | 1007 (92.7) | 1 (ref) |  |
| Vaccine characteristics influencing vaccination acceptance |  |  |  |
| ***Required doses of COVID–19 vaccine*** |  |  |  |
| Only accept single dose | 298 (27.4) | 4.87 (2.55–9.33) | p<0.001 |
| Do not mind | 788 (72.6) | 1 (ref) |  |
| ***Effectiveness threshold of COVID–19 vaccine*** |  |  |  |
| Only accept 90% threshold | 434 (40.0) | 2.10 (0.95–4.64) | 0.067 |
| Do not mind | 652 (60.0) | 1 (ref) |  |
| ***Adverse reactions of COVID–19 vaccine*** |  |  |  |
| Only accept minor adverse reactions | 456 (42.0) | 3.96 (1.56–10.05) | 0004 |
| Do not mind moderate adverse reactions | 630 (58.0) | 1 (ref) |  |
| ***Duration of COVID–19 vaccine protection*** |  |  |  |
| Only accept lesser than 12 months | 457 (42.1) | 2.59 (1.15–5.84) | 0.022 |
| Do not mind | 629 (57.9) | 1 (ref) |  |
| ***Technology used in COVID–19 vaccine*** |  |  |  |
| Do not accept mRNA technology | 106 (9.8) | 2.76 (1.36–5.62) | 0.005 |
| Do not mind | 382 (35.2) | 0.53 (0.24–1.21) | 0.132 |
| Do not know mRNA technology | 598 (55.1) | 1 (ref) |  |
| ***Producing country of COVID–19 vaccine*** |  |  |  |
| Only accept a vaccine that is produced by specific countries | 317 (29.2) | 1.75 (0.95–3.21) | 0.073 |
| Producing countries of a COVID–19 vaccine is not of my concern in vaccine acceptance | 769 (70.8) | 1 (ref) |  |

Hosmer–Lemeshow test, chi–square: 6.600, p–value: 0.580; Nagelkerke R^2^ : 0.534

| Sri Lanka | All participants  (n=776) | Extremely unlikely/ unlikely *vs* extremely likely/likely to accept COVID–19 vaccination  (n=59) | |
| --- | --- | --- | --- |
|  |  | OR (95% CI) | p–value |
| Demographics |  |  |  |
| ***Age group*** |  |  |  |
| 18–29 | 260 (33.5) | 3.87 (0.46–32.18) | 0.211 |
| 30–39 | 115 (14.8) | 2.28 (0.26–20.13) | 0.459 |
| 40–49 | 208 (26.8) | 0.39 (0.04–4.02) | 0.427 |
| 50–59 | 167 (21.5) | 1.32 (0.15–11.76) | 0.801 |
| 60 and above | 26 (3.4) | 1 (ref) |  |
| ***Gender*** |  |  |  |
| Male | 199 (25.6) | 1.33 (0.72–2.46) | 0.360 |
| Female | 577 (74.4) | 1 (ref) |  |
| ***Highest education level*** |  |  |  |
| Secondary school and below | 16 (2.1) | – | – |
| Certificate/A–Level/Diploma | 202 (26.0) | 0.80 (0.28–2.78) | 0.682 |
| Bachelor degree | 462 (59.5) | 1.06 (0.42–2.68) | 0.898 |
| Postgraduate degree | 96 (12.4) | 1 (ref) |  |
| ***Ever delayed acceptance or refuse vaccine despite availability of vaccine service*** |  |  |  |
| Yes | 62 (8.0) | 3.60 (1.74–7.49) | 0.001 |
| No | 714 (92.0) | 1 (ref) |  |
| Vaccine characteristics influencing vaccination acceptance |  |  |  |
| ***Required doses of COVID–19 vaccine*** |  |  |  |
| Only accept single dose | 472 (60.8) | 2.06 (1.08–3.94) | 0.028 |
| Do not mind | 304 (39.2) | 1 (ref) |  |
| ***Effectiveness threshold of COVID–19 vaccine*** |  |  |  |
| Only accept 90% threshold | 460 (59.3) | 1.97 (0.90–4.44) | 0.091 |
| Do not mind | 316 (40.7) | 1 (ref) |  |
| ***Adverse reactions of COVID–19 vaccine*** |  |  |  |
| Only accept minor adverse reactions | 621 (80.0) | 1.00 (0.47–2.14) | 0.991 |
| Do not mind moderate adverse reactions | 155 (20.0) | 1 (ref) |  |
| ***Duration of COVID–19 vaccine protection*** |  |  |  |
| Only accept lesser than 12 months | 523 (67.4) | 0.84 (0.41–1.74) | 0.640 |
| Do not mind | 253 (32.6) | 1 (ref) |  |
| ***Technology used in COVID–19 vaccine*** |  |  |  |
| Do not accept mRNA technology | 358 (46.1) | 0.70 (0.31–1.56) | 0.382 |
| Do not mind | 159 (20.5) | 0.76 (0.34–1.71) | 0.504 |
| Do not know mRNA technology | 259 (33.4) | 1 (ref) |  |
| ***Producing country of COVID–19 vaccine*** |  |  |  |
| Only accept a vaccine that is produced by specific countries | 509 (65.6) | 1.25 (0.64–2.45) | 0.512 |
| Producing countries of a COVID–19 vaccine is not of my concern in vaccine acceptance | 267 (34.4) | 1 (ref) |  |

Hosmer–Lemeshow test, chi–square: 4.843, p–value: 0..774; Nagelkerke R^2^ : 0.182

Highest educational level of secondary school and below was excluded due to small sample size

| United Arab Emirates | All participants  n=(938) | Extremely unlikely/ unlikely *vs* extremely likely/likely to accept COVID–19 vaccination  (n=148) | |
| --- | --- | --- | --- |
|  |  | OR (95% CI) | p–value |
| Demographics |  |  |  |
| ***Age group*** |  |  |  |
| 18–29 | 170 (18.1) | 1.45 (0.64–3.33) | 0.376 |
| 30–39 | 293 (31.2) | 1.76 (0.81–3.85) | 0.155 |
| 40–49 | 268 (28.6) | 1.83 (0.82–4.07) | 0.138 |
| 50–59 | 130 (13.9) | 1.55 (0.64–3.74) | 0.331 |
| 60 and above | 77 (8.2) | 1 (ref) |  |
| ***Gender*** |  |  |  |
| Male | 459 (48.9) | 1.26 (0.86–1.83) | 0.234 |
| Female | 479 (51.1) | 1 (ref) |  |
| ***Highest education level*** |  |  |  |
| Secondary school and below | 240 (25.6) | 2.21 (0.81–6.07) | 0.122 |
| Certificate/A–Level/Diploma | 232 (24.7) | 2.37 (0.87–6.49) | 0.093 |
| Bachelor degree | 376 (40.1) | 2.52 (0.94–6.77) | 0.067 |
| Postgraduate degree | 90 (9.6) | 1 (ref) |  |
| ***Ever delayed acceptance or refuse vaccine despite availability of vaccine service*** |  |  |  |
| Yes | 355 (37.8) | 1.51 (1.03–2.21) | 0.034 |
| No | 583 (62.2) | 1 (ref) |  |
| Vaccine characteristics influencing vaccination acceptance |  |  |  |
| ***Required doses of COVID–19 vaccine*** |  |  |  |
| Only accept single dose | 366 (39.0) | 1.44 (0.97–2.14) | 0.073 |
| Do not mind | 572 (61.0) | 1 (ref) |  |
| ***Effectiveness threshold of COVID–19 vaccine*** |  |  |  |
| Only accept 90% threshold | 487 (51.9) | 2.27 (1.46–3.52) | p<0.001 |
| Do not mind | 451 (48.1) | 1 (ref) |  |
| ***Adverse reactions of COVID–19 vaccine*** |  |  |  |
| Only accept minor adverse reactions | 394 (74.0) | 1.31 (0.79–2.17) | 0.300 |
| Do not mind moderate adverse reactions | 244 (26.0) | 1 (ref) |  |
| ***Duration of COVID–19 vaccine protection*** |  |  |  |
| Only accept lesser than 12 months | 477 (50.9) | 1.72 (1.15–2.56) | 0.008 |
| Do not mind | 461 (49.1) | 1 (ref) |  |
| ***Technology used in COVID–19 vaccine*** |  |  |  |
| Do not accept mRNA technology | 284 (30.3) | 0.54 (0.34–0.84) | 0.007 |
| Do not mind | 350 (37.3) | 0.49 (0.31–0.76) | 0.002 |
| Do not know mRNA technology | 304 (32.4) | 1 (ref) |  |
| ***Producing country of COVID–19 vaccine*** |  |  |  |
| Only accept a vaccine that is produced by specific countries | 461 (49.1) | 1.81 (1.24–2.66) | 0.002 |
| Producing countries of a COVID–19 vaccine is not of my concern in vaccine acceptance | 477 (50.9) | 1 (ref) |  |

Hosmer–Lemeshow test, chi–square: 11.574, p–value: 0.171; Nagelkerke R^2^ : 0.150

| United Kingdom | All participants  n=(1021) | Extremely unlikely/ unlikely *vs* extremely likely/likely to accept COVID–19 vaccination  (n=85) | |
| --- | --- | --- | --- |
|  |  | OR (95% CI) | p–value |
| Demographics |  |  |  |
| ***Age group*** |  |  |  |
| 18–29 | 244 (23.9) | 0.18 (0.61–0.55) | 0.003 |
| 30–39 | 399 (39.1) | 0.45 (0.17–1.19) | 0.108 |
| 40–49 | 182 (17.8) | 0.44 (0.15–1.28) | 0.130 |
| 50–59 | 148 (14.5) | 1.48 (0.55–4.03) | 0.441 |
| 60 and above | 48 (4.7) | 1 (ref) |  |
| ***Gender*** |  |  |  |
| Male | 564 (55.2) | 1.75 (0.89–3.42) | 0.994 |
| Female | 457 (44.8) | 1 (ref) |  |
| ***Highest education level*** |  |  |  |
| Secondary school and below | 7 (0.7) | – | – |
| Certificate/A–Level/Diploma | 298 (29.2) | 1.75 (0.89–3.42) | 0.103 |
| Bachelor degree | 342 (33.5) | 1.92 (0.99–3.75) | 0.056 |
| Postgraduate degree | 374 (36.6) | 1 (ref) |  |
| ***Ever delayed acceptance or refuse vaccine despite availability of vaccine service*** |  |  |  |
| Yes | 87 (8.5) | 2.75 (1.21–6.25) | 0.016 |
| No | 934 (91.5) | 1 (ref) |  |
| Vaccine characteristics influencing vaccination acceptance |  |  |  |
| ***Required doses of COVID–19 vaccine*** |  |  |  |
| Only accept single dose | 362 (35.5) | 2.29 (1.36–3.84) | 0.002 |
| Do not mind | 659 (64.5) | 1 (ref) |  |
| ***Effectiveness threshold of COVID–19 vaccine*** |  |  |  |
| Only accept 90% threshold | 582 (57.0) | 5.55 (2.58–11.94) | p<0.001 |
| Do not mind | 439 (43.0) | 1 (ref) |  |
| ***Adverse reactions of COVID–19 vaccine*** |  |  |  |
| Only accept minor adverse reactions | 864 (84.6) | 0.96 (0.41–2.22) | 0.922 |
| Do not mind moderate adverse reactions | 157 (15.4) | 1 (ref) |  |
| ***Duration of COVID–19 vaccine protection*** |  |  |  |
| Only accept lesser than 12 months | 520 (50.9) | 2.43 (1.31–4.51) | 0.005 |
| Do not mind | 501 (49.1) | 1 (ref) |  |
| ***Technology used in COVID–19 vaccine*** |  |  |  |
| Do not accept mRNA technology | 101 (9.9) | 1.76 (0.76–4.06) | 0.185 |
| Do not mind | 385 (37.7) | 0.52 (0.27–0.99) | 0.047 |
| Do not know mRNA technology | 535 (52.4) | 1 (ref) |  |
| ***Producing country of COVID–19 vaccine*** |  |  |  |
| Only accept a vaccine that is produced by specific countries | 771 (75.5) | 1.27 (0.61–2.68) | 0.523 |
| Producing countries of a COVID–19 vaccine is not of my concern in vaccine acceptance | 250 (24.5) | 1 (ref) |  |

Hosmer–Lemeshow test, chi–square: 16.292, p–value: 0.038; Nagelkerke R^2^ : 0.278

Highest educational level of secondary school and below was excluded due to small sample size

| United States of America | All participants  n=(968) | Extremely unlikely/ unlikely *vs* extremely likely/likely to accept COVID–19 vaccination  (n=285) | |
| --- | --- | --- | --- |
|  |  | OR (95% CI) | p–value |
| Demographics |  |  |  |
| ***Age group*** |  |  |  |
| 18–29 | 158 (16.3) | 0.33 (0.19–0.60) | p<0.001 |
| 30–39 | 323 (33.4) | 0.28 (0.17–0.48) | p<0.001 |
| 40–49 | 250 (25.8) | 0.33 (0.19–0.57) | p<0.001 |
| 50–59 | 121 (12.5) | 0.54 (0.30–0.99) | 0.045 |
| 60 and above | 116 (12.0) | 1 (ref) |  |
| ***Gender*** |  |  |  |
| Male | 471 (48.7) | 1.89 (1.34–2.66) | p<0.001 |
| Female | 485 (50.1) | 1 (ref) |  |
| Other | 12 (1.2) | – | – |
| ***Highest education level*** |  |  |  |
| Secondary school and below | 115 (11.9) | 1.61 (0.84–3.10) | 0.152 |
| Certificate/A–Level/Diploma | 317 (32.7) | 0.71 (0.41–1.24) | 0.228 |
| Bachelor degree | 441 (45.6) | 0.67 (0.39–1.15) | 0.147 |
| Postgraduate degree | 95 (9.8) | 1 (ref) |  |
| ***Ever delayed acceptance or refuse vaccine despite availability of vaccine service*** |  |  |  |
| Yes | 140 (14.5) | 0.62 (0.39–0.98) | 0.039 |
| No | 828 (85.5) | 1 (ref) |  |
| Vaccine characteristics influencing vaccination acceptance |  |  |  |
| ***Required doses of COVID–19 vaccine*** |  |  |  |
| Only accept single dose | 478 (49.4) | 0.56 (0.39–0.80) | 0.001 |
| Do not mind | 490 (50.6) | 1 (ref) |  |
| ***Effectiveness threshold of COVID–19 vaccine*** |  |  |  |
| Only accept 90% threshold | 487 (50.3) | 2.43 (1.67–3.53) | p<0.001 |
| Do not mind | 481 (49.7) | 1 (ref) |  |
| ***Adverse reactions of COVID–19 vaccine*** |  |  |  |
| Only accept minor adverse reactions | 497 (51.3) | 1.67 (1.17–2.40) | 0.005 |
| Do not mind moderate adverse reactions | 471 (48.7) | 1 (ref) |  |
| ***Duration of COVID–19 vaccine protection*** |  |  |  |
| Only accept lesser than 12 months | 679 (70.1) | 0.55 (0.38–0.80) | 0.001 |
| Do not mind | 289 (29.9) | 1 (ref) |  |
| ***Technology used in COVID–19 vaccine*** |  |  |  |
| Do not accept mRNA technology | 189 (19.2) | 0.70 (0.44–1.11) | 0.127 |
| Do not mind | 488 (50.4) | 0.56 (0.39–0.82) | 0.003 |
| Do not know mRNA technology | 294 (30.4) | 1 (ref) |  |
| ***Producing country of COVID–19 vaccine*** |  |  |  |
| Only accept a vaccine that is produced by specific countries | 727 (75.1) | 0.29 (0.20–0.42) | p<0.001 |
| Producing countries of a COVID–19 vaccine is not of my concern in vaccine acceptance | 241 (24.9) | 1 (ref) |  |

Hosmer–Lemeshow test, chi–square: 10.800, p–value: 0.000; Nagelkerke R^2^ : 0.322

Other gender was excluded due to small sample size

| Vietnam | All participants  n=(1462) | Extremely unlikely/ unlikely *vs* extremely likely/likely to accept COVID–19 vaccination  (n=170) | |
| --- | --- | --- | --- |
|  |  | OR (95% CI) | p–value |
| Demographics |  |  |  |
| ***Age group*** |  |  |  |
| 18–29 | 467 (31.9) | 0.32 (0.15–0.69) | 0.004 |
| 30–39 | 543 (37.1) | 0.31 (0.16–0.59) | p<0.001 |
| 40–49 | 231 (15.8) | 0.41 (0.20–0.82) | 0.012 |
| 50–59 | 144 (9.8) | 0.56 (0.28–1.12) | 0.100 |
| 60 and above | 77 (5.3) | 1 (ref) |  |
| ***Gender*** |  |  |  |
| Male | 757 (51.8) | 0.75 (0.52–1.08) | 0.119 |
| Female | 705 (48.2) | 1 (ref) |  |
| ***Highest education level*** |  |  |  |
| Secondary school and below | 269 (18.4) | 7.65 (3.61–16.22) | p<0.001 |
| Certificate/A–Level/Diploma | 470 (32.1) | 1.59 (0.74–3.41) | 0.237 |
| Bachelor degree | 582 (39.8) | 0.77 (0.33–1.76) | 0.530 |
| Postgraduate degree | 141 (9.6) | 1 (ref) |  |
| ***Ever delayed acceptance or refuse vaccine despite availability of vaccine service*** |  |  |  |
| Yes | 110 (7.5) | 4.94 (2.36–10.33) | p<0.001 |
| No | 1352 (92.5) | 1 (ref) |  |
| Vaccine characteristics influencing vaccination acceptance |  |  |  |
| ***Required doses of COVID–19 vaccine*** |  |  |  |
| Only accept single dose | 887 (60.7) | 4.96 (2.94–8.37) | p<0.001 |
| Do not mind | 575 (39.3) | 1 (ref) |  |
| ***Effectiveness threshold of COVID–19 vaccine*** |  |  |  |
| Only accept 90% threshold | 1159 (79.3) | 1.58 (0.90–2.79) | 0.115 |
| Do not mind | 303 (20.7) | 1 (ref) |  |
| ***Adverse reactions of COVID–19 vaccine*** |  |  |  |
| Only accept minor adverse reactions | 1045 (71.5) | 2.12 (1.30–3.46) | 0.003 |
| Do not mind moderate adverse reactions | 417 (28.5) | 1 (ref) |  |
| ***Duration of COVID–19 vaccine protection*** |  |  |  |
| Only accept lesser than 12 months | 908 (62.1) | 1.31 (0.86–1.99) | 0.207 |
| Do not mind | 554 (37.9) | 1 (ref) |  |
| ***Technology used in COVID–19 vaccine*** |  |  |  |
| Do not accept mRNA technology | 467 (31.9) | 1.23 (0.82–1.83) | 0.324 |
| Do not mind | 322 (22.0) | 0.60 (0.35–1.05) | 0.073 |
| Do not know mRNA technology | 673 (46.0) | 1 (ref) |  |
| ***Producing country of COVID–19 vaccine*** |  |  |  |
| Only accept a vaccine that is produced by specific countries | 995 (68.1) | 1.14 (0.74–1.75) | 0.566 |
| Producing countries of a COVID–19 vaccine is not of my concern in vaccine acceptance | 467 (31.9) | 1 (ref) |  |

Hosmer–Lemeshow test, chi–square: 7.861, p–value: 0.447; Nagelkerke R^2^ : 0.30
