# Supplementary material for: COVID-19 vaccination intention and vaccine characteristics influencing vaccination acceptance: a global survey of 17 countries
Source: Infect Dis Poverty. 2021 Oct 7;10:122. doi: 10.1186/s40249-021-00900-w (PMC8496428; doi:10.1186/s40249-021-00900-w)
Supplement: Supplementary file 7 — Additional file 7. Vaccine characteristics influencing vaccination acceptance by demographics for individual country. [file 40249_2021_900_MOESM7_ESM.docx]

Vaccine characteristics influencing vaccination acceptance by demographics for individual country

| **Australia**  N=811 | ***Required doses of COVID-19 vaccine^a^*** | ***Effectiveness threshold of COVID-19 vaccine^b^*** | ***Adverse reactions of COVID-19 vaccine^c^*** | ***Duration of COVID-19 vaccine protection^d^*** | ***Technology used in COVID-19 vaccine^e^*** | ***Producing country of COVID-19 vaccine^f^*** |
| --- | --- | --- | --- | --- | --- | --- |
| Demographics |  |  |  |  |  |  |
| ***Age group*** |  |  |  |  |  |  |
| 18–29 | 2.27 (1.08–4.76)* | 0.56 (0.27–1.17) | 0.41 (0.19–0.89)* | 1.48 (0.62–3.57) | 0.53 (0.23–1.19) | 2.11 (0.85–5.26) |
| 30–39 | 2.86 (1.43–5.71)** | 0.47 (0.24–0.95)* | 0.50 (0.24–1.04) | 1.31 (0.59–2.92) | 0.69 (0.33–1.43) | 2.87 (1.25–6.59)* |
| 40–49 | 2.29 (1.16–4.51)* | 0.54 (0.27–1.07) | 0.49 (0.24–1.02) | 1.02 (0.48–2.31) | 0.90 (0.44–1.83) | 1.50 (0.69–3.23) |
| 50–59 | 2.06 (0.99–4.31) | 0.76 (0.36–1.59) | 0.48 (0.22–1.06) | 0.82 (0.35–1.88) | 1.09 (0.51–2.36) | 0.96 (0.42–2.17) |
| 60 and above | 1 (ref) | 1 (ref) | 1 (ref) | 1 (ref) | 1 (ref) | 1 (ref) |
| ***Gender*** |  |  |  |  |  |  |
| Male | 1.01 (0.76–1.35) | 1.04 (0.78–1.37) | 0.82 (0.62–1.10) | 1.27 (0.91–1.79) | 0.95 (0.69–1.31) | 1.50 (1.01–2.23)* |
| Female | 1 (ref) | 1 (ref) | 1 (ref) | 1 (ref) | 1 (ref) | 1 (ref) |
| ***Highest education level*** |  |  |  |  |  |  |
| Secondary school and below | 0.75 (0.34–1.67) | 1.66 (0.74–3.73) | 1.60 (0.69–.3.73) | 1.54 (0.56–4.24) | 0.52 (0.20–1.35) | 1.99 (0.73–5.39) |
| Certificate/A–Level/Diploma | 0.95 (0.57–1.58) | 1.37 (0.81–2.30) | 0.86 (0.51–1.46) | 1.10 (0.61–1.98) | 0.82 (0.47–1.43) | 2.90 (1.53–5.48)** |
| Bachelor degree | 1.47 (0.89–2.40) | 1.20 (0.73–1.98)*** | 0.98 (0.60–1.63) | 1.09 (0.62–1.92) | 0.96 (0.57–1.61) | 1.78 (1.02–3.12)* |
| Postgraduate degree | 1 (ref) | 1 (ref) | 1 (ref) | 1 (ref) | 1 (ref) | 1 (ref) |
| ***Ever delayed acceptance or refuse vaccine despite availability of vaccine service*** |  |  |  |  |  |  |
| Yes | 0.94 (0.59–1.50) | 2.27 (1.43–3.61)*** | 4.78 (2.82–8.10)*** | 1.03 (0.60–1.76) | 1.85 (1.16–2.95)* | 0.39 (0.23–0.65)*** |
| No | 1 (ref) | 1 (ref) | 1 (ref) | 1 (ref) | 1 (ref) | 1 (ref) |

^a^ *P*–value: 0.904; Nagelkerke *R^2^* : 0.037

^b^ *P* –value: 0.398; Nagelkerke *R^2^* : 0.040

^c^ *P* –value: 0.956; Nagelkerke *R^2^* : 0.091

^d^ *P* –value: 0.970; Nagelkerke *R^2^* : 0.015

^e^ *P* –value: 0.418; Nagelkerke *R^2^* : 0.035

^f^ *P* –value: 0.147; Nagelkerke *R^2^* : 0.116

| **Bangladesh**  N=1094 | ***Required doses of COVID-19 vaccine^a^*** | ***Effectiveness threshold of COVID-19 vaccine^b^*** | ***Adverse reactions of COVID-19 vaccine^c^*** | ***Duration of COVID-19 vaccine protection^d^*** | ***Technology used in COVID-19 vaccine^e^*** | ***Producing country of COVID-19 vaccine^f^*** |
| --- | --- | --- | --- | --- | --- | --- |
| Demographics |  |  |  |  |  |  |
| ***Age group*** |  |  |  |  |  |  |
| 18–29 | 5.61 (2.56–12.28)*** | 1.95 (0.99–3.85) | 0.80 (0.35–1.80) | 1.25 (0.64–2.44) | 4.45 (1.03–19.34)* | 1.41 (0.77–2.60) |
| 30–39 | 5.13 (2.38–11.08)*** | 2.47 (1.28–4.76)** | 0.82 (0.37–1.79) | 1.29 (0.68–2.47) | 5.05 (1.18–21.62)* | 2.12 (1.17–3.84)* |
| 40–49 | 4.48 (1.96–10.21)*** | 2.01 (0.95–4.27) | 0.76 (0.32–1.80) | 1.45 (0.69–3.03) | 3.56 (0.79–16.11) | 1.61 (0.83–3.11) |
| 50–59 | 2.49 (0.95–6.51) | 1.06 (0.454–2.49) | 0.80 (0.29–2.24) | 1.06 (0.45–2.52) | 5.18 (1.04–25.73)* | 1.44 (0.65–3.17) |
| 60 and above | 1 (ref) | 1 (ref) | 1 (ref) | 1 (ref) | 1 (ref) | 1 (ref) |
| ***Gender*** |  |  |  |  |  |  |
| Male | 0.99 (0.74–1.32) | 0.67 (0.45–0.98)* | 0.76 (0.52–1.08) | 1.16 (0.84–1.59) | 0.96 (0.66–1.40) | 1.02 (0.77–1.36) |
| Female | 1 (ref) | 1 (ref) | 1 (ref) | 1 (ref) | 1 (ref) | 1 (ref) |
| ***Highest education level*** |  |  |  |  |  |  |
| Secondary school and below | 1.65 (1.01–2.71)* | 1.27 (0.67–2.40) | 1.54 (0.82–2.87) | 2.40 (1.28–4.49)** | 0.52 (0.26–1.03) | 0.63 (0.40–1.00) |
| Certificate/A–Level/Diploma | 1.62 (0.95–2.75) | 2.69 (1.11–6.52)* | 2.01 (0.96–4.22) | 1.02 (0.59–1.76) | 0.57 (0.27–1.03) | 1.27 (0.76–2.13) |
| Bachelor degree | 0.91 (0.68–1.22) | 1.21 (0.83–1.76) | 1.61 (1.11–2.34)* | 1.37 (0.99–1.88) | 0.94 (0.65–1.36) | 0.98 (0.74–1.30) |
| Postgraduate degree | 1 (ref) | 1 (ref) | 1 (ref) | 1 (ref) | 1 (ref) | 1 (ref) |
| ***Ever delayed acceptance or refuse vaccine despite availability of vaccine service*** |  |  |  |  |  |  |
| Yes | 4.72 (3.51–6.33)*** | 2.28 (1.51–3.44)*** | 0.93 (0.66–1.31) | 2.11 (1.52–2.93) | 2.83 (2.03–3.95)*** | 2.24 (1.70–2.97)*** |
| No | 1 (ref) | 1 (ref) | 1 (ref) | 1 (ref) | 1 (ref) | 1 (ref) |

^a^ *P*–value: 0.273; Nagelkerke *R2* : 0.191

^b^ *P*–value: 0.629; Nagelkerke *R2* : 0.070

^c^ *P*–value: 0.120; Nagelkerke *R2* : 0.015

^d^ *P*–value: 0.965; Nagelkerke *R2* : 0.054

^e^ *P*–value: 0.662; Nagelkerke *R2* : 0.079

^f^ *P*–value: 0.629; Nagelkerke *R2* : 0.062

| **China**  N=1373 | ***Required doses of COVID-19 vaccine^a^*** | ***Effectiveness threshold of COVID-19 vaccine^b^*** | ***Adverse reactions of COVID-19 vaccine^c^*** | ***Duration of COVID-19 vaccine protection^d^*** | ***Technology used in COVID-19 vaccine^e^*** | ***Producing country of COVID-19 vaccine^f^*** |
| --- | --- | --- | --- | --- | --- | --- |
| Demographics |  |  |  |  |  |  |
| ***Age group*** |  |  |  |  |  |  |
| 18–29 | 0.24 (0.13–0.46)*** | 0.32 (0.14–0.70)** | 0.66 (0.25–1.74) | 0.27 (0.13–0.55)*** | 0.65 (0.25–1.64) | 0.34 (0.17–0.63)** |
| 30–39 | 0.34 (0.18–0.66)** | 0.36 (0.16–0.81)* | 0.52 (0.19–1.40) | 0.36 (0.17–0.76)** | 0.65 (0.25–1.72) | 0.28 (0.14–0.55)*** |
| 40–49 | 0.34 (0.16–0.70)** | 0.25 (0.11–0.58)** | 0.44 (0.16–1.22) | 0.34 (0.15–0.75)** | 0.61 (0.22–1.74) | 0.28 (0.13–0.58)** |
| 50–59 | 0.44 (0.20–0.94)* | 0.58 (0.23–1.46) | 0.57 (0.19–1.74) | 0.39 (0.17–0.91)* | 0.54 (0.16–1.81) | 0.47 (0.22–1.03) |
| 60 and above | 1 (ref) | 1 (ref) | 1 (ref) | 1 (ref) | 1 (ref) | 1 (ref) |
| ***Gender*** |  |  |  |  |  |  |
| Male | 1.06 (0.82–1.39) | 0.92 (0.74–1.15) | 0.71 (0.53–0.95)** | 0.83 (0.66–1.03) | 1.93 (1.34–2.76)*** | 1.11 (0.89–1.38) |
| Female | 1 (ref) | 1 (ref) | 1 (ref) | 1 (ref) | 1 (ref) | 1 (ref) |
| ***Highest education level*** |  |  |  |  |  |  |
| Secondary school and below | 2.60 (1.59–4.23)*** | 2.03 (1.34–3.09)** | 1.00 (0.57–1.76) | 1.85 (1.23–2.79)** | 0.64 (0.34–1.18) | 0.71 (0.47–1.06) |
| Certificate/A–Level/Diploma | 1.81 (1.14–2.86)* | 1.29 (0.90–1.86) | 0.67 (0.42–1.07) | 0.90 (0.63–1.29) | 0.50 (0.29–0.88)* | 0.73 (0.51–1.05) |
| Bachelor degree | 1.54 (1.04–2.27)* | 1.65 (1.23–2.20)** | 0.97 (0.65–1.45) | 1.21 (0.91–1.61) | 0.51 (0.33–0.78)** | 1.20 (0.90–1.60) |
| Postgraduate degree | 1 (ref) | 1 (ref) | 1 (ref) | 1 (ref) | 1 (ref) | 1 (ref) |
| ***Ever delayed acceptance or refuse vaccine despite availability of vaccine service*** |  |  |  |  |  |  |
| Yes | 2.86 (2.14–3.81)*** | 1.40 (1.07–1.84)** | 1.11 (0.78–1.59) | 1.22 (0.94–1.59) | 2.94 (2.03–4.24)*** | 1.46 (1.13–1.90)** |
| No | 1 (ref) | 1 (ref) | 1 (ref) | 1 (ref) | 1 (ref) | 1 (ref) |

^a^ *P*–value: 0.521; Nagelkerke *R^2^* : 0.104

^b^ *P*–value: 0.330; Nagelkerke *R^2^* : 0.040

^c^ *P*–value: 0.996; Nagelkerke *R^2^* : 0.021

^d^ *P*–value: 0.900; Nagelkerke *R^2^* : 0.039

^e^ *P*–value: 0.751; Nagelkerke *R^2^* : 0.081

^f^ *P*–value: 0.057; Nagelkerke *R^2^* : 0.033

| **India**  N=1566 | ***Required doses of COVID-19 vaccine^a^*** | ***Effectiveness threshold of COVID-19 vaccine^b^*** | ***Adverse reactions of COVID-19 vaccine^c^*** | ***Duration of COVID-19 vaccine protection^d^*** | ***Technology used in COVID-19 vaccine^e^*** | ***Producing country of COVID-19 vaccine^f^*** |
| --- | --- | --- | --- | --- | --- | --- |
| Demographics |  |  |  |  |  |  |
| ***Age group*** |  |  |  |  |  |  |
| 18–29 | 1.69 (1.09–2.62)* | 3.48 (2.25–5.38)*** | 6.74 (4.28–10.61)*** | 1.40 (0.93–2.11) | 0.58 (0.37–0.91)* | 1.22 (0.80–1.85) |
| 30–39 | 2.81 (1.79–4.43)*** | 0.72 (0.47–1.12) | 2.54 (1.63–3.95)*** | 0.62 (0.40–0.95)* | 1.65 (1.05–2.58)* | 0.81 (0.52–1.25) |
| 40–49 | 2.89 (1.84–4.53)*** | 0.69 (0.45–1.06) | 2.66 (1.72–4.13)*** | 0.74 (0.48–1.13) | 2.03 (1.31–3.16)** | 1.13 (0.73–1.73) |
| 50–59 | 2.70 (1.72–4.23)*** | 0.66 (0.43–1.02) | 1.89 (1.22–2.92)** | 0.85 (0.56–1.30) | 1.99 (1.28–3.10)** | 1.18 (0.77–1.81) |
| 60 and above | 1 (ref) | 1 (ref) | 1 (ref) | 1 (ref) | 1 (ref) | 1 (ref) |
| ***Gender*** |  |  |  |  |  |  |
| Male | 1.37 (1.11–1.68)* | 0.91 (0.73–1.13) | 1.36 (1.07–1.71)* | 0.97 (0.79–1.20) | 1.33 (1.07–1.66)* | 1.22 (0.99–1.51) |
| Female | 1 (ref) | 1 (ref) | 1 (ref) | 1 (ref) | 1 (ref) | 1 (ref) |
| ***Highest education level*** |  |  |  |  |  |  |
| Secondary school and below | 1.11 (0.74–1.67) | 0.71 (0.46–1.10) | 0.64 (0.41–1.00) | 0.97 (0.64–1.45) | 1.98 (1.30–3.04)** | 1.02 (0.68–1.53) |
| Certificate/A–Level/Diploma | 1.28 (0.97–1.69) | 0.41 (0.31–0.55)*** | 0.47 (0.35–0.64)*** | 0.71 (0.54–0.94)* | 1.43 (1.07–1.91)* | 0.89 (0.68–1.18) |
| Bachelor degree | 1.35 (1.04–1.77)* | 0.68 (0.51–0.91)* | 0.84 (0.61–1.14) | 0.85 (0.65–1.10) | 1.25 (0.94–1.66) | 0.95 (0.72–1.23) |
| Postgraduate degree | 1 (ref) | 1 (ref) | 1 (ref) | 1 (ref) | 1 (ref) | 1 (ref) |
| ***Ever delayed acceptance or refuse vaccine despite availability of vaccine service*** |  |  |  |  |  |  |
| Yes | 2.06 (1.44–2.95)*** | 2.14 (1.41–3.24)*** | 1.63 (1.03–2.56)* | 2.47 (1.70–3.59)*** | 1.46 (1.01–2.11)* | 2.48 (1.74–3.55)*** |
| No | 1 (ref) | 1 (ref) | 1 (ref) | 1 (ref) | 1 (ref) | 1 (ref) |

^a^ *P*–value : 0.077; Nagelkerke *R^2^* : 0.053

^b^ *P*–value : 0.017; Nagelkerke *R^2^* : 0.195

^c^ *P*–value: 0.729; Nagelkerke *R^2^* : 0.145

^d^ *P*–value: 0.271; Nagelkerke *R^2^* : 0.064

^e^ *P*<0.001; Nagelkerke *R^2^* : 0.086

^f^ *P*–value: 0.251; Nagelkerke *R^2^* : 0.036

| **Iran**  N=1019 | ***Required doses of COVID-19 vaccine^a^*** | ***Effectiveness threshold of COVID-19 vaccine^b^*** | ***Adverse reactions of COVID-19 vaccine^c^*** | ***Duration of COVID-19 vaccine protection^d^*** | ***Technology used in COVID-19 vaccine^e^*** | ***Producing country of COVID-19 vaccine^f^*** |
| --- | --- | --- | --- | --- | --- | --- |
| Demographics |  |  |  |  |  |  |
| ***Age group*** |  |  |  |  |  |  |
| 18–29 | 1.12 (0.66–1.92) | 2.87 (1.63–5.06*** | 2.33 (1.30–4.19)** | 0.75 (0.45–1.24) | 18.03 (2.28–142.58)** | 1.41 (0.83–2.37) |
| 30–39 | 1.17 (0.68–2.03) | 2.95 (1.64–5.32)*** | 2.32 (1.28–4.19)** | 0.71 (0.42–1.20) | 16.40 (2.05–131.06)** | 1.06 (0.62–1.79) |
| 40–49 | 1.15 (0.65–2.03) | 2.74 (1.48–5.08)** | 2.12 (1.14–3.94)* | 1.13 (0.65–1.96) | 22.77 (2.86–181.62)** | 1.38 (0.79–2.41) |
| 50–59 | 0.86 (0.47–1.59) | 2.10 (1.12–3.95)* | 1.14 (0.61–2.12) | 1.37 (0.76–2.47) | 14.96 (1.78–125.70)* | 1.10 (0.61–1.96) |
| 60 and above | 1 (ref) | 1 (ref) | 1 (ref) | 1 (ref) | 1 (ref) | 1 (ref) |
| ***Gender*** |  |  |  |  |  |  |
| Male | 1.43 (1.07–1.90)* | 0.61 (0.43–0.88)** | 0.66 (0.47–0.93)* | 0.62 (0.47–0.81)*** | 0.69 (0.45–1.07)** | 1.03 (0.78–1.37) |
| Female | 1 (ref) | 1 (ref) | 1 (ref) | 1 (ref) | 1 (ref) | 1 (ref) |
| ***Highest education level*** |  |  |  |  |  |  |
| Secondary school and below | 1.89 (0.93–3.85) | 0.44 (0.21–0.95)* | 1.26 (0.56–2.81) | 0.80 (0.41–1.58) | 3.92 (1.61–9.53)** | 0.85 (0.42–1.69) |
| Certificate/A–Level/Diploma | 2.02 (1.32–3.08)** | 0.56 (0.34–0.92)* | 1.63 (0.98–2.74) | 1.08 (0.72–1.63) | 0.47 (0.23–0.96)* | 0.82 (0.54–1.25)0 |
| Bachelor degree | 1.10 (0.77–1.56) | 1.06 (0.67–1.67) | 1.29 (0.86–1.94) | 1.06 (0.76–1.48) | 0.71 (0.43–1.17) | 1.19 (0.84–1.69) |
| Postgraduate degree | 1 (ref) | 1 (ref) | 1 (ref) | 1 (ref) | 1 (ref) | 1 (ref) |
| ***Ever delayed acceptance or refuse vaccine despite availability of vaccine service*** |  |  |  |  |  |  |
| Yes | 0.14 (0.11–0.19)*** | 0.14 (0.07–0.26)*** | 0.33 (0.21–0.53)*** | 0.36 (0.26–0.50)*** | 0.44 (0.29–0.67)*** | 0.70 (0.81–0.97)* |
| No | 1 (ref) | 1 (ref) | 1 (ref) | 1 (ref) | 1 (ref) | 1 (ref) |

^a^ *P*–value: 0.041; Nagelkerke *R^2^* : 0.227

^b^ *P*–value: 0.398; Nagelkerke *R^2^* : 0.040

^c^ *P*–value: 0.539; Nagelkerke *R^2^* : 0.096

^d^ *P*–value: 0.970; Nagelkerke *R^2^* : 0.015

^e^ *P*–value: 0.128; Nagelkerke *R^2^* : 0.103

^f^ *P*–value: 0.067; Nagelkerke *R^2^* : 0.021

| **Japan**  N=1037 | ***Required doses of COVID-19 vaccine^a^*** | ***Effectiveness threshold of COVID-19 vaccine^b^*** | ***Adverse reactions of COVID-19 vaccine^c^*** | ***Duration of COVID-19 vaccine protection^d^*** | ***Technology used in COVID-19 vaccine^e^*** | ***Producing country of COVID-19 vaccine^f^*** |
| --- | --- | --- | --- | --- | --- | --- |
| Demographics |  |  |  |  |  |  |
| ***Age group*** |  |  |  |  |  |  |
| 18–29 | 1.56 (0.99–2.45) | 1.59 (1.05–2.42)* | 0.93 (0.54–1.59) | 1.03 (0.68–1.54) | 2.27 (1.15–4.46)* | 0.88 (0.58–1.35) |
| 30–39 | 1.79 (1.17–2.75)** | 1.32 (0.89–1.97) | 0.91 (0.54–1.54) | 1.06 (0.72–1.57) | 1.57 (0.79–3.15) | 1.10 (0.72–1.68) |
| 40–49 | 1.27 (0.83–1.94) | 0.93 (0.64–1.36) | 1.22 (0.70–2.10) | 0.89 (0.61–1.30) | 1.84 (0.95–3.59) | 1.34 (0.88–2.05) |
| 50–59 | 1.25 (0.80–1.95) | 0.75 (0.50–1.11) | 1.33 (0.73–2.41) | 0.80 (0.53–1.20) | 0.93 (0.41–2.10) | 1.22 (0.78–1.92) |
| 60 and above | 1 (ref) | 1 (ref) | 1 (ref) | 1 (ref) | 1 (ref) | 1 (ref) |
| ***Gender*** |  |  |  |  |  |  |
| Male | 0.64 (0.48–0.87)** | 0.62 (0.48–0.81)*** | 0.65 (0.45–0.94)* | 0.70 (0.54–0.92)* | 0.93 (0.62–1.48) | 0.59 (0.45–0.79)*** |
| Female | 1 (ref) | 1 (ref) | 1 (ref) | 1 (ref) | 1 (ref) | 1 (ref) |
| ***Highest education level*** |  |  |  |  |  |  |
| Secondary school and below | 3.19 (1.93–5.27)*** | 1.12 (0.73–1.70) | 0.78 (0.43–1.43) | 1.66 (1.09–2.54)* | 0.46 (0.24–0.86)* | 1.08 (0.70–1.68) |
| Certificate/A–Level/Diploma | 2.77 (1.64–4.69)*** | 1.60 (1.01–2.53)* | 0.94 (0.48–1.84) | 1.31 (0.83–2.05) | 0.45 (0.23–0.90)* | 1.62 (0.99–2.66) |
| Bachelor degree | 2.04 (1.28–3.24)** | 0.90 (0.62–1.30) | 0.73 (0.43–1.23) | 1.11 (0.76–1.62) | 0.39 (0.23–0.67)** | 1.24 (0.85–1.83) |
| Postgraduate degree | 1 (ref) | 1 (ref) | 1 (ref) | 1 (ref) | 1 (ref) | 1 (ref) |
| ***Ever delayed acceptance or refuse vaccine despite availability of vaccine service*** |  |  |  |  |  |  |
| Yes | 2.06 (1.43–2.97)*** | 1.23 (0.86–1.77) | 1.33 (0.78–2.27) | 2.18 (1.54–3.07)*** | 2.11 (1.27–3.49)** | 0.87 (0.60–1.27) |
| No | 1 (ref) | 1 (ref) | 1 (ref) | 1 (ref) | 1 (ref) | 1 (ref) |

^a^ *P*–value: 0.769; Nagelkerke *R^2^*: 0.072

^b^ *P*–value: 0.558; Nagelkerke *R^2^*: 0.048

^c^ *P*–value: 0.720; Nagelkerke *R^2^*: 0.022

^d^ *P*–value: 0.115; Nagelkerke *R^2^*: 0.048

^e^ *P*–value: 0.272; Nagelkerke *R^2^*: 0.069

^f^ *P*–value: 0.558; Nagelkerke *R^2^*: 0.038

| **Malaysia**  N=2175 | ***Required doses of COVID-19 vaccine^a^*** | ***Effectiveness threshold of COVID-19 vaccine^b^*** | ***Adverse reactions of COVID-19 vaccine^c^*** | ***Duration of COVID-19 vaccine protection^d^*** | ***Technology used in COVID-19 vaccine^e^*** | ***Producing country of COVID-19 vaccine^f^*** |
| --- | --- | --- | --- | --- | --- | --- |
| Demographics |  |  |  |  |  |  |
| ***Age group*** |  |  |  |  |  |  |
| 18–29 | 1.27 (0.90–1.78) | 1.58 (1.11–2.26)* | 0.70 (0.48–1.02) | 0.66 (0.48–0.91)* | 0.20 (0.13–0.32)*** | 0.57 (0.42–0.76)*** |
| 30–39 | 1.33 (0.97–1.83) | 1.55 (1.12–2.14)* | 0.98 (0.68–1.41) | 0.70 (0.52–0.96)* | 0.38 (0.27–0.55)*** | 0.69 (0.52–0.92)* |
| 40–49 | 1.19 (0.86–1.66) | 1.49 (1.07–2.07)* | 0.75 (0.53–1.08) | 0.68 (0.50–0.93)* | 0.34 (0.24–0.50)*** | 0.77 (0.58–1.03) |
| 50–59 | 0.99 (0.68–1.42) | 1.53 (1.06–2.20)* | 0.73 (0.50–1.08) | 0.87 (0.61–1.23) | 0.54 (0.37–0.79)** | 0.73 (0.54–0.99)* |
| 60 and above | 1 (ref) | 1 (ref) | 1 (ref) | 1 (ref) | 1 (ref) | 1 (ref) |
| ***Gender*** |  |  |  |  |  |  |
| Male | 0.90 (0.73–1.11) | 0.58 (0.47–0.72)*** | 0.87 (0.70–1.09) | 0.79 (0.65–0.96)* | 1.00 (0.77–1.29) | 0.73 (0.61–0.88)** |
| Female | 1 (ref) | 1 (ref) | 1 (ref) | 1 (ref) | 1 (ref) | 1 (ref) |
| ***Highest education level*** |  |  |  |  |  |  |
| Secondary school and below | 3.84 (2.77–5.33)*** | 1.93 (1.33–2.80)** | 2.57 (1.73–3.83)*** | 1.37 (0.99–1.87) | 0.81 (0.54–1.22) | 1.64 (1.22–2.19)** |
| Certificate/A–Level/Diploma | 1.73 (1.26–2.37)** | 1.39 (1.01–1.90)* | 1.34 (0.98–1.84) | 1.12 (0.84–1.49) | 0.56 (0.38–0.84)** | 1.13 (0.87–1.47) |
| Bachelor degree | 1.43 (1.06–1.92)** | 1.26 (0.95–1.66) | 1.36 (1.03–1.80)* | 1.04 (0.81–1.34) | 1.03 (0.75–1.42) | 1.15 (0.91–1.46) |
| Postgraduate degree | 1 (ref) | 1 (ref) | 1 (ref) | 1 (ref) | 1 (ref) | 1 (ref) |
| ***Ever delayed acceptance or refuse vaccine despite availability of vaccine service*** |  |  |  |  |  |  |
| Yes | 3.11 (2.28–4.24)*** | 1.82 (1.17–2.82)** | 1.96 (1.23–3.10)** | 2.07 (1.41–3.05)*** | 2.99 (2.12–4.23)*** | 1.93 (1.40–2.65)*** |
| No | 1 (ref) | 1 (ref) | 1 (ref) | 1 (ref) | 1 (ref) | 1 (ref) |

^a^ *P*–value: 0.273; Nagelkerke *R^2^* : 0.089

^b^ *P*–value: 0.839; Nagelkerke *R^2^* : 0.047

^c^ *P*–value: 0.895; Nagelkerke *R^2^* : 0.032

^d^ *P*–value: 0.580; Nagelkerke *R^2^*: 0.023

^e^ *P*–value: 0.752; Nagelkerke *R^2^*: 0.093

^f^ *P*–value: 0.101; Nagelkerke *R^2^*: 0.034

| **Norway**  N=1382 | ***Required doses of COVID-19 vaccine^a^*** | ***Effectiveness threshold of COVID-19 vaccine^b^*** | ***Adverse reactions of COVID-19 vaccine^c^*** | ***Duration of COVID-19 vaccine protection^d^*** | ***Technology used in COVID-19 vaccine^e^*** | ***Producing country of COVID-19 vaccine^f^*** |
| --- | --- | --- | --- | --- | --- | --- |
| Socio demography |  |  |  |  |  |  |
| ***Age group*** |  |  |  |  |  |  |
| 18–29 | 1.10 (0.64–1.90) | 0.47 (0.30–0.76)** | 0.43 (0.27–0.69)*** | 2.81 (1.76–4.49)*** | 0.36 (0.18–0.72)** | 1.82 (0.95–3.51) |
| 30–39 | 1.37 (0.82–2.30) | 0.23 (0.15–0.35)*** | 0.75 (0.49–1.13) | 5.32 (3.41–8.30)*** | 0.63 (0.33–1.21) | 0.49 (0.27–0.89)* |
| 40–49 | 0.95 (0.55–1.64) | 0.40 (0.25–0.63)*** | 2.12 (1.37–3.28)** | 2.58 (1.63–4.07)*** | 2.30 (1.24–4.26)** | 0.49 (0.26–0.92)* |
| 50–59 | 0.71 (0.41–1.23) | 0.50 (0.31–0.81)* | 4.74 (2.95–7.60)*** | 5.81 (3.62–9.33)*** | 2.21 (1.18–4.15)* | 0.47 (0.25–0.89)* |
| 60 and above | 1 (ref) | 1 (ref) | 1 (ref) | 1 (ref) | 1 (ref) | 1 (ref) |
| ***Gender*** |  |  |  |  |  |  |
| Male | 0.51 (0.39–0.67) | 0.83 (0.65–1.05) | 0.41 (0.32–0.54)*** | 0.81 (0.64–1.03) | 0.56 (0.40–0.79)** | 0.83 (0.61–1.14) |
| Female | 1 (ref) | 1 (ref) | 1 (ref) | 1 (ref) | 1 (ref) | 1 (ref) |
| ***Highest education level*** |  |  |  |  |  |  |
| Secondary school and below | 8.17 (4.27–15.65)*** | 1.31 (0.72–2.39) | 1.13 (0.57–2.24) | 1.64 (0.88–3.04) | 0.24 (0.09–0.64)** | 0.22 (0.17–0.43)*** |
| Certificate/A–Level/Diploma | 1.62 (1.07–2.44)* | 2.47 (1.81–3.37)*** | 0.64 (0.46–0.88)** | 0.73 (0.53–0.98)* | 0.28 (0.17–0.45)*** | 2.10 (1.28–3.44)** |
| Bachelor degree | 8.26 (5.66–12.05)*** | 4.04 (3.00–5.47)*** | 0.41 (0.30–0.57)*** | 0.65 (0.49–0.87)** | 1.33 (0.89–2.00) | 0.22 (0.15–0.33)*** |
| Postgraduate degree | 1 (ref) | 1 (ref) | 1 (ref) | 1 (ref) | 1 (ref) | 1 (ref) |
| ***Ever delayed acceptance or refuse vaccine despite availability of vaccine service*** |  |  |  |  |  |  |
| Yes | 3.66 (2.40–5.56)*** | 1.33 (0.88–2.00) | 2.75 (1.78–4.26)*** | 2.00 (1.35–2.96)** | 4.01 (0.257–6.26)*** | 0.31 (0.30–0.49)*** |
| No | 1 (ref) | 1 (ref) | 1 (ref) | 1 (ref) | 1 (ref) | 1 (ref) |

^a^ *P*<0.001; Nagelkerke *R^2^*: 0.281

^b^ *P*–value: 0.007; Nagelkerke *R^2^*: 0.149

^c^ *P*<0.001; Nagelkerke *R^2^*: 0.256

^d^ *P*<0.001; Nagelkerke *R^2^*: 0.101

^e^ *P*<0.001; Nagelkerke *R^2^*: 0.207

^f^ *P*<0.001; Nagelkerke *R^2^*: 0.249

| **Pakistan**  N=1271 | ***Required doses of COVID-19 vaccine^a^*** | ***Effectiveness threshold of COVID-19 vaccine^b^*** | ***Adverse reactions of COVID-19 vaccine^c^*** | ***Duration of COVID-19 vaccine protection^d^*** | ***Technology used in COVID-19 vaccine^e^*** | ***Producing country of COVID-19 vaccine^f^*** |
| --- | --- | --- | --- | --- | --- | --- |
| Demographics |  |  |  |  |  |  |
| ***Age group*** |  |  |  |  |  |  |
| 18–29 | 2.09 (1.30–3.34)** | 3.45 (2.09–5.69)*** | 1.19 (0.69–2.03) | 2.45 (1.57–3.82)*** | 3.04 (1.52–6.08)** | 2.05 (1.32–3.19)** |
| 30–39 | 1.40 (0.86–2.26) | 2.12 (1.28–3.51)** | 0.84 (0.49–1.45) | 1.44 (0.92–2.27) | 2.74 (1.35–5.58)** | 2.78 (1.74–4.42)*** |
| 40–49 | 0.74 (0.43–1.27) | 1.11 (0.65–1.90) | 0.52 (0.29–0.93)* | 0.92 (0.55–1.52) | 1.10 (0.47–2.58) | 1.13 (0.69–1.86) |
| 50–59 | 1.86 (1.11–3.13)* | 4.12 (2.26–7.52)*** | 0.86 (0.48–1.530 | 1.78 (1.09–2.90)* | 2.09 (0.96–4.55) | 1.36 (0.84–2.19) |
| 60 and above | 1 (ref) | 1 (ref) | 1 (ref) | 1 (ref) | 1 (ref) | 1 (ref) |
| ***Gender*** |  |  |  |  |  |  |
| Male | 1.11 (0.87–1.40) | 0.93 (0.73–1.19) | 0.84 (0.65–1.09) | 1.34 (1.06–1.69)* | 0.90 (0.66–1.22) | 1.15 (0.92–1.45) |
| Female | 1 (ref) | 1 (ref) | 1 (ref) | 1 (ref) | 1 (ref) | 1 (ref) |
| ***Highest education level*** |  |  |  |  |  |  |
| Secondary school and below | 2.55 (1.64–3.97)*** | 1.84 (1.13–2.99)* | 1.15 (0.71–1.88) | 0.75 (0.49–1.15) | 0.29 (0.16–0.54)*** | 1.41 (0.92–2.15) |
| Certificate/A–Level/Diploma | 1.12 (0.68–1.83) | 0.87 (0.51–1.46) | 2.07 (1.06–4.07)* | 0.38 (0.23–0.62)*** | 0.87 (0.49–1.55) | 1.45 (0.89–2.38) |
| Bachelor degree | 1.01 (0.75–1.35) | 0.66 (0.49–0.90)** | 0.64 (0.47–0.87)** | 0.71 (0.53–0.95)* | 0.83 (0.58–1.18) | 1.27 (0.95–1.70) |
| Postgraduate degree | 1 (ref) | 1 (ref) | 1 (ref) | 1 (ref) | 1 (ref) | 1 (ref) |
| ***Ever delayed acceptance or refuse vaccine despite availability of vaccine service*** |  |  |  |  |  |  |
| Yes | 3.05 (2.32–4.01)*** | 1.84 (1.37–2.47)*** | 2.42 (1.74–3.37)*** | 2.39 (1.81–3.14)*** | 3.20 (2.28–4.49)*** | 1.78 (1.36–2.33)*** |
| No | 1 (ref) | 1 (ref) | 1 (ref) | 1 (ref) | 1 (ref) | 1 (ref) |

^a^ *P*–value: 0.071; Nagelkerke *R^2^*: 0.165

^b^ *P*–value: 0.002; Nagelkerke *R^2^*: 0.100

^c^ *P*–value: 0.018; Nagelkerke *R^2^* : 0.100

^d^ *P*–value: 0.409; Nagelkerke *R^2^*: 0.097

^e^ *P*–value: 0.028; Nagelkerke *R^2^*: 0.138

^f^ *P*–value: 0.079; Nagelkerke *R^2^*: 0.056

| **Singapore**  N=841 | ***Required doses of COVID-19 vaccine^a^*** | ***Effectiveness threshold of COVID-19 vaccine^b^*** | ***Adverse reactions of COVID-19 vaccine^c^*** | ***Duration of COVID-19 vaccine protection^d^*** | ***Technology used in COVID-19 vaccine^e^*** | ***Producing country of COVID-19 vaccine^f^*** |
| --- | --- | --- | --- | --- | --- | --- |
| Demographics |  |  |  |  |  |  |
| ***Age group*** |  |  |  |  |  |  |
| 18–29 | 0.54 (0.22–1.34) | 1.07 (0.54–2.12) | 0.74 (0.38–1.43) | 1.24 (0.65–2.36) | 0.23 (0.05–1.12) | 2.10 (1.12–3.93)* |
| 30–39 | 0.48 (0.23–0.97)* | 1.29 (0.75–2.21) | 0.95 (0.56–1.61) | 1.08 (0.66–1.77) | 0.35 (0.14–0.87)* | 1.92 (1.18–3.13)** |
| 40–49 | 0.60 (0.31–1.15) | 1.27 (0.75–2.18) | 1.14 (0.67–1.950 | 1.14 (0.70–1.87) | 0.52 (0.22–1.22) | 2.60 (1.60–4.23)*** |
| 50–59 | 0.70 (0.37–1.30) | 1.61 (0.93–2.79) | 0.92 (0.54–1.55) | 1.15 (0.71–1.86) | 0.61 (0.26–1.44) | 3.68 (2.24–6.06)*** |
| 60 and above | 1 (ref) | 1 (ref) | 1 (ref) | 1 (ref) | 1 (ref) | 1 (ref) |
| ***Gender*** |  |  |  |  |  |  |
| Male | 0.57 (0.36–0.91)* | 0.74 (0.54–1.03) | 0.73 (0.53–0.99)* | 0.77 (0.57–1.03) | 1.91 (1.06–3.47)* | 0.55 (0.41–0.74)*** |
| Female | 1 (ref) | 1 (ref) | 1 (ref) | 1 (ref) | 1 (ref) | 1 (ref) |
| ***Highest education level*** |  |  |  |  |  |  |
| Secondary school and below | 4.51 (1.88–10.80)** | 1.91 (0.95–3.85) | 1.64 (0.86–3.13) | 1.00 (0.55–1.79) | 0.23 (0.05–1.09) | 0.98 (0.54–1.79) |
| Certificate/A–Level/Diploma | 4.75 (2.20–1.028)*** | 1.41 (0.84–2.36) | 2.00 (1.20–2.32)** | 1.24 (0.78–1.98) | 1.04 (0.46–2.35) | 1.15 (0.72–1.86) |
| Bachelor degree | 2.65 (1.29–5.44)** | 1.16 (0.78–1.73) | 1.24 (0.85–1.81) | 1.10 (0.76–1.59) | 0.77 (0.38–1.55) | 0.97 (0.67–1.41) |
| Postgraduate degree | 1 (ref) | 1 (ref) | 1 (ref) | 1 (ref) | 1 (ref) | 1 (ref) |
| ***Ever delayed acceptance or refuse vaccine despite availability of vaccine service*** |  |  |  |  |  |  |
| Yes | 2.69 (1.59–4.55)*** | 1.71 (0.99–2.94) | 1.60 (0.97–2.64) | 1.74 (1.08–2.80)* | 3.73 (1.93–7.23)*** | 2.09 (1.28–3.40)** |
| No | 1 (ref) | 1 (ref) | 1 (ref) | 1 (ref) | 1 (ref) | 1 (ref) |

^a^ *P*–value: 0.674; Nagelkerke *R^2^*: 0.113

^b^ *P*–value: 0.237; Nagelkerke *R^2^*: 0.027

^c^ *P*–value: 0.248; Nagelkerke *R^2^*: 0.034

^d^ *P*–value: 0.910; Nagelkerke *R2* : 0.019

^e^ *P*–value: 0.753; Nagelkerke *R2* : 0.091

^f^ *P*–value: 0.874; Nagelkerke *R2* : 0.095

| **Sri Lanka**  N=776 | ***Required doses of COVID-19 vaccine^a^*** | ***Effectiveness threshold of COVID-19 vaccine^b^*** | ***Adverse reactions of COVID-19 vaccine^c^*** | ***Duration of COVID-19 vaccine protection^d^*** | ***Technology used in COVID-19 vaccine^e^*** | ***Producing country of COVID-19 vaccine^f^*** |
| --- | --- | --- | --- | --- | --- | --- |
| Demographics |  |  |  |  |  |  |
| ***Age group*** |  |  |  |  |  |  |
| 18–29 | 0.11 (0.40–0.31)*** | 3.58 (1.56–8.24)** | 0.44 (0.15–1.33) | 1.74 (0.76–3.99) | 0.56 (0.02–0.14)*** | 2.87 (1.23–6.55)* |
| 30–39 | 0.16 (0.06–0.46)** | 1.56 (0.65–3.71) | 0.70 (0.22–2.23) | 1.44 (0.60–3.48) | 0.11 (0.04–0.28)*** | 1.89 (0.79–4.52) |
| 40–49 | 2.00 (0.68–5.89) | 0.67 (0.29–1.52) | 1.54 (0.48–4.90) | 1.45 (0.62–3.35) | 1.13 (0.45–2.88) | 2.93 (1.27–6.78)** |
| 50–59 | 1.47 (0.50–4.31) | 0.76 (0.33–1.75) | 1.51 (0.47–4.90) | 1.40 (0.59–3.28) | 1.22 (0.47–3.15) | 2.56 (1.09–6.00)* |
| 60 and above | 1 (ref) | 1 (ref) | 1 (ref) | 1 (ref) | 1 (ref) | 1 (ref) |
| ***Gender*** |  |  |  |  |  |  |
| Male | 0.93 (0.63–1.38) | 0.56 (0.39–0.81)** | 1.18 (0.77–1.80) | 0.80 (0.56–1.13) | 1.00 (0.66–1.52) | 0.49 (0.16–1.47) |
| Female | 1 (ref) | 1 (ref) | 1 (ref) | 1 (ref) | 1 (ref) | 1 (ref) |
| ***Highest education level*** |  |  |  |  |  |  |
| Secondary school and below | 3.81 (1.01–14.34)* | 1.19 (0.38–3.74) | 3.86 (0.80–18.66) | 0.51 (0.17–1.49) | 2.06 (0.55–7.65) | 0.49 (0.16–1.47) |
| Certificate/A–Level/Diploma | 2.99 (1.62–5.52*** | 1.06 (0.62–1.80) | 2.82 (1.55–5.15)** | 0.89 (0.53–1.51) | 1.59 (0.87–2.92) | 0.68 (0.39–1.17) |
| Bachelor degree | 2.55 (1.47–4.43)** | 0.85 (0.53–1.37) | 2.51 (1.48–4.23)** | 1.14 (0.70–1.85) | 1.79 (1.03–3.11)* | 0.71 (0.43–1.16) |
| Postgraduate degree | 1 (ref) | 1 (ref) | 1 (ref) | 1 (ref) | 1 (ref) | 1 (ref) |
| ***Ever delayed acceptance or refuse vaccine despite availability of vaccine service*** |  |  |  |  |  |  |
| Yes | 1.05 (0.57–1.94) | 1.35 (0.74–2.47) | 1.13 (0.59–2.18) | 1.70 (0.91–3.18) | 0.98 (0.50–1.93) | 1.72 (0.93–3.16) |
| No | 1 (ref) | 1 (ref) | 1 (ref) | 1 (ref) | 1 (ref) | 1 (ref) |

^a^ *P*–value: 0.952; Nagelkerke *R^2^*: 0.382

^b^ *P*–value: 0.481; Nagelkerke *R^2^*: 0.148

^c^ *P*–value: 0.871; Nagelkerke *R^2^*: 0.090

^d^ *P*–value: 0.622; Nagelkerke *R^2^*: 0.021

^e^ *P*–value: 0.135; Nagelkerke *R^2^*: 0.433

^f^ *P*–value: 0.928; Nagelkerke *R^2^*: 0.027

| **Somalia**  N=894 | ***Required doses of COVID-19 vaccine^a^*** | ***Effectiveness threshold of COVID-19 vaccine^b^*** | ***Adverse reactions of COVID-19 vaccine^c^*** | ***Duration of COVID-19 vaccine protection^d^*** | ***Technology used in COVID-19 vaccine^e^*** | ***Producing country of COVID-19 vaccine^f^*** |
| --- | --- | --- | --- | --- | --- | --- |
| Demographics |  |  |  |  |  |  |
| ***Age group*** |  |  |  |  |  |  |
| 18–29 | 0.74 (0.36–1.52) | 1.03 (0.50–2.12) | 0.54 (0.25–1.19) | 0.63 (0.32–1.27) | 1.76 (0.86–3.59) | 1.87 (0.96–3.65) |
| 30–39 | 0.66 (0.33–1.33) | 0.70 (0.35–1.40) | 0.52 (0.25–1.12) | 0.97 (0.50–1.91) | 1.83 (0.91–3.66) | 1.40 (0.74–2.67) |
| 40–49 | 0.66 (0.33–1.34) | 0.51 (0.25–1.02) | 0.72 (0.33–1.54) | 0.65 (0.33–1.28) | 2.71 (1.35–5.45)** | 1.05 (0.55–2.02) |
| 50–59 | 0.54 (0.26–1.110 | 0.48 (0.23–0.98)* | 0.72 (0.33–1.58) | 0.58 (0.29–1.17) | 2.42 (1.18–4.97)* | 0.83 (0.42–1.64) |
| 60 and above | 1 (ref) | 1 (ref) | 1 (ref) | 1 (ref) | 1 (ref) | 1 (ref) |
| ***Gender*** |  |  |  |  |  |  |
| Male | 1.21 (0.92–1.59) | 0.99 (0.75–1.31) | 0.96 (0.72–1.28) | 1.10 (0.83–1.45) | 1.02 (0.78–1.34) | 1.09 (0.84–1.43) |
| Female | 1 (ref) | 1 (ref) | 1 (ref) | 1 (ref) | 1 (ref) | 1 (ref) |
| ***Highest education level*** |  |  |  |  |  |  |
| Secondary school and below | 0.77 (0.46–1.31) | 0.90 (0.51–1.56) | 0.67 (0.38–1.18) | 1.03 (0.60–1.76) | 0.38 (0.23–0.65)*** | 0.98 (0.58–1.65) |
| Certificate/A–Level/Diploma | 1.12 (0.66–1.89) | 0.66 (0.38–1.14) | 0.91 (0.52–1.59) | 0.67 (0.39–1.13) | 0.51 (0.30–0.85)* | 0.93 (0.56–1.56) |
| Bachelor degree | 1.12 (0.67–1.90) | 0.84 (0.49–1.45) | 1.23 (0.70–2.16) | 1.00 (0.59–1.70) | 0.88 (0.53–1.48) | 1.11 (0.67–1.86) |
| Postgraduate degree | 1 (ref) | 1 (ref) | 1 (ref) | 1 (ref) | 1 (ref) | 1 (ref) |
| ***Ever delayed acceptance or refuse vaccine despite availability of vaccine service*** |  |  |  |  |  |  |
| Yes | 0.95 (0.71–1.26) | 1.79 (1.33–2.40)*** | 1.37 (1.01–1.87)* | 1.84 (1.38–2.46)*** | 0.91 (0.69–1.22) | 1.19 (0.90–1.58) |
| No | 1 (ref) | 1 (ref) | 1 (ref) | 1 (ref) | 1 (ref) | 1 (ref) |

^a^ *P*–value: 0.059; Nagelkerke *R^2^*: 0.016

^b^ *P*–value: 0.692; Nagelkerke *R^2^*: 0.069

^c^ *P*–value: 0.322; Nagelkerke *R^2^*: 0.029

^d^ *P*–value: 0.780; Nagelkerke *R^2^*: 0.060

^e^ *P*–value: 0.161; Nagelkerke *R^2^*: 0.063

^f^ *P*–value: 0.135; Nagelkerke *R^2^*: 0.033

| **South Africa**  N=1086 | ***Required doses of COVID-19 vaccine^a^*** | ***Effectiveness threshold of COVID-19 vaccine^b^*** | ***Adverse reactions of COVID-19 vaccine^c^*** | ***Duration of COVID-19 vaccine protection^d^*** | ***Technology used in COVID-19 vaccine^e^*** | ***Producing country of COVID-19 vaccine^f^*** |
| --- | --- | --- | --- | --- | --- | --- |
| Demographics |  |  |  |  |  |  |
| ***Age group*** |  |  |  |  |  |  |
| 18–29 | 1.43 (0.87–2.35) | 1.04 (0.68–1.60) | 1.41 (0.92–2.15) | 1.02 (0.67–1.55) | 1.28 (0.62–2.62) | 0.63 (0.41–0.98)* |
| 30–39 | 1.56 (0.99–2.45) | 0.94 (0.64–1.39) | 0.88 (0.59–1.30) | 0.64 (0.43–0.94)* | 0.84 (0.42–1.68) | 0.41 (0.27–0.63)*** |
| 40–49 | 1.58 (0.99–2.49) | 0.83 (0.55–1.23) | 0.91 (0.61–1.35) | 0.81 (0.55–1.20) | 1.46 (0.76–2.77) | 0.45 (0.29–0.68)*** |
| 50–59 | 1.31 (0.80–2.15) | 0.73 (0.47–1.12) | 0.89 (0.58–1.36) | 0.93 (062–1.42) | 0.93 (0.45–1.92) | 0.53 (0.34–0.82)** |
| 60 and above | 1 (ref) | 1 (ref) | 1 (ref) | 1 (ref) | 1 (ref) | 1 (ref) |
| ***Gender*** |  |  |  |  |  |  |
| Male | 1.26 (0.95–1.68) | 0.98 (0.76–1.26) | 0.91 (0.70–1.17) | 0.74 (0.57–0.95)* | 1.09 (0.71–1.67) | 0.94 (0.71–1.24) |
| Female | 1 (ref) | 1 (ref) | 1 (ref) | 1 (ref) | 1 (ref) | 1 (ref) |
| ***Highest education level*** |  |  |  |  |  |  |
| Secondary school and below | 2.48 (1.55–3.97)*** | 1.32 (0.87–2.00) | 0.84 (0.56–1.26) | 1.17 (0.78–1.75) | 0.47 (0.22–0.98)* | 1.33 (0.86–2.06) |
| Certificate/A–Level/Diploma | 2.99 (2.05–4.38)*** | 1.64 (1.19–2.27)** | 0.74 (0.54–1.02) | 0.93 (0.68–1.28) | 0.68 (0.41–1.11) | 1.03 (0.72–1.46) |
| Bachelor degree | 1.72 (1.09–2.71)* | 1.24 (0.85–1.82) | 0.91 (0.63–1.32) | 1.24 (0.86–1.79) | 0.67 (0.36–1.22) | 1.29 (0.86–1.92) |
| Postgraduate degree | 1 (ref) | 1 (ref) | 1 (ref) | 1 (ref) | 1 (ref) | 1 (ref) |
| ***Ever delayed acceptance or refuse vaccine despite availability of vaccine service*** |  |  |  |  |  |  |
| Yes | 3.75 (2.32–6.06)*** | 5.27 (3.11–8.94)*** | 5.66 (3.25–9.85)*** | 3.86 (2.31–6.44)*** | 6.23 (3.68–10.58)*** | 3.44 (2.14–5.53)*** |
| No | 1 (ref) | 1 (ref) | 1 (ref) | 1 (ref) | 1 (ref) | 1 (ref) |

^a^ *P*–value: 0.687; Nagelkerke *R^2^*: 0.088

^b^ *P*–value: 0.213; Nagelkerke *R^2^*: 0.067

^c^ *P*–value: 0.125; Nagelkerke *R^2^*: 0.072

^d^ *P*–value: 0.229; Nagelkerke *R^2^*: 0.062

^e^ *P*–value: 0.203; Nagelkerke *R^2^*: 0.093

^f^ *P*–value: 0.734; Nagelkerke *R^2^*: 0.068

| **United Arab Emirates**  N=938 | ***Required doses of COVID-19 vaccine^a^*** | ***Effectiveness threshold of COVID-19 vaccine^b^*** | ***Adverse reactions of COVID-19 vaccine^c^*** | ***Duration of COVID-19 vaccine protection^d^*** | ***Technology used in COVID-19 vaccine^e^*** | ***Producing country of COVID-19 vaccine^f^*** |
| --- | --- | --- | --- | --- | --- | --- |
| Demographics |  |  |  |  |  |  |
| ***Age group*** |  |  |  |  |  |  |
| 18–29 | 0.89 (0.51–1.55) | 0.76 (0.43–1.35) | 0.50 (0.24–1.04) | 0.75 (0.43–1.32) | 1.11 (0.62–2.02) | 1.09 (0.63–1.90) |
| 30–39 | 0.82 (0.49–1.37) | 0.69 (0.41–1.17) | 0.52 (0.26–1.02) | 0.91 (0.54–1.54) | 1.14 (0.66–1.98) | 1.02 (0.61–1.70) |
| 40–49 | 0.80 (0.47–1.37) | 0.57 (0.33–0.97)* | 0.44 (0.22–0.87)* | 0.67 (0.41–1.17) | 1.06 (0.61–1.84) | 0.83 (0.50–1.40) |
| 50–59 | 0.88 (0.49–1.57) | 0.63 (0.35–1.13) | 0.47 (0.23–0.98)* | 0.72 (0.40–1.28) | 0.60 (0.32–0.95) | 0.81 (0.46–1.43) |
| 60 and above | 1 (ref) | 1 (ref) | 1 (ref) | 1 (ref) | 1 (ref) | 1 (ref) |
| ***Gender*** |  |  |  |  |  |  |
| Male | 0.97 (0.74–1.27) | 0.92 (0.70–1.20) | 0.97 (0.71–1.31) | 1.01 (0.77–1.32) | 1.02 (0.77–1.36) | 1.26 (0.97–1.64) |
| Female | 1 (ref) | 1 (ref) | 1 (ref) | 1 (ref) | 1 (ref) | 1 (ref) |
| ***Highest education level*** |  |  |  |  |  |  |
| Secondary school and below | 2.10 (1.24–3.56)** | 3.00 (1.78–5.05)*** | 2.65 (1.52–4.60)** | 2.38 (1.41–4.01)** | 0.56 (0.33–0.95)* | 1.84 (1.09–3.10)* |
| Certificate/A–Level/Diploma | 2.01 (1.19–3.39)** | 1.92 (1.5–3.20)* | 2.24 (1.31–3.84)** | 1.93 (1.15–3.23)* | 0.70 (0.42–1.18) | 1.75 (1.04–2.94)* |
| Bachelor degree | 0.86 (0.52–1.43) | 1.22 (0.75–1.98) | 1.79 (1.09–2.94)* | 1.28 (0.78–2.08) | 0.49 (0.30–0.81)** | 1.81 (1.11–2.96)* |
| Postgraduate degree | 1 (ref) | 1 (ref) | 1 (ref) | 1 (ref) | 1 (ref) | 1 (ref) |
| ***Ever delayed acceptance or refuse vaccine despite availability of vaccine service*** |  |  |  |  |  |  |
| Yes | 0.77 (0.58–1.03) | 1.08 (0.82–1.42) | 1.30 (0.94–1.78) | 1.53 (1.16–2.01)** | 1.13 (0.84–1.52) | 1.08 (0.82–1.42) |
| No | 1 (ref) | 1 (ref) | 1 (ref) | 1 (ref) | 1 (ref) | 1 (ref) |

^a^ *P*–value: 0.782; Nagelkerke *R^2^*: 0.056

^b^ *P*–value: 0.395; Nagelkerke *R^2^*: 0.062

^c^ *P*–value: 0.759; Nagelkerke *R^2^*: 0.040

^d^ *P*–value: 0.647; Nagelkerke *R^2^*: 0.057

^e^ *P*–value: 0.894; Nagelkerke *R^2^*: 0.023

^f^ *P*–value: 0.545; Nagelkerke *R^2^*: 0.023

| **United Kingdom**  N=1021 | ***Required doses of COVID-19 vaccine^a^*** | ***Effectiveness threshold of COVID-19 vaccine^b^*** | ***Adverse reactions of COVID-19 vaccine^c^*** | ***Duration of COVID-19 vaccine protection^d^*** | ***Technology used in COVID-19 vaccine^e^*** | ***Producing country of COVID-19 vaccine^f^*** |
| --- | --- | --- | --- | --- | --- | --- |
| Demographics |  |  |  |  |  |  |
| ***Age group*** |  |  |  |  |  |  |
| 18–29 | 1.23 (0.63–2.42) | 1.14 (0.60–2.16) | 1.25 (0.58–2.69) | 0.32 (0.16–0.64)** | 1.12 (0.38–3.34) | 0.84 (0.39–1.81) |
| 30–39 | 0.79 (0.41–1.53) | 0.89 (0.47–1.66) | 2.28 (1.06–4.90)* | 0.38 (0.19–0.74)* | 1.04 (0.36–3.03) | 0.74 (0.35–1.56) |
| 40–49 | 1.27 (0.63–2.54) | 0.79 (0.41–1.53) | 1.81 (0.81–4.04) | 0.52 (0.26–1.05) | 1.85 (0.63–5.42) | 0.63 (0.29–1.37) |
| 50–59 | 1.34 (0.66–2.73) | 1.31 (0.66–2.59) | 2.65 (1.10–6.34)* | 0.59 (0.29–1.23) | 1.71 (0.56–5.23) | 0.67 (0.30–1.52) |
| 60 and above | 1 (ref) | 1 (ref) | 1 (ref) | 1 (ref) | 1 (ref) | 1 (ref) |
| ***Gender*** |  |  |  |  |  |  |
| Male | 1.96 (1.49–2.57)*** | 1.28 (0.99–1.65) | 1.52 (1.07–2.16)* | 1.76 (1.36–2.28)*** | 0.68 (0.44–1.05) | 1.35 (1.01–1.82)* |
| Female | 1 (ref) | 1 (ref) | 1 (ref) | 1 (ref) | 1 (ref) | 1 (ref) |
| ***Highest education level*** |  |  |  |  |  |  |
| Secondary school and below | 0.86 (0.16–4.57) | 0.54 (0.12–2.46) | 0.98 (0.11–8.67) | 2.26 (0.43–11.93) | 1.67 (0.19–14.48) | 0.23 (0.05–1.05) |
| Certificate/A–Level/Diploma | 1.72 (1.23–2.40)** | 1.27 (0.92–1.75) | 1.13 (0.72–1.79) | 1.58 (1.15–2.17)** | 0.24 (0.12–0.48)*** | 0.67 (0.45–0.98)* |
| Bachelor degree | 1.65 (1.19–2.28)** | 0.78 (0.58–1.06) | 0.72 (0.48–1.10) | 1.16 (0.85–1.57) | 0.94 (0.58–1.50) | 0.46 (0.32–0.66)*** |
| Postgraduate degree | 1 (ref) | 1 (ref) | 1 (ref) | 1 (ref) | 1 (ref) | 1 (ref) |
| ***Ever delayed acceptance or refuse vaccine despite availability of vaccine service*** |  |  |  |  |  |  |
| Yes | 0.89 (0.55–1.45) | 0.68 (0.43–1.07) | 1.23 (0.65–2.34) | 0.95 (0.60–1.51) | 5.16 (2.95–9.02)*** | 0.32 (0.20–0.51)*** |
| No | 1 (ref) | 1 (ref) | 1 (ref) | 1 (ref) | 1 (ref) | 1 (ref) |

^a^ *P*–value: 0.003; Nagelkerke *R^2^*: 0.055

^b^ *P*<0.001; Nagelkerke *R^2^*: 0.031

^c^ *P*–value: 0.038; Nagelkerke *R^2^*: 0.037

^d^ *P*–value: 0.002; Nagelkerke *R^2^*: 0.052

^e^ *P*–value: 0.150; Nagelkerke *R^2^*: 0.122

^f^ *P*–value: 0.147; Nagelkerke *R^2^*: 0.116

| **United States of America**  N=968 | ***Required doses of COVID-19 vaccine^a^*** | ***Effectiveness threshold of COVID-19 vaccine^b^*** | ***Adverse reactions of COVID-19 vaccine^c^*** | ***Duration of COVID-19 vaccine protection^d^*** | ***Technology used in COVID-19 vaccine^e^*** | ***Producing country of COVID-19 vaccine^f^*** |
| --- | --- | --- | --- | --- | --- | --- |
| Demographics |  |  |  |  |  |  |
| ***Age group*** |  |  |  |  |  |  |
| 18–29 | 4.07 (2.31–7.17)*** | 1.34 (0.81–2.21) | 1.07 (0.63–1.80) | 2.09 (1.24–3.50)** | 1.54 (0.80–2.98) | 1.96 (1.16–3.44)* |
| 30–39 | 4.22 (2.52–7.09)*** | 1.35 (0.87–2.12) | 0.75 (0.47–1.19) | 2.12 (1.34–3.34)** | 1.43 (0.79–2.60) | 2.22 (1.38–3.59)** |
| 40–49 | 4.97 (2.92–8.47)*** | 1.05 (0.66–1.67) | 0.63 (0.39–1.01) | 2.89 (1.76–4.67)*** | 1.85 (1.01–3.39)* | 2.33 (1.41–3.86)** |
| 50–59 | 2.62 (1.45–4.73)** | 1.32 (0.78–2.23) | 0.57 (0.33–0.98)* | 1.72 (1.01–2.94)* | 1.50 (0.76–2.98) | 2.13 (1.20–3.78)* |
| 60 and above | 1 (ref) | 1 (ref) | 1 (ref) | 1 (ref) | 1 (ref) | 1 (ref) |
| ***Gender*** |  |  |  |  |  |  |
| Male | 0.79 (0.60–1.04) | 0.80 (0.61–1.05) | 0.79 (0.60–1.04) | 1.07 (0.80–1.43) | 1.23 (0.87–1.71) | 1.01 (0.73–1.38) |
| Female | 1 (ref) | 1 (ref) | 1 (ref) | 1 (ref) | 1 (ref) | 1 (ref) |
| ***Highest education level*** |  |  |  |  |  |  |
| Secondary school and below | 0.98 (0.54–1.79) | 1.68 (0.95–2.98) | 1.57 (0.86–2.85) | 0.67 (0.37–1.22) | 1.03 (0.53–2.02) | 0.56 (0.31–1.03) |
| Certificate/A–Level/Diploma | 1.46 (0.88–2.41) | 1.50 (0.92–2.42) | 1.04 (0.63–1.72) | 1.11 (0.66–1.87) | 0.81 (0.46–1.44) | 1.16 (0.69–1.95) |
| Bachelor degree | 1.59 (0.98–2.59) | 1.16 (0.73–1.85) | 0.88 (0.54–1.43) | 0.96 (0.58–1.58) | 0.69 (0.40–1.20) | 1.83 (1.09–3.05)* |
| Postgraduate degree | 1 (ref) | 1 (ref) | 1 (ref) | 1 (ref) | 1 (ref) | 1 (ref) |
| ***Ever delayed acceptance or refuse vaccine despite availability of vaccine service*** |  |  |  |  |  |  |
| Yes | 0.99 (0.68–1.45) | 1.93 (1.32–2.82)** | 5.52 (3.47–8.77)*** | 0.95 (0.64–1.41) | 1.67 (1.09–2.54)* | 0.65 (0.44–0.98)* |
| No | 1 (ref) | 1 (ref) | 1 (ref) | 1 (ref) | 1 (ref) | 1 (ref) |

^a^ *P*–value: 0.862; Nagelkerke *R^2^*: 0.093

^b^ *P*–value: 0.267; Nagelkerke *R^2^*: 0.034

^c^ *P*–value: 0.957; Nagelkerke *R^2^*: 0.113

^d^ *P*–value: 0.625; Nagelkerke *R^2^*: 0.037

^e^ *P*–value: 0.415; Nagelkerke *R^2^*: 0.023

^f^ *P*–value: 0.323; Nagelkerke *R^2^*: 0.075

| **Vietnam**  N=1462 | ***Required doses of COVID-19 vaccine^a^*** | ***Effectiveness threshold of COVID-19 vaccine^b^*** | ***Adverse reactions of COVID-19 vaccine^c^*** | ***Duration of COVID-19 vaccine protection^d^*** | ***Technology used in COVID-19 vaccine^e^*** | ***Producing country of COVID-19 vaccine^f^*** |
| --- | --- | --- | --- | --- | --- | --- |
| Demographics |  |  |  |  |  |  |
| ***Age group*** |  |  |  |  |  |  |
| 18–29 | 0.33 (0.19–0.58)*** | 0.71 (0.37–1.35) | 0.52 (0.26–1.01) | 0.48 (0.27–0.86)* | 0.42 (0.24–0.75)** | 1.14 (0.65–2.00) |
| 30–39 | 1.11 (0.65–1.89) | 1.02 (0.56–1.87) | 0.53 (0.28–0.99)* | 0.77 (0.45–1.33) | 1.05 (0.63–1.76) | 1.14 (0.68–1.92) |
| 40–49 | 0.78 (0.44–1.38) | 0.83 (0.44–1.57) | 0.50 (0.26–0.97) | 0.76 (0.43–1.36) | 1.19 (0.69–2.06) | 1.05 (0.60–1.83) |
| 50–59 | 1.59 (0.84–3.00) | 1.25 (0.62–2.53) | 0.54 (0.37–1.09) | 0.91 (0.49–1.69) | 1.18 (0.66–2.10) | 1.21 (0.67–2.20) |
| 60 and above | 1 (ref) | 1 (ref) | 1 (ref) | 1 (ref) | 1 (ref) | 1 (ref) |
| ***Gender*** |  |  |  |  |  |  |
| Male | 0.99 (0.79–1.24) | 0.84 (0.64–1.09) | 0.90 (0.71–1.14) | 1.01 (0.81–1.26) | 1.13 (0.90–1.43) | 1.11 (0.88–1.39) |
| Female | 1 (ref) | 1 (ref) | 1 (ref) | 1 (ref) | 1 (ref) | 1 (ref) |
| ***Highest education level*** |  |  |  |  |  |  |
| Secondary school and below | 1.90 (1.21–2.97)** | 1.25 (0.74–2.11) | 0.95 (0.58–1.57) | 1.09 (0.70–1.72) | 1.03 (0.65–1.61) | 1.07 (0.68–1.68) |
| Certificate/A-Level/Diploma | 2.04 (1.36–3.06)** | 0.91 (0.57–1.47) | 0.69 (0.44–1.08) | 0.88 (0.58–1.32) | 1.23 (0.82–1.86) | 0.98 (0.65–1.49) |
| Bachelor degree | 1.68 (1.11–2.55)* | 1.41 (0.85–2.34) | 0.77 (0.49–1.23) | 0.89 (0.58–1.35) | 1.22 (0.79–1.88) | 0.95 (0.62–1.46) |
| Postgraduate degree | 1 (ref) | 1 (ref) | 1 (ref) | 1 (ref) | 1 (ref) | 1 (ref) |
| ***Ever delayed acceptance or refuse vaccine despite availability of vaccine service*** |  |  |  |  |  |  |
| Yes | 0.44 (0.28–0.69)*** | 3.27 (1.61–6.65)** | 1.81 (1.09–3.00)* | 0.82 (0.54–1.23) | 0.70 (0.42–1.16) | 1.28 (0.82–2.01) |
| No | 1 (ref) | 1 (ref) | 1 (ref) | 1 (ref) | 1 (ref) | 1 (ref) |

^a^ *P*–value: 0.808; Nagelkerke *R^2^*: 0.139

^b^ *P*–value: 0.939; Nagelkerke *R^2^*: 0.029

^c^ *P*–value: 0.199; Nagelkerke *R^2^*: 0.018

^d^ *P*–value: 0.945; Nagelkerke *R^2^*: 0.029

^e^ *P*–value: 0.138; Nagelkerke *R^2^*: 0.058

^f^ *P*–value: 0.071; Nagelkerke *R^2^*: 0.003
